# Ferulic Acid Alleviates Atherosclerotic Plaques by Inhibiting VSMC Proliferation Through the NO/p21 Signaling pathway

**DOI:** 10.1007/s12265-021-10196-8

**Published:** 2022-01-06

**Authors:** Xiaoyun Wu, Ziwei Hu, Junjie Zhou, Jin Liu, Ping Ren, Xi Huang

**Affiliations:** 1 Medical College, Xiamen University, Xiamen, 361102 China; 2 School of Basic Medicine, Gannan Medical University, Ganzhou, 341000 China; 3 School of Rehabilitation Medicine, Gannan Medical University, Ganzhou, 341000 China; 4 Institute of TCM-related Comorbid Depression, Nanjing University of Chinese Medicine, 138 Xianlin Rd., 210023 Nanjing, China

**Keywords:** Atherosclerosis, Ferulic acid, NO/p21, VSMC

## Abstract

The benefits and risks of inhibiting the proliferation and migration of vascular smooth muscle cells (VSMCs) in atherosclerosis (AS) remain a subject of debate. In this study, we investigated the effect of ferulic acid (FA) on the proliferation and migration of VSMCs induced by platelet-derived growth factor (PDGF) and the associated mechanism and used ApoE^-/-^ mice to study whether the effect of FA on VSMC proliferation and migration is beneficial in alleviating AS plaques. It was found that FA not only reduced blood lipid levels but also promoted the production of nitric oxide (NO) by MOVAS cells through the endothelial nitric oxide synthase (eNOS) pathway, inhibited the migration and proliferation of VSMCs induced by PDGF, promoted the expression of p21 in VSMCs, and exerted a therapeutic effect against AS.

**Supplementary Information:**

The online version contains supplementary material available at 10.1007/s12265-021-10196-8.

## Introduction

Atherosclerosis (AS) is the pathological basis of cardiovascular and cerebrovascular diseases and the main cause of disease and disability in the world [1]. Excessive proliferation and migration of vascular smooth muscle cells (VSMCs) are closely related to the occurrence and development of AS [2]; however, the exact function of VSMCs in the pathogenesis of AS is currently unclear, and the benefits and risks of inhibiting the proliferation and migration of VSMCs in AS are still topics of debate, and more experimental evidence is needed.

Ferulic acid (FA) (4-hydroxy-3-methoxycinnamic acid) is a phenolic acid that is abundant in fruits and vegetables [3]. It is found in carrots, tomatoes, sweet potatoes, apples, peaches, plums, oilseeds, and coffee. In addition, it is also widely found in some Chinese herbal medicines, such as Ligusticum chuanxiong Angelica. Recently, FA has attracted wide attention for its nutritional and pharmacological effects, such as its antidiabetic, antihypertensive, and antitumor effects [4]. FA is also believed to have a variety of antiatherosclerotic activities, including lipid-lowering, antioxidant, and anti-inflammatory activities [5]. Although FA has a variety of promising antiatherosclerotic effects, little research has been done on cellular VSMCs.

VSMC proliferation is associated with numerous factors, such as platelets, inflammatory factors, and damaged vascular cells [6]. Studies have shown that the migration of VSMCs from the arterial media to the arterial intima leads to an increase in the generation of foam cells, which are the main causes of atherosclerotic lesions [7]. Therefore, by inhibiting the proliferation and migration of VSMCs, the number of foam cells generated from smooth muscle cells can be reduced, which is conducive to preventing the occurrence and development of AS atherosclerotic plaques and is beneficial for the treatment of AS [8]. However, it has also been proposed that VSMC proliferation and migration play an important role in maintaining the stability of plaques and are important compensatory mechanisms of plaque injury [9]. Therefore, the advantages and disadvantages of therapeutic inhibition of VSMC proliferation and migration in AS are still very controversial, and more experimental evidence is needed.

The aim of this study was to investigate the effects of FA on the proliferation and migration of platelet-derived growth factor (PDGF)-induced VSMCs and its possible mechanism in vitro. In addition, the effects of FA on AS plaques, blood lipid levels, and proliferation of the media in mice were observed in vivo, and the protective effects and possible mechanisms of FA were discussed.

## Materials and Methods

### Reagents

PDGF-BB was purchased from R&D Systems, Inc. (Minneapolis, MN, USA) and dissolved in 4 mM HCl containing 0.1% bovine serum albumin (BSA). FA (purity greater than 99%) was purchased from Sigma (St. Louis, MO, USA), dissolved in dimethyl sulfoxide (DMSO) at a concentration of 200 mM and stored at 4 ℃. The final concentration of DMSO was less than 0.1% [v/v] in all of the experiments. NO kits (catalog no. S0021) were purchased from Beyotime Biotechnology (Nanjing, China). 3-(4,5-Dimethylthiazol-2-yl)-2,5-diphenyltetrazolium bromide (MTT) was supplied by Sigma (St. Louis, MO, USA). Dulbecco’s modified Eagle’s medium (DMEM) was purchased from Life Technologies (Carlsbad, CA, USA). Primary antibodies against endothelial nitric oxide synthase (eNOS), p-eNos, p21^CIP1^, p27^Kip1^, Cyclin D1, CDK4, CyclinE, CDK2, proliferating cell nuclear antigen (PCNA), and β-actin and horseradish peroxidase-conjugated anti-rabbit antibodies were obtained from Cell Signaling Technology. PI3K, p-PI3K was purchased from abcam(UK)and AKT, p-AKT was purchased from proteintech(USA).

### Cell Culture and Cell Viability Assay

MOVAS cells (mouse aortic smooth muscle cells) and human umbilical vein endothelial cells (HUVECs) were purchased from the Cell Bank of the Chinese Academy of Sciences. The cells were cultured in DMEM supplemented with 10% heat-inactivated fetal bovine serum (FBS), penicillin (100 U/mL), and streptomycin (100 mg/mL) (all from Gibco) and maintained at 37 °C in a humidified 5% CO_2_ atmosphere.

Cell viability was assessed using the MTT assay. MOVAS cells and HUVECs (1.0×10^4^ cells/well) were seeded in 96-well plates and incubated for 24 h. Then, the cells were treated with different concentrations of FA (0, 200, 300, 400, and 1000 ng/mL) for another 24 h. Twenty microliters of MTT (5 mg/mL) working solution was added to each well, and the cells were incubated at 37 °C for 4 h. Then, the culture medium was removed, and 150 µL DMSO was added to dissolve the formazan crystals. A microplate reader (TecanM200, Switzerland) was used to measure the absorbance values at 490 nm. The assay was performed in triplicate for each concentration.

### Determination of NO Production and Western Blot Analysis

MOVAS cells and HUVECs (1.0×10^4^ cells/well) were seeded in 96-well plates and incubated overnight. Then, the culture medium was removed, and FA was added at various concentrations (0, 200, and 400 ng/mL) with or without L-NAME (L-N) for 12 h. Nitric oxide (NO) levels in the culture medium were measured directly according to the instructions of a NO assay kit (S0021, Beyotime Institute of Biotechnology, China) based on the Griess reaction. The absorbance values were measured at 540 nm using a microplate reader (TecanM200, Switzerland).

Protein expression was assessed by western blot (WB) analysis. Cells (MOVAS cells and HUVECs) pretreated with FA at different concentrations (0, 200, and 400 ng/mL) were collected, total proteins were extracted with RIPA lysis buffer (Beyotime, China), and the protein concentration was quantified using a BCA protein assay kit (Beyotime, China). Protein samples (30 µg) were separated by 10% dodecyl sulfate-polyacrylamide gel electrophoresis (SDS-PAGE) and transferred to PVDF polyvinylidene difluoride membranes (Millipore, MA, USA). Then, the membranes were incubated with the respective primary anti-body (eNOS, p-eNOS Ser1177, AKT, p-AKT Ser473 and PI3K, P-PI3K p85 alpha Y607) overnight at 4 °C. The membrane was then incubated with a corresponding horseradish peroxidase-conjugated secondary antibody (Santa Cruz, USA). Finally, the protein bands were observed with an enhanced chemiluminescence (ECL) system, and the negative bands observed after exposure were scanned and analyzed with Quantity One professional grayscale analysis software.

### Analysis of VSMC Proliferation by the MTT Assay and the Cell Cycle of VSMCs by Flow Cytometry

The effect of FA on PDGF-BB-induced proliferation of MOVAS cells was analyzed using the MTT assay. MOVAS cells were pretreated with FA (0, 200, and 400 ng/mL) with or without L-N in serum-free medium for 24 h and then stimulated with PDGF-BB (25 ng/mL) for 24 h.

The cell cycle was analyzed by flow cytometry. At the end of treatment, the cells were collected, fixed in 70% cold ethanol, and incubated at 4 °C overnight. After being washed twice with PBS, the cells were incubated with RNase A and propidium iodide (PI) solution (Sigma-Aldrich) for 30 min at 4 °C in darkness. Subsequently, the samples were analyzed with a FACSCalibur flow cytometer (version 2.0, BD Biosciences, USA) using CellQuest software. The PI signal was measured with an argon ion laser at an excitation of 488 nm through a 630-nm filter. Data for 10,000 cells was collected and plotted in a FSC/SSC scatterplot, and gating was used to eliminate adhesive cells and cell debris. The results are representative of at least three independent experiments.

### Detection of Cyclins

Cells were cultured as described above, divided into the following groups, and treated with drugs: the control (negative control) group, which was cultured without serum starvation for 24 h; the PDGF-BB (positive control) group, which was first cultured without serum for 24 h and then cultured with 10% calf serum + 25 ng/mL PDGF-BB for 24 h; the high-dose inhibitor group, which was first cultured with serum-free medium + 400 ng/mL sodium ferulate + L-N for 24 h and then with 10% calf serum + 25 ng/mL PDGF-BB for 24 h; the low-dose FA group, which was first cultured with serum-free medium + 200 ng/mL sodium ferulate for 24 h and then with 10% calf serum + 25 ng/mL PDGF-BB for 24 h; and the high-dose FA group, which was first cultured with serum-free medium + 400 ng/mL sodium ferulate for 24 h and then with 10% calf serum + 25 ng/mL PDGF-BB for 24 h.

WB analysis was performed as described previously.

### Transwell Migration Assay and Cytoskeleton Analysis

The transwell cell migration assay was performed using a modified Boyden chamber (8.0 µm pore size, Corning). The cells were trypsinized and washed with PBS, and suspended cells (1 × 10^4^ cells) were added to the upper chamber. The lower chambers were filled with the same medium (no cells) supplemented with or without PDGF-BB (25 ng/mL) in the absence or presence of FA (200 or 400 ng/mL). The cells were incubated under normal conditions for 24 h. Then, the cells that had not migrated were carefully cleared from the upper surface of the filter, while the cells on the lower surface of the filter were fixed with 4% paraformaldehyde and stained with 0.1% crystal violet. Migrated cells were counted in eight random fields under a microscope, and the average number of cells was calculated (Nikon, Tokyo, Japan).

The cytoskeleton (F-actin) of cells was analyzed by staining. Cells were washed with PBS (pH 7.4) preheated at 37 °C, fixed in 4% paraformaldehyde at room temperature for 10 min, and then washed 2 to 3 times with PBS for 10 min each. After that, the cells were permeabilized in 0.5% Triton X-100 for 5 min and incubated with fluorescein isothiocyanate-phalloidin (cell signaling). Cytoskeleton staining in the cells was analyzed using an inverted fluorescence microscope (Olympus IX73).

### Animal Experiments

#### Animals and Experimental Diet

A total of 48 six-week-old male ApoE^-/-^ mice were purchased from Peking University Laboratory Animal Center and were acclimatized for 2 weeks before the start of the experiment. Then, the mice were randomly divided into three groups: the group fed a high-fat diet (HFD) (the HFD group); the group fed a HFD and treated with simvastatin (10 mg/kg) (Hangzhou Moshadong Pharmaceutical) daily (the HFD+Simva group); and the group fed a HFD and treated with FA (120 mg/kg) daily (the HFD+FA group). Simvastatin and FA were administered by oral gavage for 16 weeks. The HFD WAS purchased from Beijing Sino Australia Feed Co., Ltd. and comprised 68.85% standard chow, 21% fat, 0.15% cholesterol, 1% sodium taurocholate, 4% milk powder, and 5% sucrose.

#### Blood Sample Collection and Analysis of Plasma Lipid Levels

After 16 weeks, the animals were euthanized after overnight fasting, and blood samples were collected using EDTA tubes. After 20 min, the plasma was separated by centrifugation at 1500 rpm for 20 min at 4 °C and stored at −20 °C until analysis. High-density lipoprotein (HDL), low-density lipoprotein (LDL), triglyceride (TG), and total cholesterol (TC) levels were measured using ELISA kits according to the manufacturer’s instructions (R&D Systems, USA).

#### Collection of Thoracic Aortas and Quantitative Analysis of Atherosclerotic Lesions

Thoracic aortas were collected, and half of each sample was fixed with 4% formaldehyde for 24 h and embedded in paraffin. The aortic root was serially sectioned into 6-μm sections and stained with hematoxylin and eosin (HE). The other half of each aorta was opened longitudinally along the ventral midline from the iliac artery to the aortic root and stained with Oil Red O. Images were captured with a Zeiss Axio camera (Carl Zeiss, Jena, Germany). Atherosclerotic lesions were quantitatively analyzed by using ImageJ software.

### Statistical Analysis

The data are expressed as the mean ± SD and were analyzed by one-way ANOVA using Minitab Statistical software version 13 (Minitab, State College, USA). In the case of a significant difference, Fisher’s exact test or the Games-Howell test was applied as a post hoc test for data homoscedastic data and heteroscedastic data, respectively. Means were considered significantly different at *p* < 0.05.

## Results

### Influence of FA on HUVECs and MOVAS Cell Activities

**Fig. 1:**
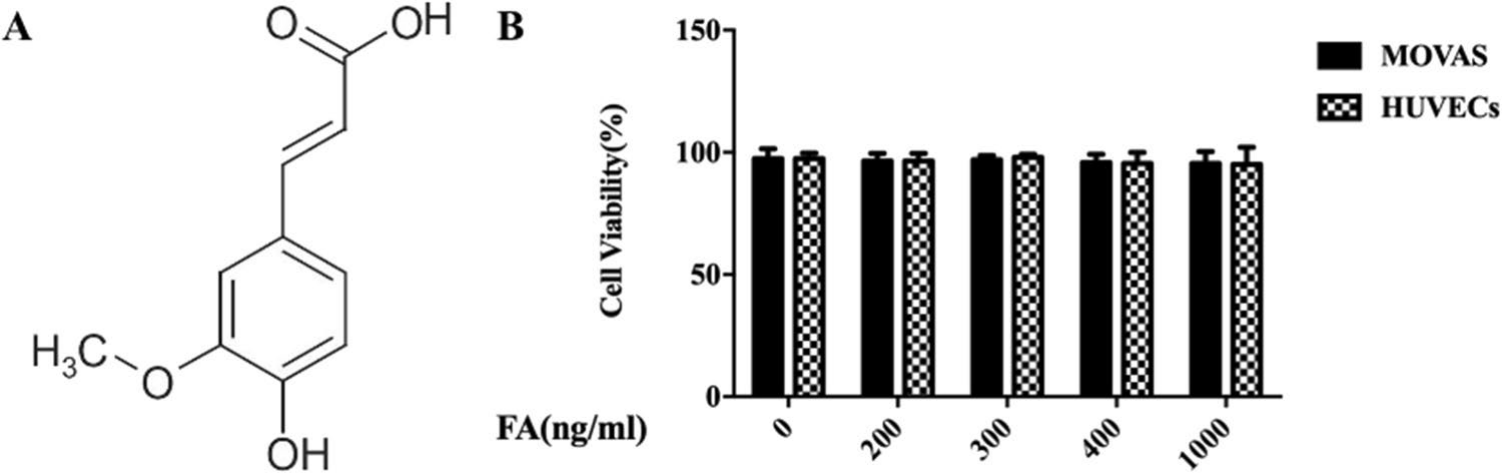
Effects of different concentrations of FA on the activities of HUVECs and MOVAS**.** FA did not show any toxicity to HUVECs and MOVAS cells

The MTT assay was used to analyze the effect of FA on HUVECs and MOVAS cell viability. According to the literature, we selected 0, 200, 300, 400, and 1000 ng/mL as the concentrations of FA. HUVECs and MOVAS cells treated with different concentrations of FA were subjected to the MTT assay after incubation for 24 h, and the results are shown in Fig. 1. FA showed no toxicity to HUVECs or MOVAS cells at a concentration of 0, 200, 300, 400, or 1000 ng/mL. Based on these results, the concentrations selected for the subsequent cell experiments were 0 ng/mL, 200 ng/mL, and 400 ng/mL.

### FA Promotes NO Generation in HUVECs and MOVAS Cells Through the PI3K/AKT/eNOS Pathway

**Fig. 2:**
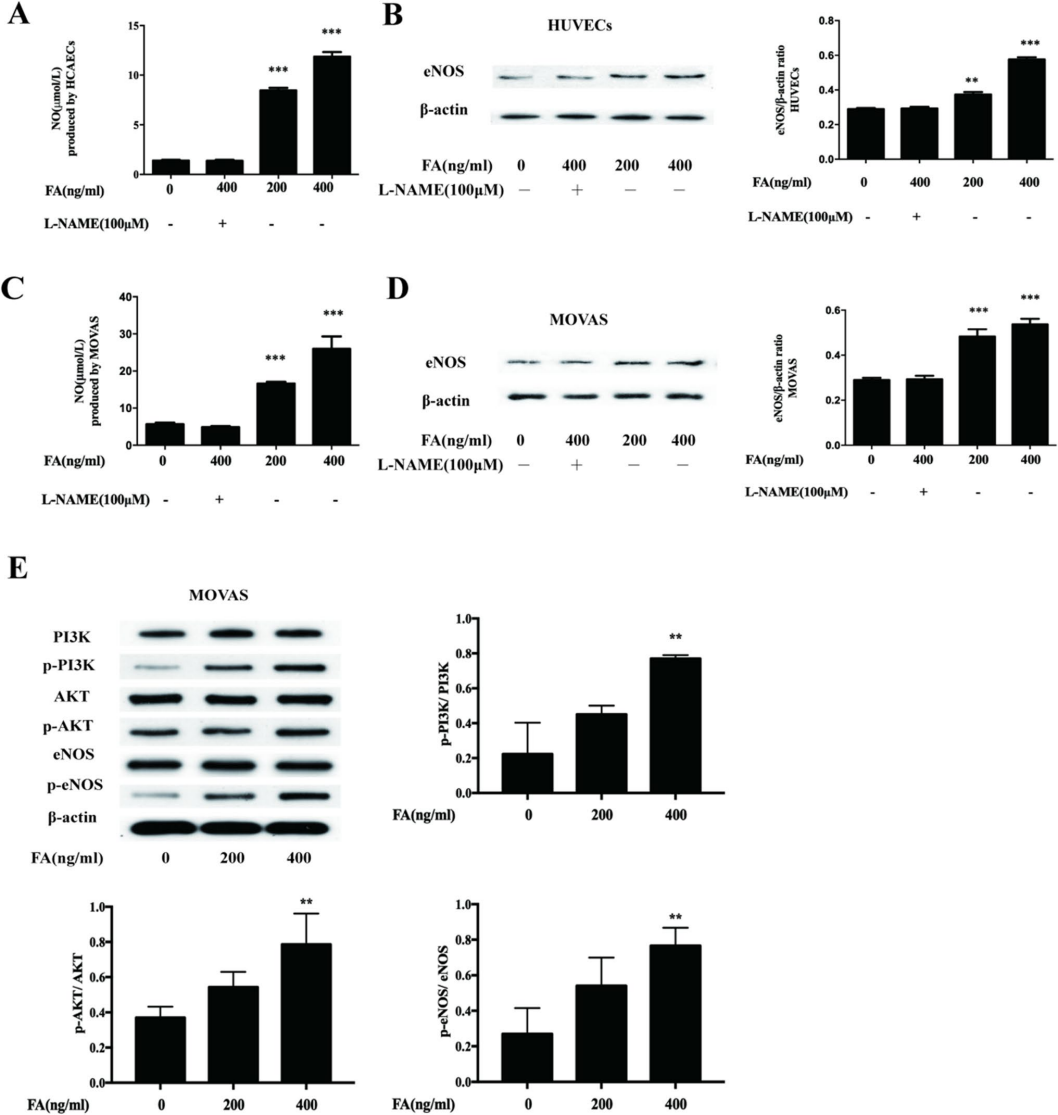
FA can promote NO generation through the PI3K/AKT/eNOS pathway. Compared with HVUEC, MOVAS control group and 400ng/ml+ L-N group, the NO production and eNOS protein expression of 200ng/ml and 400ng/ml FA were significantly increased, and the difference was statistically significant. In addition, the protein expression levels of eNOS, p-eNOS, PI3K, p-PI3K, AKT, and p-Akt were displayed after 200 ng/mL and 400 ng/mL FA treatments. The main statistical method used was Mann-Whitney U test after one-way ANOVA. ^*^p < 0.05, ^**^p < 0.01, ^***^p < 0.001 vs. control group

HUVECs and MOVAS cells were divided into four groups: the control (negative control) group; the high-dose FA plus inhibitor group, which was treated with 400 ng/mL sodium FA + L-N; the low-dose FA group, which was treated with 200 ng/mL FA; and the high-dose FA group, which was treated with 400 ng/mL FA. NO production was evaluated with an NO kit, and eNOS expression was measured by WB analysis. The results are shown in Fig. 2A–D. Compared with the control group, FA not only promoted the increased expression of eNOS in both cell lines but also significantly increased the ratios of p-PI3K/PI3K, p-AKT/AKT, and p-eNOS/eNOS after 200 ng/mL and 400 ng/mL FA treatments, However, FA had no significant effect on the expression of AKT and PI3K proteins as shown in Fig. 2E. (^*^p < 0.05, ^**^p < 0.01, ^***^p < 0.001 vs. the control group).

### FA Inhibits VSMC Proliferation and Arrests Cells in G1 Phase

**Fig. 3:**
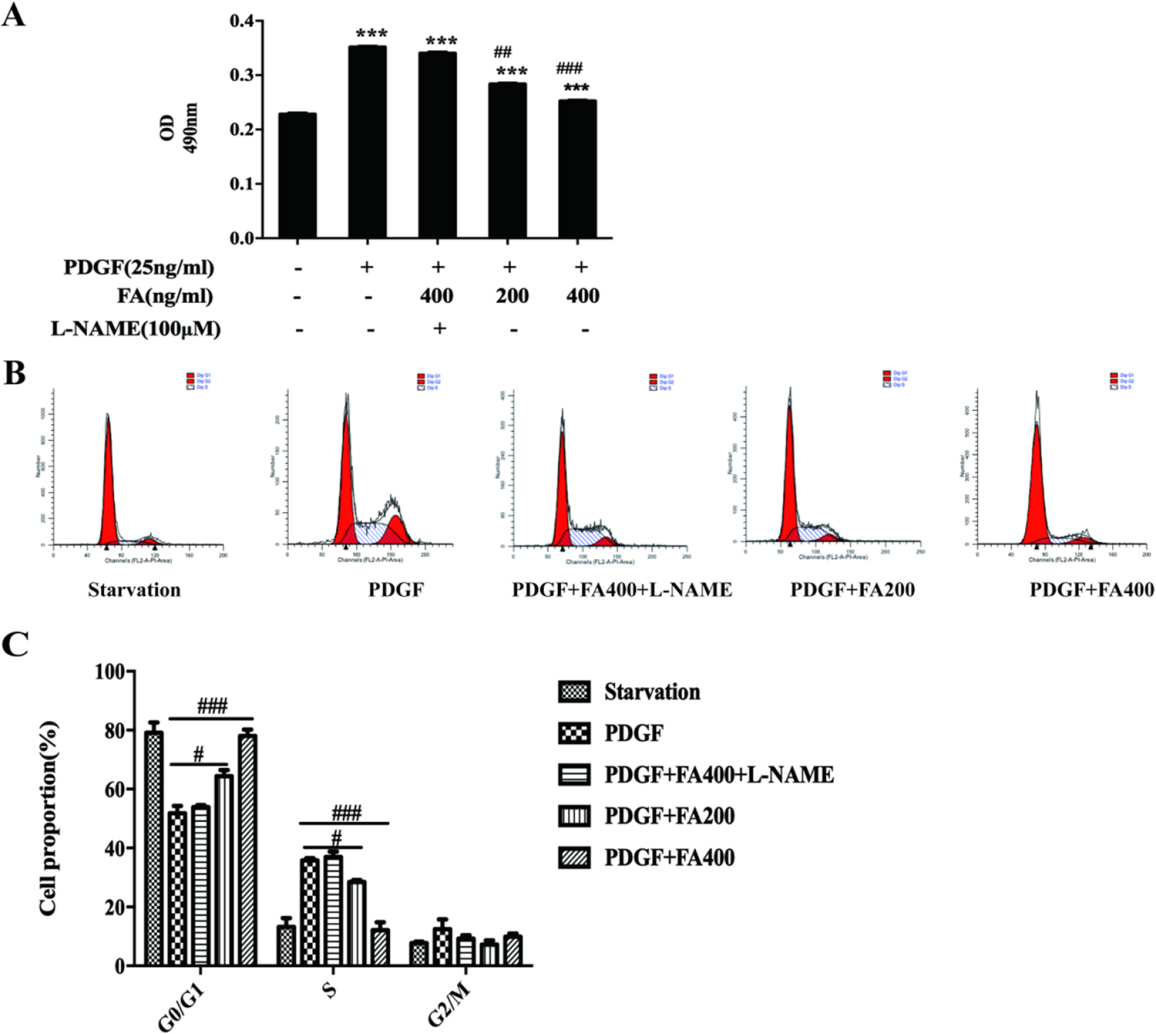
FA inhibited VSMC proliferation and stalled in G1 phase. PDGF can promote the proliferation of VSMC, while FA can inhibit the proliferation of VSMC promoted by PDGF. The main statistical method used was Mann-Whitney U test after one-way ANOVA. ^*^p < 0.05, ^**^p < 0.01, ^***^p < 0.001 vs. control group, ^#^*p* < 0.05, ^##^*p* < 0.01, ^###^*p* < 0.001 vs. PDGF group

Compared with those of the untreated control group, the OD values of the PDGF-treated group, PDGF+400 ng/mL FA+L-N-treated group, PDGF+200 mg/mL FA-treated group, PDGF+400 ng/mL FA-treated group, and PDGF+400 ng/mL FA-treated group were significantly increased. In addition, the OD values of the PDGF+200 ng/mL FA-treated group and PDGF+400 ng/mL FA-treated group were significantly decreased, and there was no significant difference in the OD value between the PDGF+400 ng/mL FA+L-N-treated group and the PDGF-treated group, as shown in Fig. 3. The results showed that PDGF promoted the proliferation of VSMCs, while FA inhibited the proliferation of VSMCs induced by PDGF (^*^p < 0.05, ^**^p < 0.01, ^***^p < 0.001 vs. the control group; ^#^*p* < 0.05, ^##^*p* < 0.01, ^###^*p* < 0.001 vs. the PDGF-treated group).

### FA Promotes p21 Expression and Corresponding Changes in Cyclin Expression in MOVAS cells

**Fig. 4:**
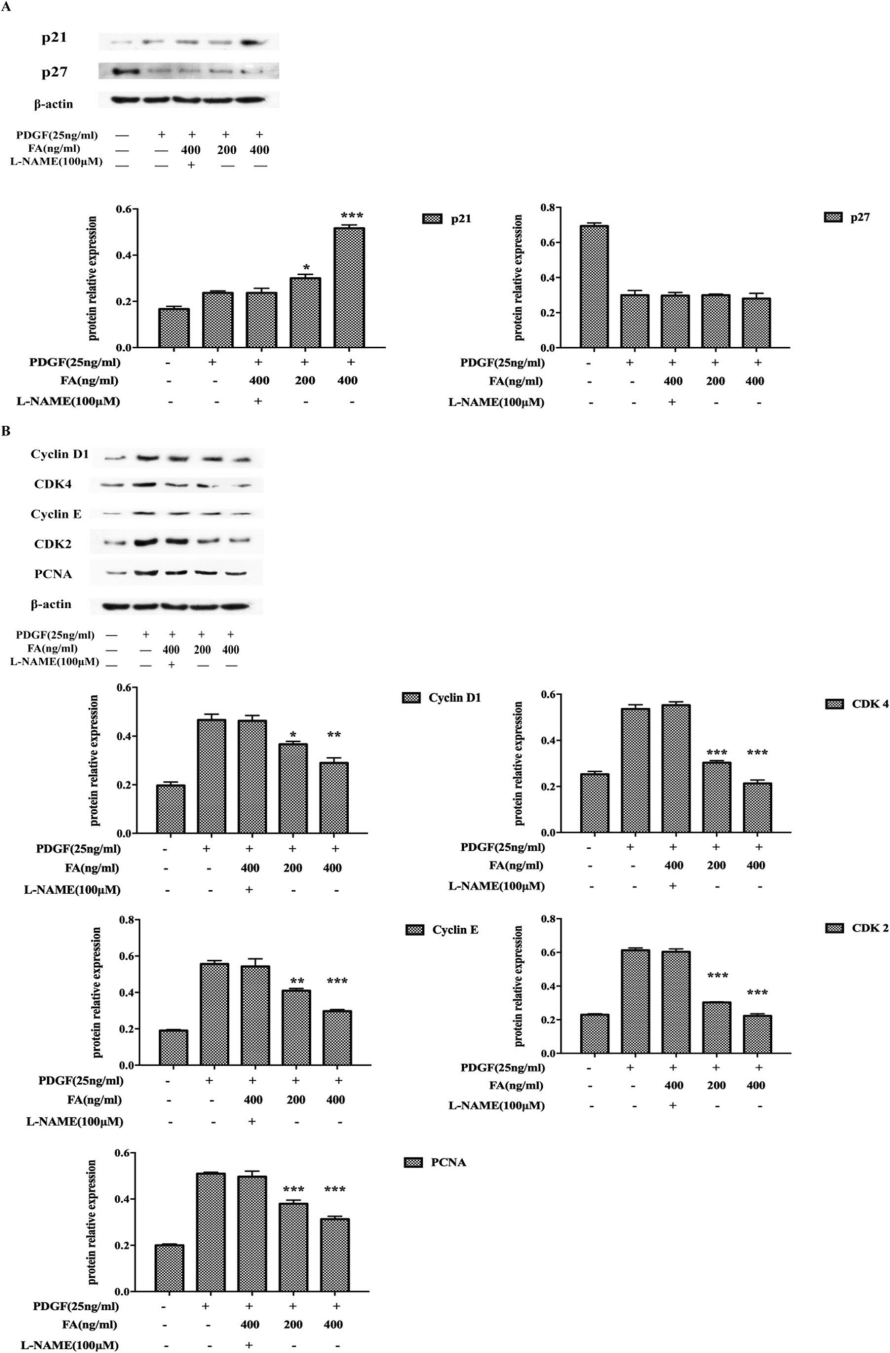
Effects of FA on P21 expression and corresponding cyclins. FA may promote the increase of p21 expression through the promotion of NO production, and the increase of p21 expression affects the subsequent cell cycle-related proteins cyclin D-CDK4, cyclin E-CDK2 complex, and PCNA expression decline. The main statistical method used was Mann-Whitney U test after one-way ANOVA. ^*^p < 0.05, ^**^p < 0.01, ^***^p < 0.001 vs. PDGF group

The results showed that FA significantly increased the expression of cyclin P21^CIP1^ in a dose-dependent manner, while the expression of cyclin D-CDK4, the cyclin E-CDK2 complex and PCNA, decreased with increasing FA concentration. However, the addition of the NO inhibitor L-N inhibited this effect, suggesting that FA may promote an increase in p21^CIP1^ expression by promoting an increase in NO production (Fig. 4) (^*^p < 0.05, ^**^p < 0.01, ^***^p < 0.001 vs. the PDGF-treated group).

### FA Inhibits VSMC Migration by Inhibiting Microfilament Aggregation

**Fig. 5:**
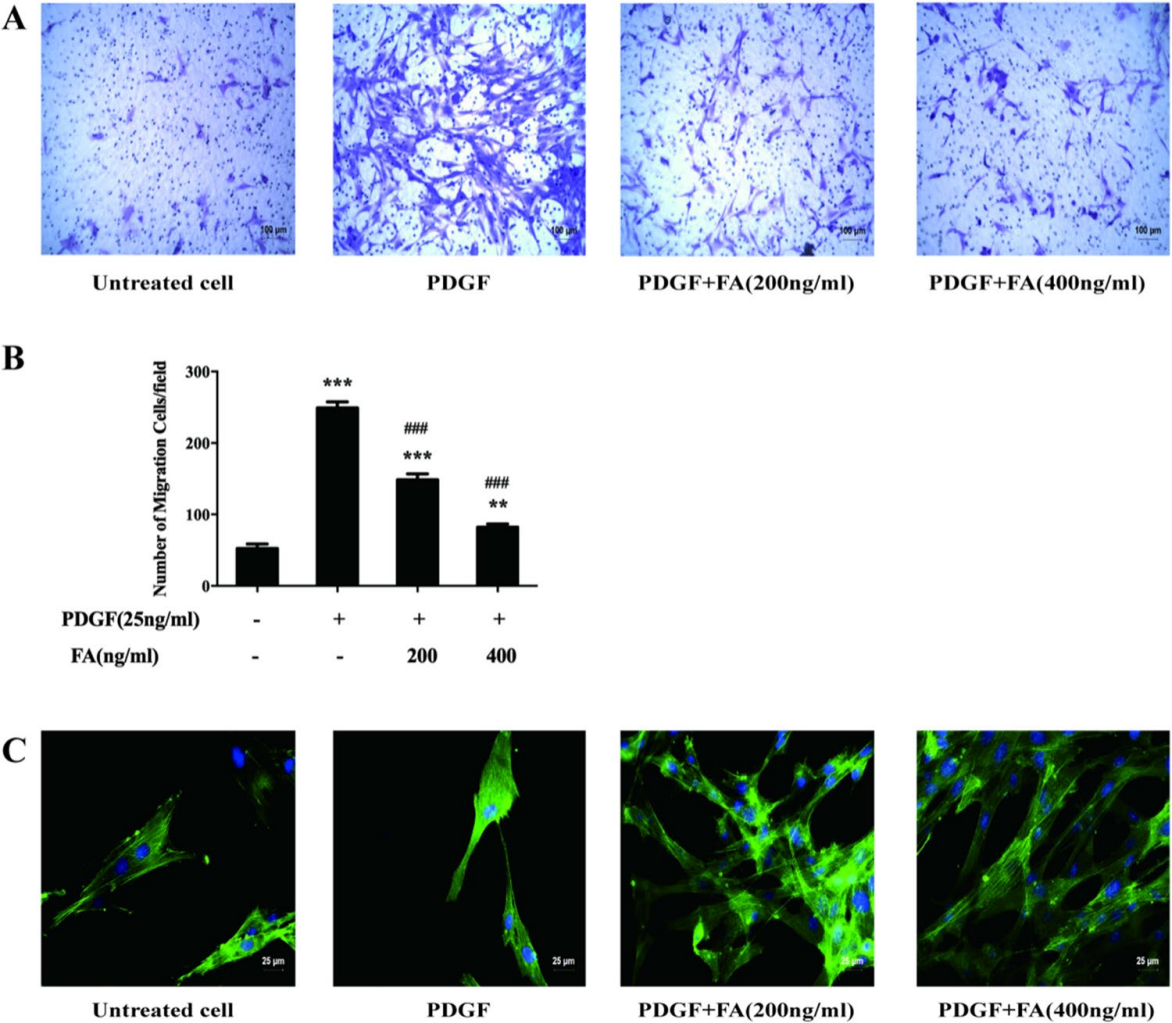
FA inhibits VSMC migration by inhibiting microfilament aggregation. FA can significantly reduce the number of cell migration, and the difference is statistically significant. After FA intervention, the amount of microfilament aggregation decreased in a dose-dependent manner. The main statistical method used was Mann-Whitney U test after one-way ANOVA. ^*^p < 0.05, ^**^p < 0.01 ^***^p < 0.001 vs. untreated cell group, ^#^*p* < 0.05, ^##^*p* < 0.01, ^###^*p* < 0.001 vs. PDGF groups

The results showed that the number of migrated cells was significantly increased in the PDGF-treated group, PDGF+200 ng/mL FA-treated group, and PDGF+400 ng/mL FA-treated group compared with the untreated control group. In addition, the number of migrated cells was significantly decreased in the PDGF+200 ng/mL FA-treated group, PDGF+400 ng/mL FA-treated group, and PDGF-treated group. The results are shown in Fig. 5A. Microfilament aggregation in the PDGF-treated group was significantly increased compared with that in the control group, and microfilament aggregation was dose-dependently reduced after FA treatment, as shown in Fig. 5B (^*^p < 0.05, ^**^p < 0.01, ***p < 0.001 vs. the control group; ^#^*p* < 0.05, ^##^*p* < 0.01, ^###^*p* < 0.001 vs. the PDGF-treated group).

### FA Is Beneficial for Alleviating AS Plaques

#### FA Reduces Blood Lipid Levels

**Fig. 6:**
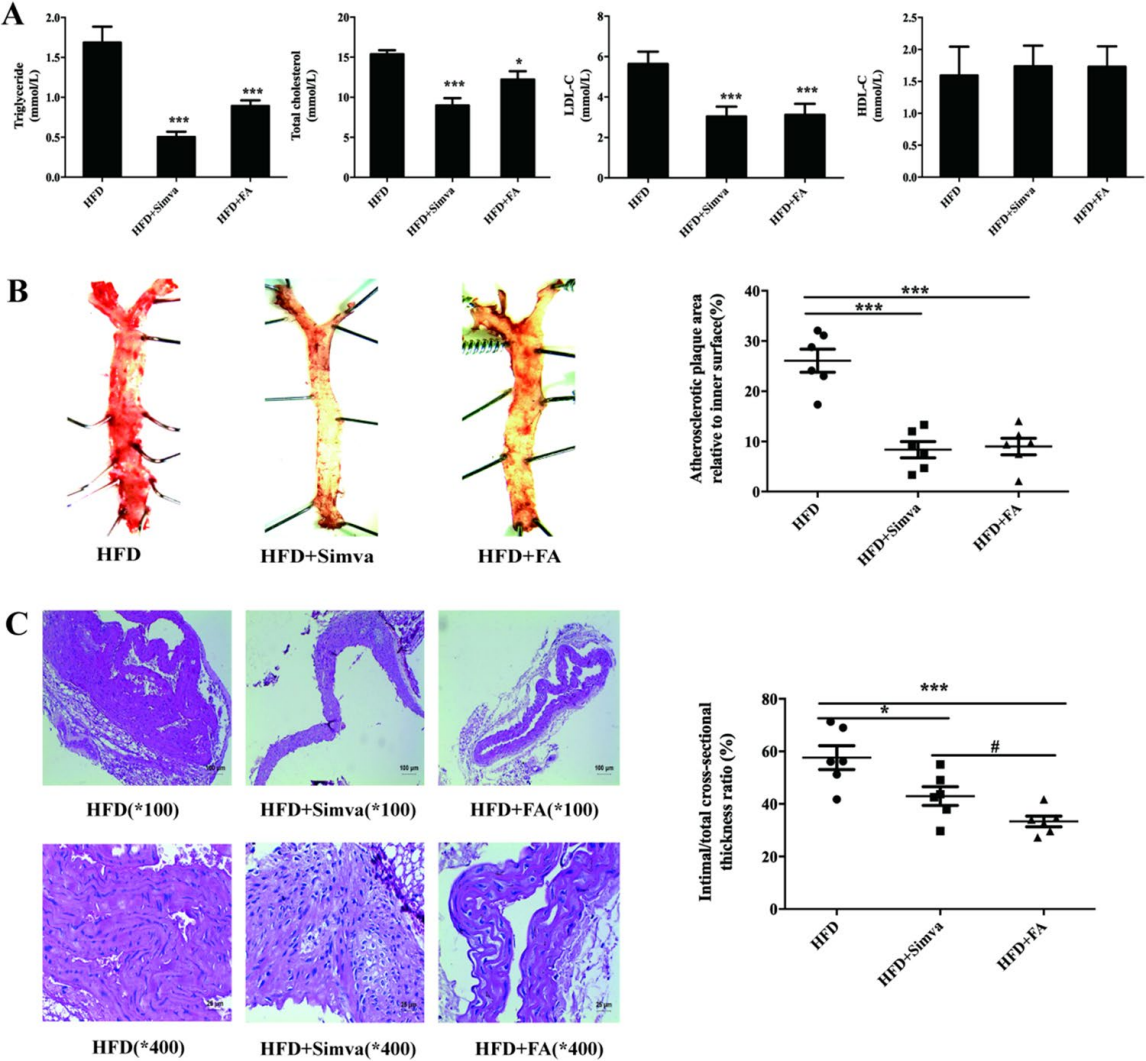
FA is beneficial for reducing AS plaques. FA could decrease the plasma TC, TG, and LDL-C levels, and the difference was statistically significant. Bolted II number, FA, simvastatin can obviously reduce the mice aorta plaque formation. Bolted II number, FA, simvastatin can significantly reduce VSMC proliferation, and differences were statistically significant. The main statistical method used was Mann-Whitney U test after one-way ANOVA. ^*^p < 0.05, ^**^p < 0.01, ^***^p < 0.001 vs. HFD group. ^#^*p* < 0.05, ^##^*p* < 0.01, ^###^*p* < 0.001 vs. HFD+Simva

The results of blood lipid analysis showed that FA and simvastatin significantly reduced the levels of TC, TG, and LDL-cholesterol (LDL-C) in the plasma of ApoE^-/-^ mice fed a HFD. Both FA and simvastatin increased HDL-cholesterol (HDL-C) content, but the difference was not statistically significant (Fig. 6A) (^*^p < 0.05, ^**^p < 0.01, ^***^p < 0.001 vs. the HFD group).

#### FA Reduces the Plaque Area

Oil red O staining showed that FA and simvastatin significantly reduced the formation of aortic plaques in mice (Fig. 6B) (^*^p < 0.05, ^**^p < 0.01, ^***^p < 0.001 vs. the HFD group).

#### FA Inhibits the Proliferation of Vascular Media

HE staining of the aortic arch showed that FA and simvastatin significantly reduced VSMC proliferation, as shown in Fig. 6C (^*^p < 0.05, ^**^p < 0.01, ^***^p < 0.001 vs. the HFD group; ^#^*p* < 0.05, ^##^*p* < 0.01, ^###^*p* < 0.001 vs. the HFD + Simva group).

## Discussion

AS is a disease that seriously endangers human health. Excessive proliferation and migration of VSMCs play an important role in the occurrence and development of AS. FA has a variety of promising antiatherosclerotic effects, but little research has been done on VSMCs. The in vitro experiments in this study revealed that FA inhibited the migration and proliferation of VSMCs induced by PDGF and arrested cells in G0/G1 phase. Further studies showed that FA promoted the production of NO in VSMCs to increase the expression of p21 in these cells, affected the levels of related cyclins, ultimately led to cell cycle arrest, and played a role in inhibiting cell proliferation. In the animal experiment, ApoE^-/-^ mice were fed a HFD to establish the animal model of AS, and simvastatin and FA were given. The results showed that compared with no treatment, FA and simvastatin significantly reduced the levels of blood lipids and reduced the area of atherosclerotic plaques in mice.

FA is abundant in vegetables, fruits, and some Chinese herbal medicines [3]. Currently, the effect of FA on the proliferation and migration of VSMCs is not clear. Studies have shown that FA can inhibit the overproliferation of VSMCs induced by angiotensin 2, but the mechanism is not clear [10]. In another study, FA was shown to promote the proliferation of MCF7 cells, BT20 human breast cancer cells, and neural progenitor cells in vivo and in vitro, suggesting that FA may exhibit different biological activities in different cell types [11]. On the basis of a literature review, we selected 0, 200, 300, 400, and 1000 ng/mL and the concentrations of FA and conducted the MTT assay [12]. The results showed that FA did not show any toxicity to HUVECs or MOVAS cells at 0, 200, 300, 400, or 1000 ng/mL. Based on these results, subsequent cell experiments were carried out at concentrations of 0, 200 ng/mL, and 400 ng/mL. We observed the effects of PDGF-BB on the proliferation and migration of VSMCs by the MTT assay, flow cytometry, and migration experiments. The results showed that PDGF-BB significantly promoted the proliferation and migration of VSMCs and promoted arrest in the G1 phase.

Studies have shown that some vasodilators that induce the release of NO, such as sodium nitroprusside and nitroglycerin, can inhibit the proliferation of VSMCs in vitro and that the precursor of NO (L-arginine) can prevent intimal thickening after balloon dilation, suggesting that NO can inhibit the proliferation of VSMCs in vivo [13]. NO is a small, diffusive, lipophilic free radical. As an important signaling molecule in the body, it has important and diverse signaling functions in almost every organ system in the body [14]. Nitric oxide synthase (NOS) catalyzes the production of NO. There are three isozymes of NOS in the human body, namely, neural nitric oxide synthase (nNOS), eNOS, and inducible nitric oxide synthase (iNOS). Among them, eNOS is the main source of NO in the cardiovascular system and plays an important role in cardiovascular diseases [15]. eNOS is not only expressed in vascular endothelial cells but also in cardiomyocytes, platelets, smooth muscle cells, bone cells, and neuron cells [16]. Regulation of eNOS includes regulation of transcription level and post-translational level. For example, eNOS mRNA synthesis can be improved by reducing eNOS mRNA degradation and inhibiting HO-GTPase, and eNOS activity can be improved by phosphorylation of Serl 177 through AKT pathway. Many drugs can increase the expression and activity of eNOS through different pathways, promote the multiple coupling of eNOS, and then induce the production of NO [17]. PI3K/AKT/eNOS is a classic signaling pathway widely existing in cells, which plays an important role in many aspects of vivo through phosphorylation or dephosphorylation, especially in cardiovascular diseases such as AS [18, 19]. Previous experiments by our group suggested that ferulic acid promoted NO output in endothelial cells through the PI3K/AKT/eNOS pathway [20]. In this research, we proved that FA can not only promote the increased expression of eNOS in the two cell lines but also promote the phosphorylation of eNOS in endothelial cells and vascular smooth muscle cells through the PI3K/AKT/eNOS signaling pathway, thus increasing the activity of eNOS and ultimately promoting the production of NO. However, FA has NO significant effect on the expression of AKT and PI3K proteins.

Therefore, we asked whether FA inhibits PDGF-BB-induced VSMC proliferation, as FA can promote the production of NO in VSMCs or endothelial cells; thus, we chose to use the inhibitor L-N [21]. Our study shows that FA can promote the production of NO by VSMCs and endothelial cells, while the inhibitor L-N significantly reduces the production of NO. In addition, our results showed that the inhibition of VSMC proliferation by FA is indeed related to NO.

The proliferation of VSMCs is regulated by the cell cycle, which consists of three distinct sequential phases (G0/G1, S, and G2/M) [22]. Each cell cycle checkpoint is tightly regulated. Thus, specific cell cycle checkpoints are considered the “last common pathway” for regulating proliferation [23]. Cyclin-dependent kinases (CDKs) and cyclin-dependent kinase inhibitors (CKIs) play an important role in the regulation of the cell cycle in eukaryotes and provide energy for cell cycle transition [24]. For example, cyclin D-CDK4 and cyclin E-CDK2 complexes cooperate with PCNA to regulate G1-S transformation. Subsequent G2-M transition is regulated by cyclin A-CDK 2 and cyclin B-CDK 1 complexes. CDK inhibitors such as p27^Kip1^ and p21^CIP1^ bind to CDKs and block the kinase activity of these cyclin-CDK complexes, leading to G1 phase arrest [25, 26]. Analysis of cell cycle-related proteins showed that FA promoted the expression of p21^CIP1^ by promoting the production of NO followed by the expression of cyclin D-CDK4, the cyclin E-CDK2 complex, and PCNA but did not alter the expression of p27^Kip1^. Therefore, FA may inhibit the proliferation of VSMCs through the NO/p21 signaling pathway.

We also conducted animal experiments to observe the therapeutic effect of FA on AS animal models. The ApoE^-/-^ mouse model of AS induced by HFD feeding is a relatively well-recognized model. We randomly divided ApoE^-/-^ mice into three groups. After 12 weeks of FA treatment, blood samples were collected from each group, and the levels of blood lipids were measured. Thoracic aortas were stained with Oil Red O to observe the formation of plaques, and aortic arch sections were stained with HE to observe the proliferation of intima and smooth muscle in the vascular media. The results showed that FA, like simvastatin, reduced the levels of blood lipids in ApoE^-/-^ mice, reduced plaque formation, and inhibited the proliferation of VSMCs.

This study showed that FA inhibited the excessive proliferation of VSMCs induced by PDGF in vitro and arrested VSMCs in G1 phase. Further experiments showed that FA inhibited VSMC migration and proliferation by promoting NO production in VSMCs. The expression of cyclin-related proteins was measured, and it was found that the expression of p21^CIP1^ was increased, while the expression of p27^Kip1^ was not significantly changed. The expression of cyclin D-CDK4, the cyclin E-CDK2 complex, and PCNA was decreased. When the inhibitor L-N was added, these effects were inhibited. It was concluded that FA inhibits VSMC proliferation through the NO/p21 signaling pathway. An AS animal model was successfully established by feeding ApoE^-/-^ mice a HFD, and the data showed that FA had the same effect as simvastatin in reducing the degree of vascular lesions and inhibiting the proliferation of VSMCs in the intima and medial vessels. It can be inferred that FA can not only reduce blood lipid levels but also treat AS by inhibiting the migration and proliferation of VSMCs. Therefore, the results of this study confirm that early inhibition of VSMC migration and proliferation is beneficial for the treatment of AS.

## Conclusion

This study showed that FA mainly inhibits the excessive proliferation of VSMCs through the NO/P21 pathway and plays an antiatherosclerotic role. Studies have also shown that the use of FA to inhibit the excessive proliferation of VSMCs in the early stage is beneficial for the treatment of AS, but the advantages and disadvantages of stabilizing atherosclerotic plaques in the later stage of the disease need to be further studied. This study also suggests that FA is a potential drug for the treatment of vascular proliferative diseases.

## Supplementary Information

Below is the link to the electronic supplementary material.

Supplementary file1 (DOCX 123 KB)

Supplementary file2 (DOCX 1588 KB)

Supplementary file3 (DOCX 661 KB)

Supplementary file4 (DOCX 2204 KB)

Supplementary file5 (DOCX 4353 KB)

Supplementary file6 (DOCX 2895 KB)

Supplementary file7 (DOCX 880 KB)

## Data Availability

The raw data are available.

## Code Availability

SPSS statistical software output.

## References

[1] T. Sahranavard, F. Carbone, F. Montecucco, S. Xu, K. Al‐Rasadi, T. Jamialahmadi, A.J.E.J.o.C.I. Sahebkar, the role of potassium in atherosclerosis, (2020). 10.1111/eci.13454 33216974

[2] F. Agne, P. Maria, E. Paul, T.J.J.C.S 2018 Resink smooth muscle cell-driven vascular diseases and molecular mechanisms of VSMC plasticity, 52 48-64. 10.1016/j.cellsig.2018.08.019 30172025

[3] H.S. Tuli, A. Chaudhary, V.S. Jaswal, S. Choudhary, S.J.R.P.o.I. Sharma, A.D 2019 Discovery, ferulic acid: A promising therapeutic phytochemical and recent patents advances, 13 10.2174/1872213X13666190621125048 31223096

[4] Zdunska, Kamila, Dana, Agnieszka, Kolodziejczak, Anna, Rotsztejn, H.J.S 2018 Pharmacology, physiology, antioxidant properties of ferulic acid and its possible application 10.1159/000491755 30235459

[5] S. Thapliyal, T. Singh, S. Handu, M. Bisht, P. Kumari, P. Arya, P. Srivastava, R.J.N.r 2021 Gandham, a review on potential footprints of ferulic acid for treatment of neurological disorders 10.1007/s11064-021-03257-6 33547615

[6] Z. Luo, T. Liao, Y. Zhang, H. Zheng, Q. Sun, F. Han, Z. Yang, Q.J.F.i.i 2020 Sun, triptolide attenuates transplant vasculopathy through multiple pathways, 11 612. 10.3389/fimmu.2020.00612 PMC7186401 32373115

[7] H.H. Yang, Y.X. Xu, J.Y. Chen, H.J. Harn, T.W.J.I.J.o.M.S. Chiou, n-butylidenephthalide inhibits the phenotypic switch of VSMCs through activation of AMPK and prevents stenosis in an arteriovenous fistula rat model, 21 (2020). 10.3390/ijms21197403 PMC7582375 33036484

[8] S. Pi, L. Mao, J. Chen, H. Shi, B.J.A. Hu, the P2RY12 receptor promotes VSMC-derived foam cell formation by inhibiting autophagy in advanced atherosclerosis, (2020). 10.1080/15548627.2020.1741202 PMC8078663 32160082

[9] W. An, L.A. Luong, N.P. Bowden, M. Yang, Q.J.C.R. Xiao, cezanne is a critical regulator of pathological arterial remodelling by targeting β-catenin signalling, (2021). 10.1093/cvr/cvab056 PMC8803089 33599243

[10] Y.Z. Hou, J. Yang, G.R. Zhao, Y.J.J.E.J.o.P. Yuan, ferulic acid inhibits vascular smooth muscle cell proliferation induced by angiotensin II, 499 (2004) 85-90. 10.1016/j.ejphar.2004.07.107 15363954

[11] H. Elansary, A. Szopa, P. Kubica, F. A. Al-Mana, E. Mahmoud, T. Zin El-Abedin, M. A. Mattar, H.J.M. Ekiert, phenolic compounds of Catalpa speciosa, Taxus cuspidate, and Magnolia acuminata have antioxidant and anticancer activity, 24 (2019). 10.3390/molecules24030412 PMC6384650 30678123

[12] R. Nankar, P.K. Prabhakar, M.J.P.I.J.o.P. Doble, phytopharmacology, hybrid drug combination: Combination of ferulic acid and metformin as anti-diabetic therapy, (2017) S0944711317301551. 10.1016/j.phymed.2017.10.015 29126698

[13] O.S. Ekhtear Hossain, Yuan Li, Madhu B. Anand-Srivastava %J Canadian Journal of Physiology, Pharmacology, sodium nitroprusside attenuates hyperproliferation of vascular smooth muscle cells from spontaneously hypertensive rats through the inhibition of overexpression of AT1 receptor, cell cycle proteins, and c-Src/growth factor receptor signaling pathways, 98 (2020). 10.1139/cjpp-2019-0338 31577906

[14] Yang Y, Huang Z, Li LJN (2021). Advanced nitric oxide donors: Chemical structure of NO drugs. NO nanomedicines and biomedical applications.

[15] C. Meza, J. La Favor, D. Kim, R.J.I.j.o.m.s. Hickner, endothelial dysfunction: Is there a hyperglycemia-induced imbalance of NOX and NOS?, 20 (2019). 10.3390/ijms20153775 PMC6696313 31382355

[16] S. Huerta, S. Chilka, B.J.I.j.o.o. Bonavida, nitric oxide donors: Novel cancer therapeutics (review), 33 (2008) 909-927. 18949354

[17] D. Dudzinski, T.J.C.r. Michel, Life history of eNOS: Partners and pathways, 75 (2007) 247-260. 10.1016/j.cardiores.2007.03.023 PMC2682334 17466957

[18] D. Dudzinski, J. Igarashi, D. Greif, T.J.A.r.o.p. Michel, toxicology, the regulation and pharmacology of endothelial nitric oxide synthase, 46 (2006) 235-276. 10.1146/annurev.pharmtox.44.101802.121844 16402905

[19] L. Chen, R. Ackerman, A.J.P. Guo, o.l. mediators, 20-HETE in neovascularization, 98 (2012) 63-68. 10.1016/j.prostaglandins.2011.12.005 22227460

[20] Y. Huang, M. Xu, J. Li, K. Chen, L. Xia, W. Wang, P. Ren, X.J.F.c. Huang, ex vivo to in vivo extrapolation of syringic acid and ferulic acid as grape juice proxies for endothelium-dependent vasodilation: Redefining vasoprotective resveratrol of the French paradox, 363 (2021) 130323. 10.1016/j.foodchem.2021.130323 34247035

[21] Yang, Gao, Ping, Zhu, Shang-Fu, Yi-Qi, Jiang, Deng, D.-L.J. Biomedicine, p. biomedecine, pharmacotherapie, ginsenoside Re inhibits PDGF-BB-induced VSMC proliferation via the eNOS/NO/cGMP pathway, 115 (2019) 108934-108934. 10.1016/j.biopha.2019.108934 31082773

[22] S. Guo, R. Zhang, Q. Liu, Q. Wan, Y. Wang, Y. Yu, G. Liu, Y. Shen, Y. Yu, J.J.F.j.o.p.o.t.F.o.A.S.f.E.B. Zhang, 2,3,7,8-Tetrachlorodibenzo-p-dioxin promotes injury-induced vascular neointima formation in mice, 33 (2019) 10207-10217. 10.1096/fj.201900546R 31216422

[23] L. Elia, P. Kunderfranco, P. Carullo, M. Vacchiano, F. Farina, I. Hall, S. Mantero, C. Panico, R. Papait, G. Condorelli, M.J.T.J.o.c.i. Quintavalle, UHRF1 epigenetically orchestrates smooth muscle cell plasticity in arterial disease, 128 (2018) 2473-2486. 10.1172/JCI96121 PMC5983314 29558369

[24] M. Galbraith, H. Bender, J.J.T. Espinosa, therapeutic targeting of transcriptional cyclin-dependent kinases, 10 (2019) 118-136. 10.1080/21541264.2018.1539615 PMC6602565 30409083

[25] O.P. Mathew, K. Ranganna, J. Mathew, M. Zhu, Z. Yousefipour, C. Selvam, S.G.J.I.J.o.M.S. Milton, cellular effects of butyrate on vascular smooth muscle cells are mediated through disparate actions on dual targets, histone deacetylase (HDAC) activity and PI3K/Akt signaling network, 20 (2019). 10.3390/ijms20122902 PMC6628026 31197106

[26] K.J.C.D. Engeland, differentiation, cell cycle arrest through indirect transcriptional repression by p53: I have a DREAM, 25 (2017). 10.1038/cdd.2017.172 PMC5729532 29125603

