# Supplementary figures and images for: Ferulic Acid Alleviates Atherosclerotic Plaques by Inhibiting VSMC Proliferation Through the NO/p21 Signaling pathway
Source: J Cardiovasc Transl Res. 2022 Jan 6;15(4):865–75. doi: 10.1007/s12265-021-10196-8 (PMC9622559; doi:10.1007/s12265-021-10196-8)

**Figure 1A**


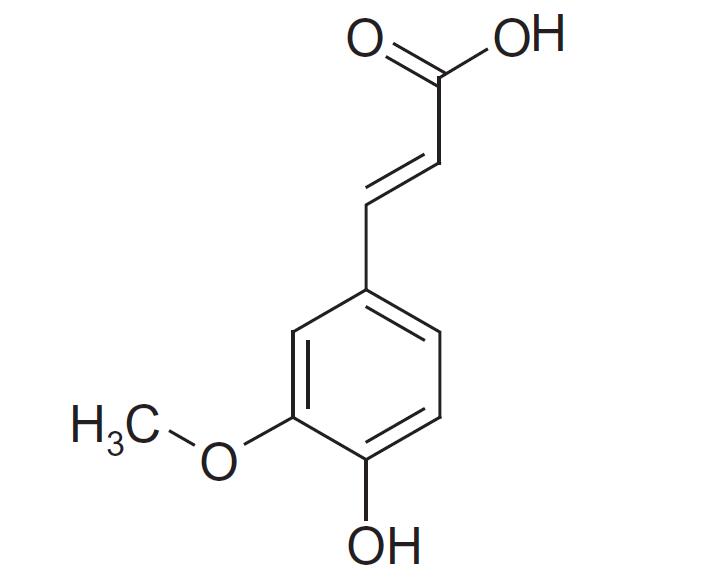


**Figure 1B**

|  | MOVAS | | | HUVECs | | |
| --- | --- | --- | --- | --- | --- | --- |
| 0 | 99.49 | 99.93 | 92.89 | 99.37 | 94.99 | 98.01 |
| 200 | 93.06 | 97.33 | 99.01 | 92.76 | 97.83 | 98.55 |
| 300 | 97.88 | 98 | 95.02 | 96.53 | 98.2 | 99.01 |
| 400 | 97.04 | 92 | 98.43 | 90.26 | 98.66 | 97.3 |
| 1000 | 99 | 97.45 | 90 | 99.42 | 87 | 98.78 |


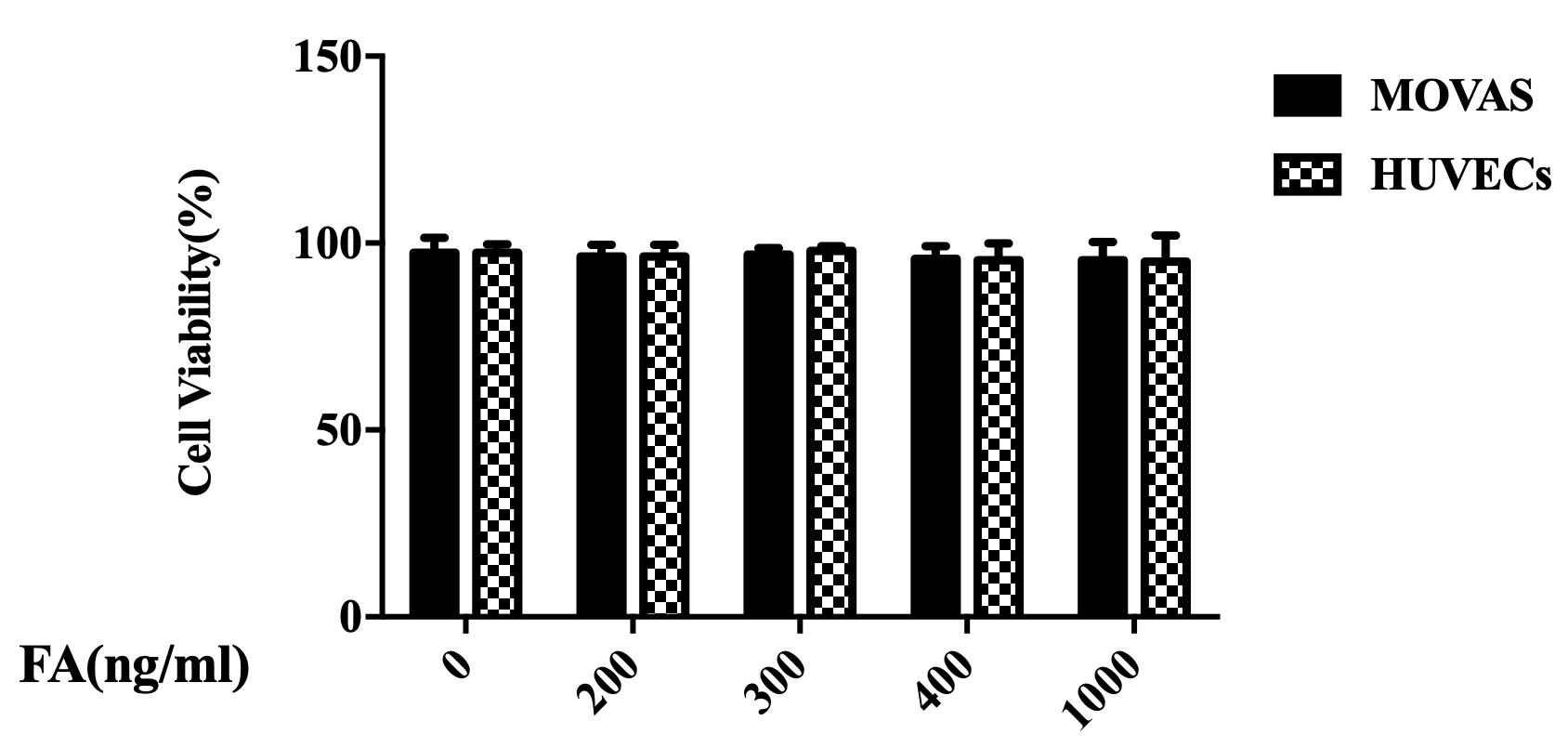

Supplement: Supplementary file 1 — Supplementary file1 (DOCX 123 KB) [file 12265_2021_10196_MOESM1_ESM.docx]

**Figure 2**

**
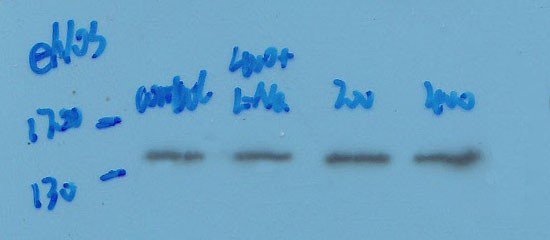
**

**
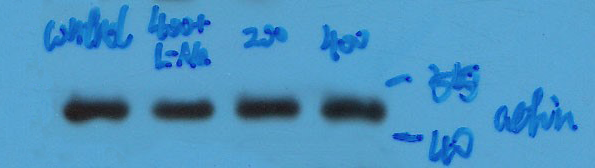
**

**
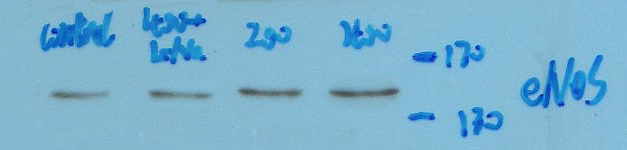
**

**
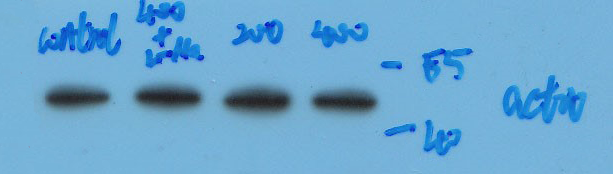
**

| 1.4575 | 1.3964 | 9.132 | 11.8871 |
| --- | --- | --- | --- |
| 1.5812 | 1.4567 | 7.455 | 12.1134 |
| 1.5459 | 1.5321 | 8.219 | 12.879 |
| 1.4013 | 1.6101 | 9.019 | 10.087 |
| 1.3213 | 0.9214 | 8.213 | 13.145 |
| 1.0214 | 1.3012 | 8.758 | 11.012 |


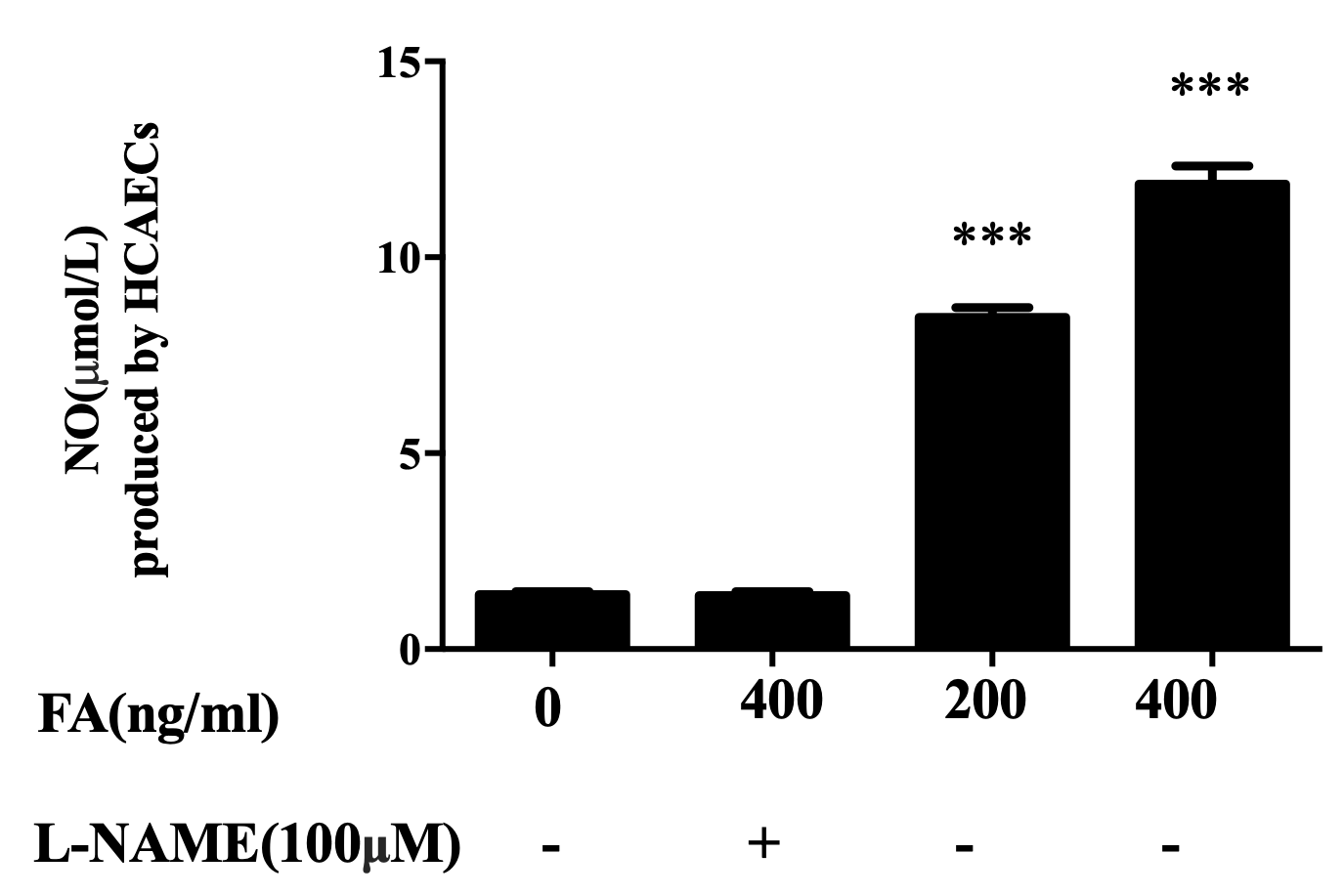


| 5.67 | 5.1 | 15.64 | 27.47 |
| --- | --- | --- | --- |
| 5.93 | 3.9 | 16.07 | 30.57 |
| 6.34 | 5.79 | 17.78 | 10.01 |
| 7.03 | 4.21 | 18.02 | 33.28 |
| 4.92 | 4.79 | 16.78 | 28.46 |
| 4.23 | 5.45 | 15.44 | 26.01 |


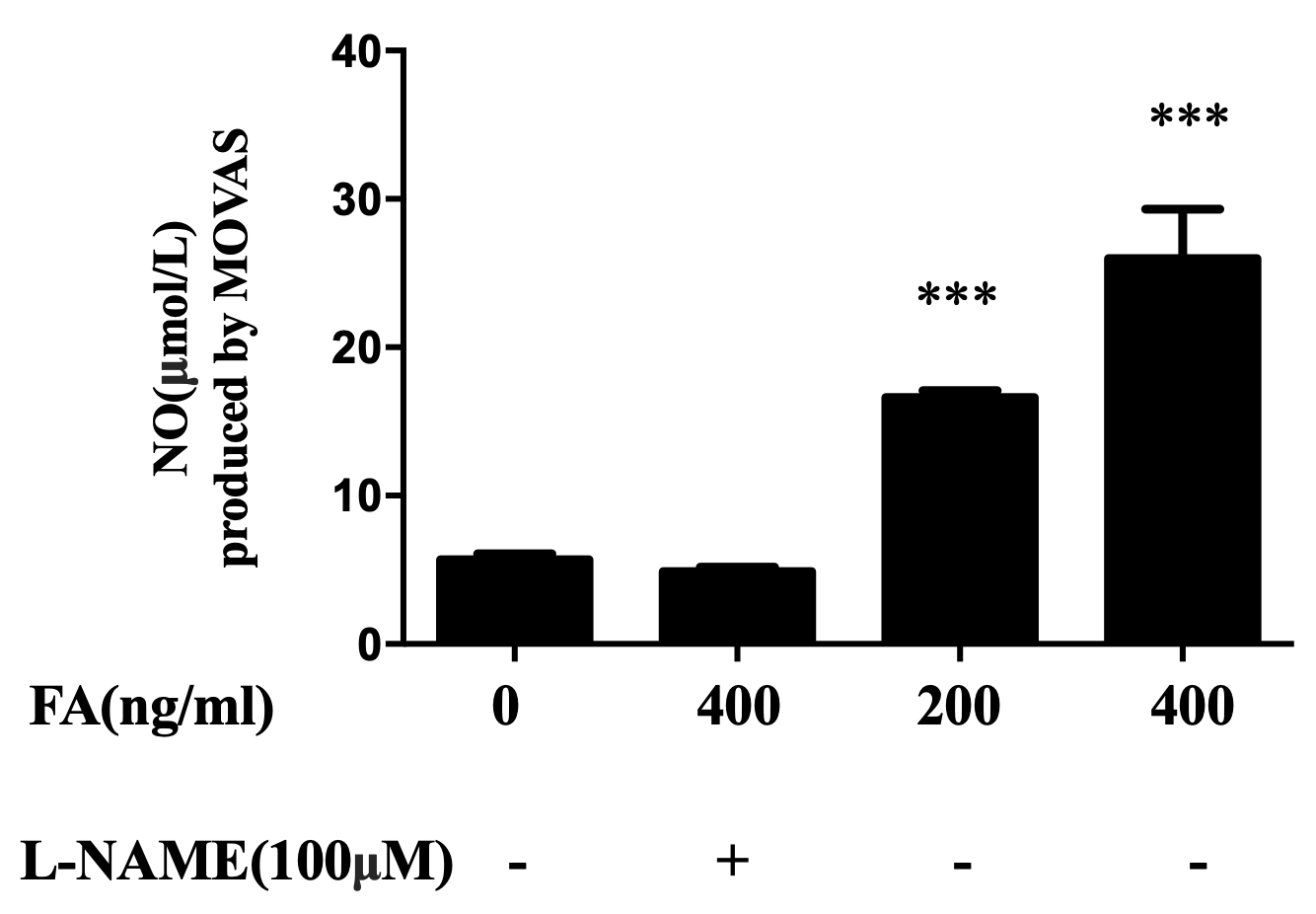


| 0.29 | 0.28 | 0.37 | 0.57 |
| --- | --- | --- | --- |
| 0.3 | 0.31 | 0.4 | 0.6 |
| 0.28 | 0.29 | 0.35 | 0.56 |


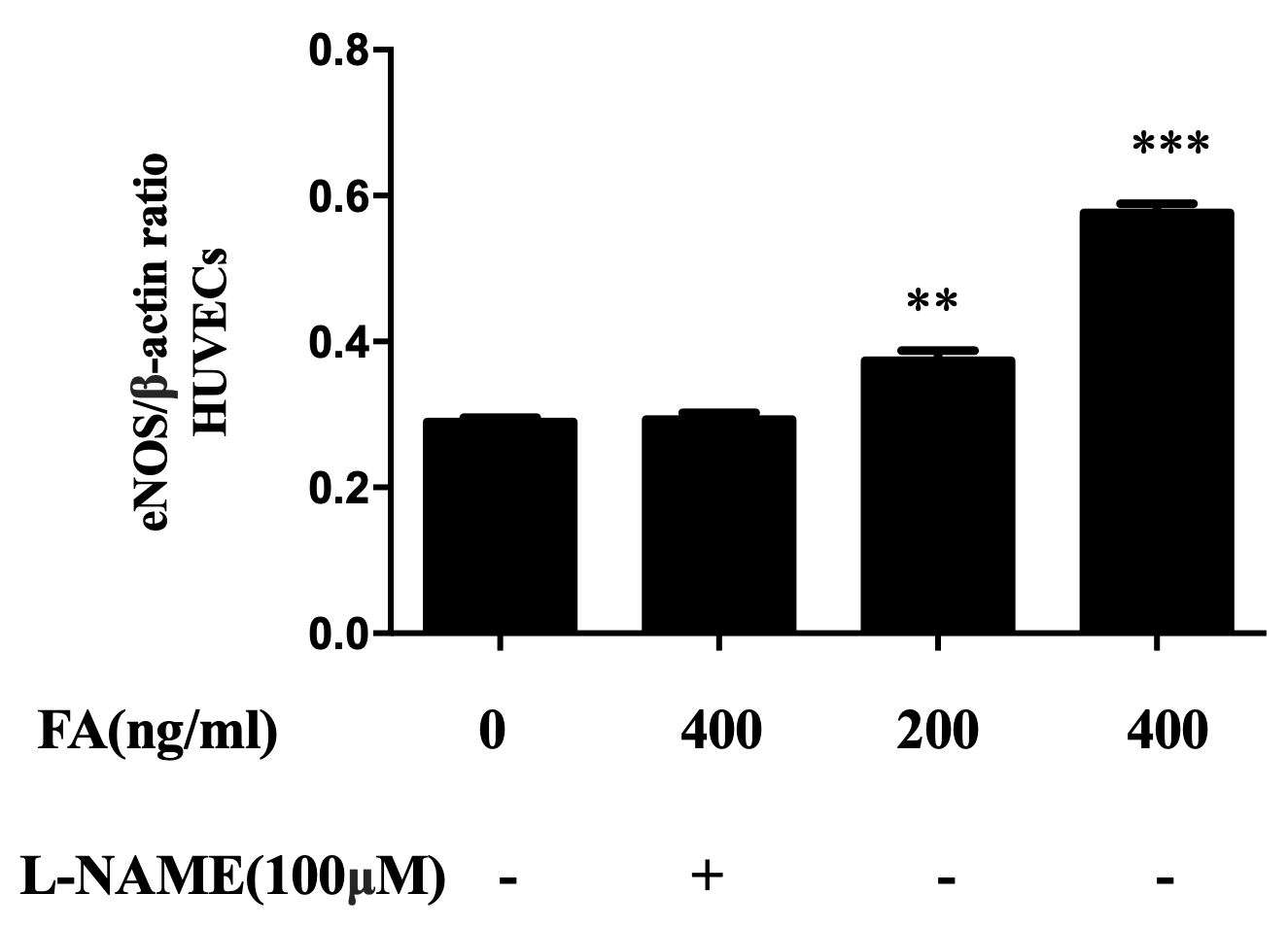


| 0.29 | 0.31 | 0.47 | 0.51 |
| --- | --- | --- | --- |
| 0.3 | 0.28 | 0.52 | 0.56 |
| 0.28 | 0.29 | 0.46 | 0.54 |


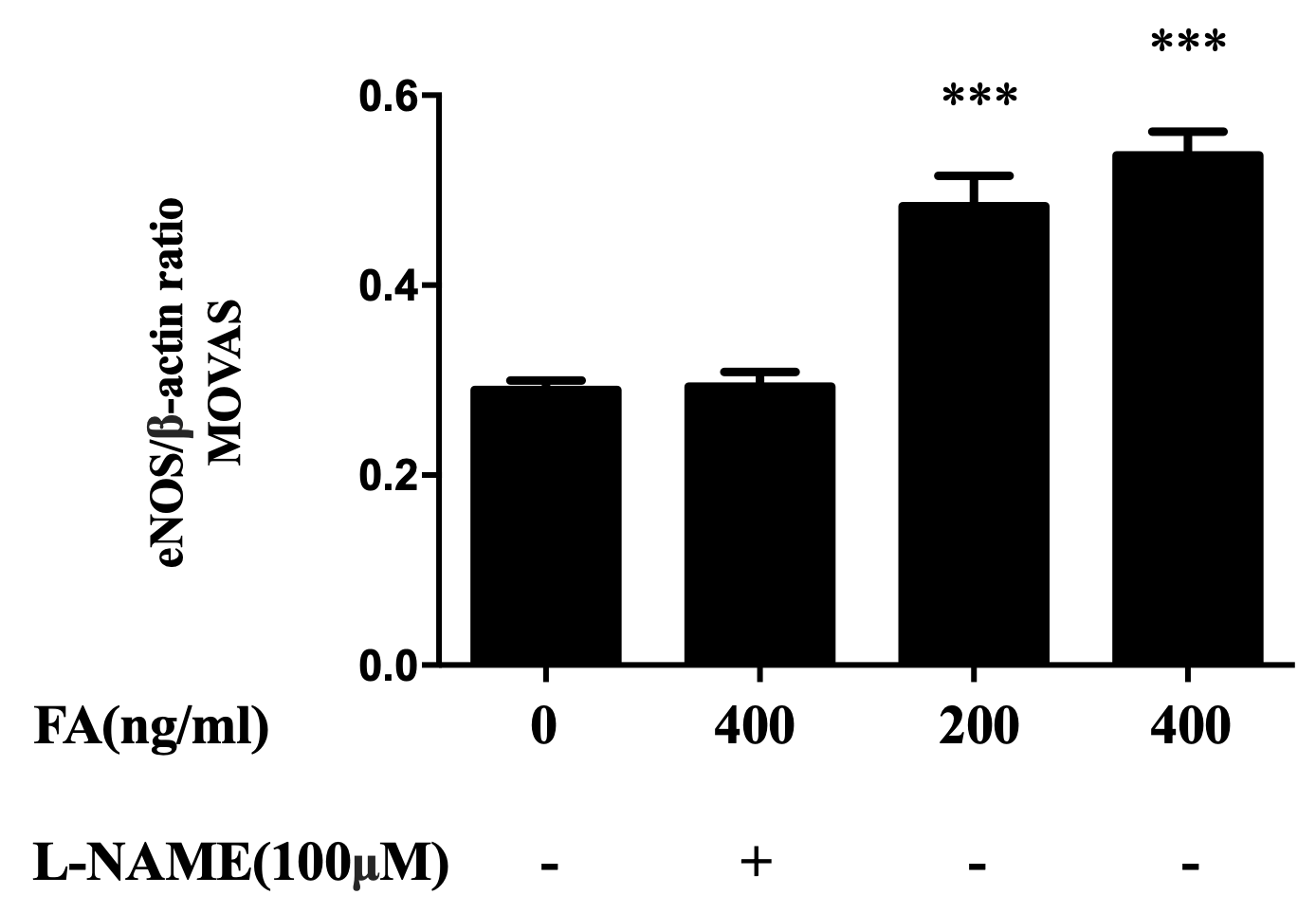


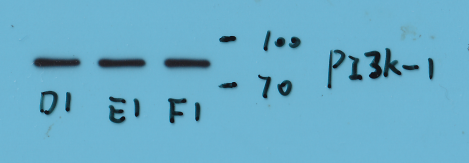


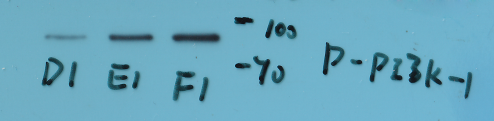


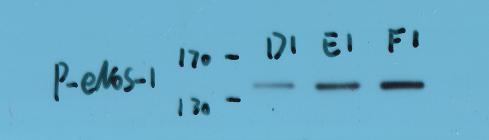


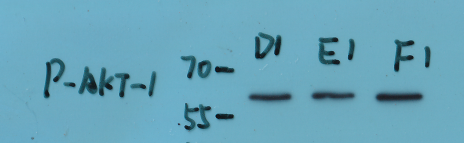


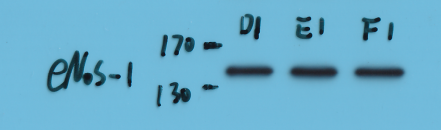


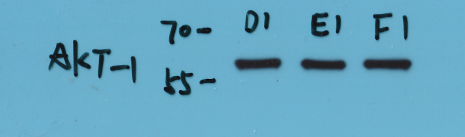


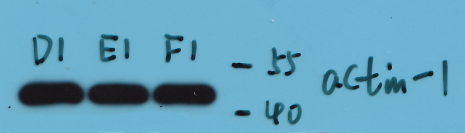


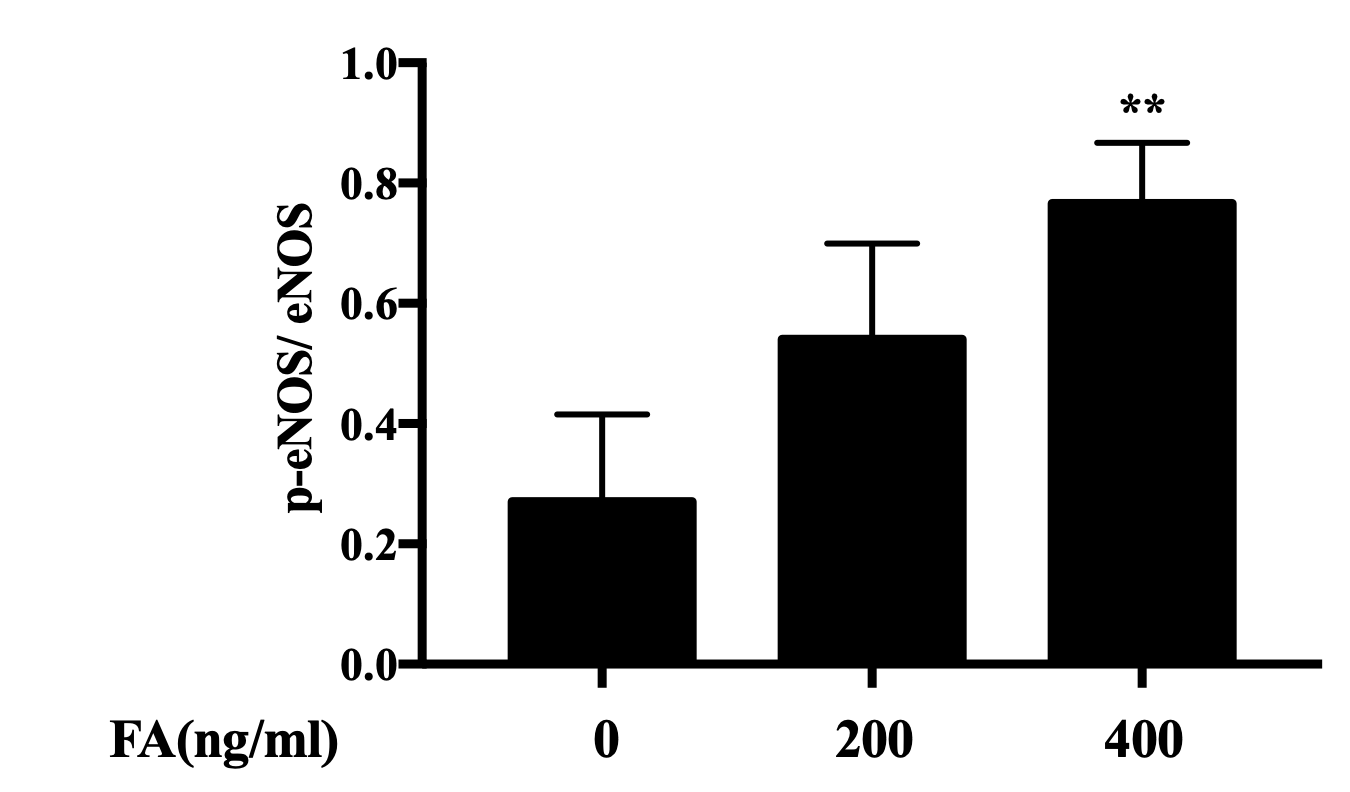

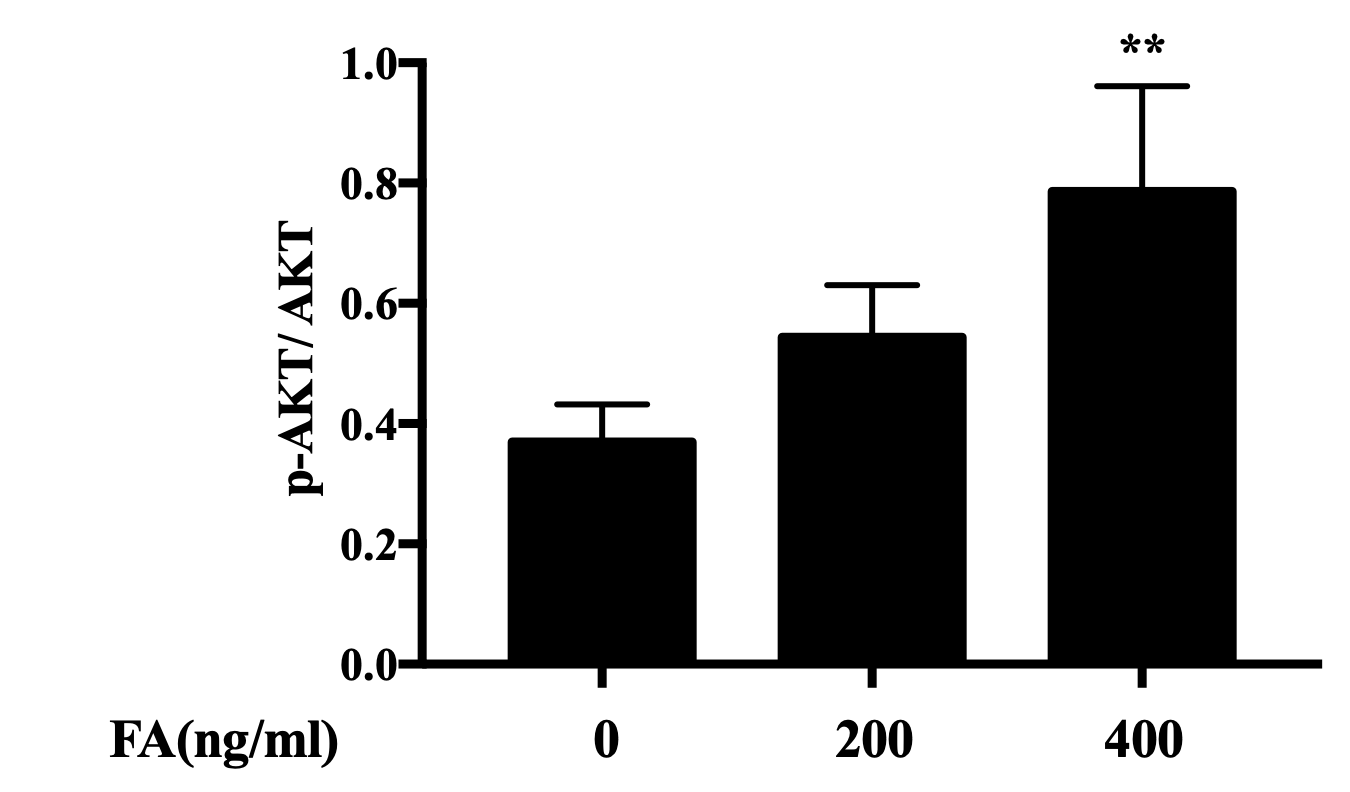

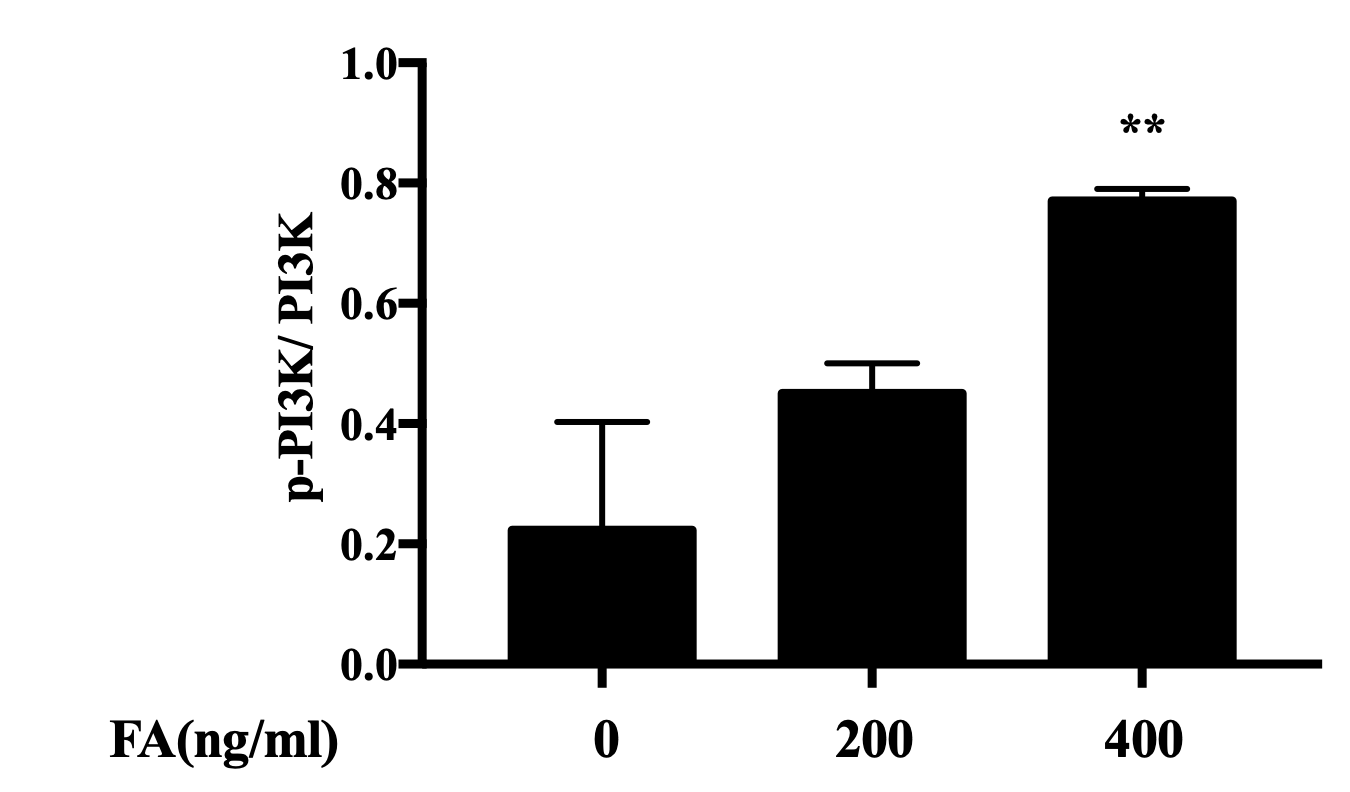

Supplement: Supplementary file 2 — Supplementary file2 (DOCX 1588 KB) [file 12265_2021_10196_MOESM2_ESM.docx]

**Figure 4**

**
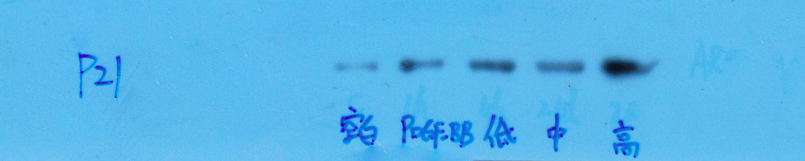

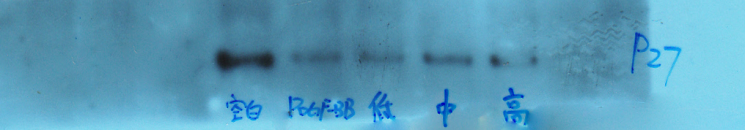
**

**
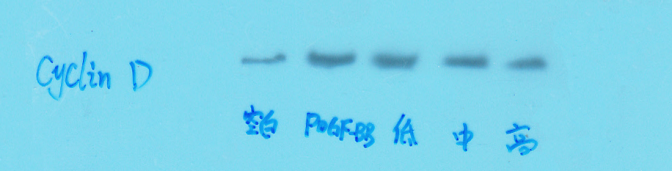
**

**
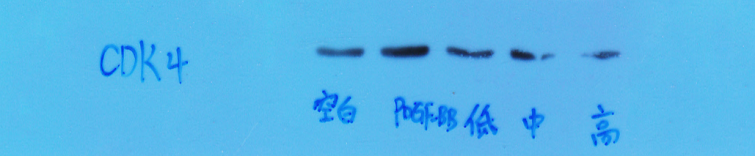
**

**
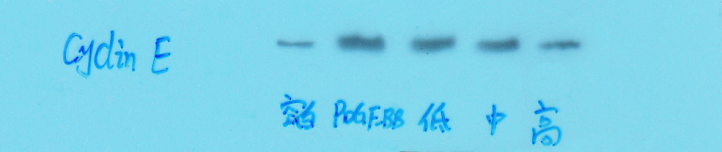
**

**
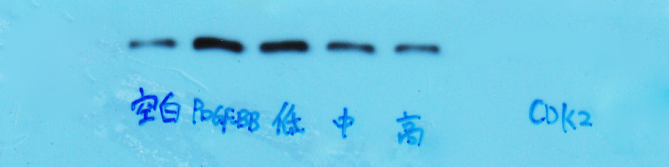
**

**
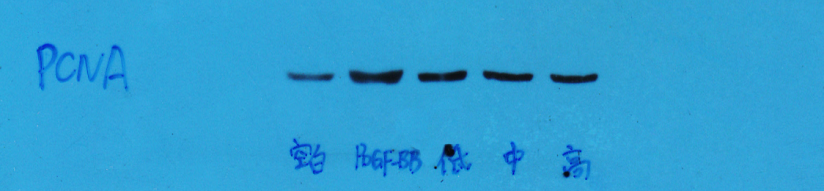

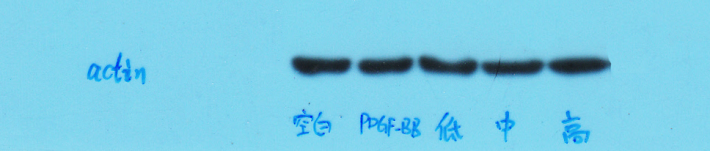
**

| p27 | | | p21 | | |
| --- | --- | --- | --- | --- | --- |
| 0.72 | 0.66 | 0.7 | 0.16 | 0.19 | 0.15 |
| 0.31 | 0.34 | 0.25 | 0.22 | 0.24 | 0.25 |
| 0.31 | 0.26 | 0.32 | 0.24 | 0.2 | 0.27 |
| 0.3 | 0.31 | 0.29 | 0.33 | 0.27 | 0.3 |
| 0.22 | 0.3 | 0.32 | 0.49 | 0.54 | 0.52 |


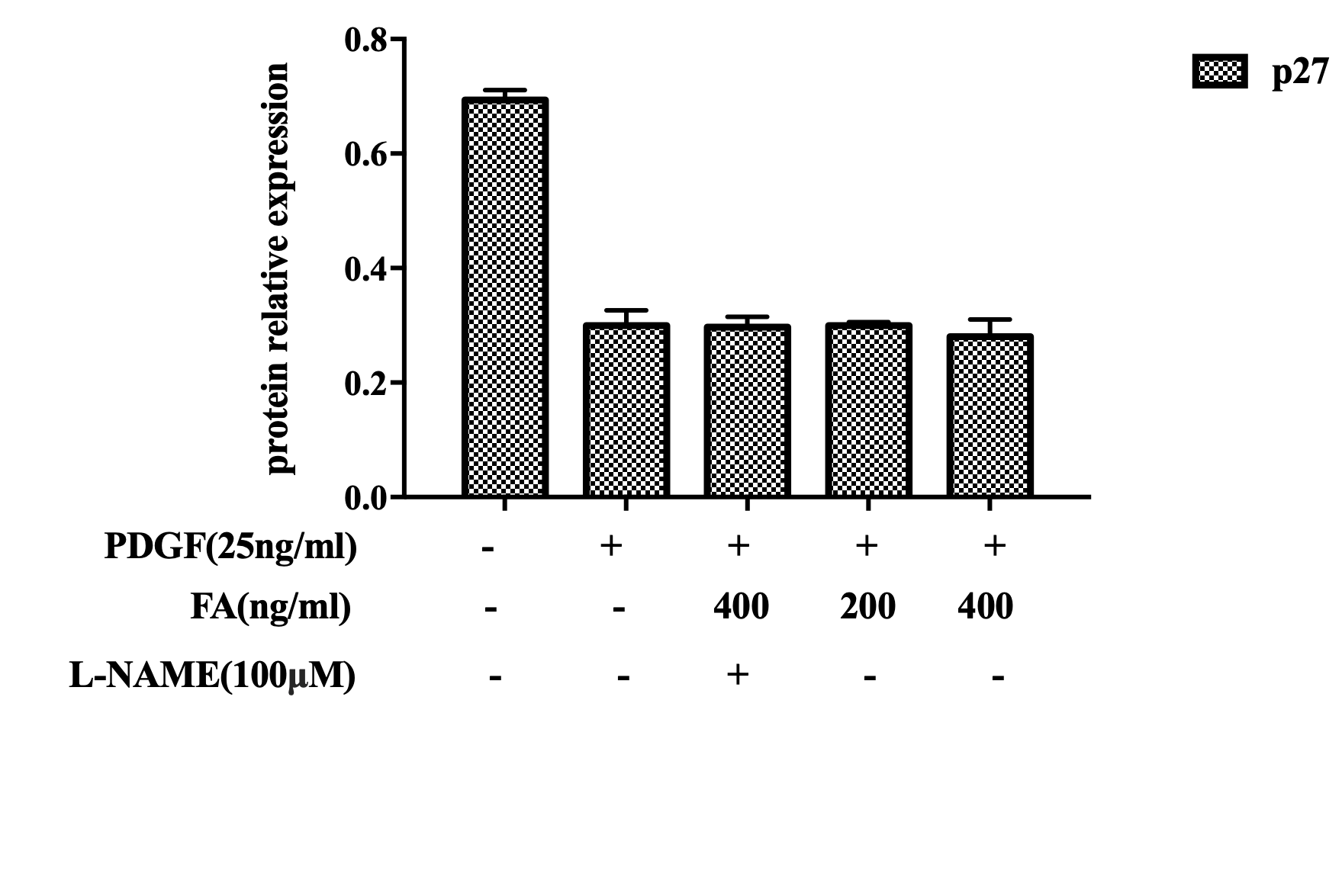


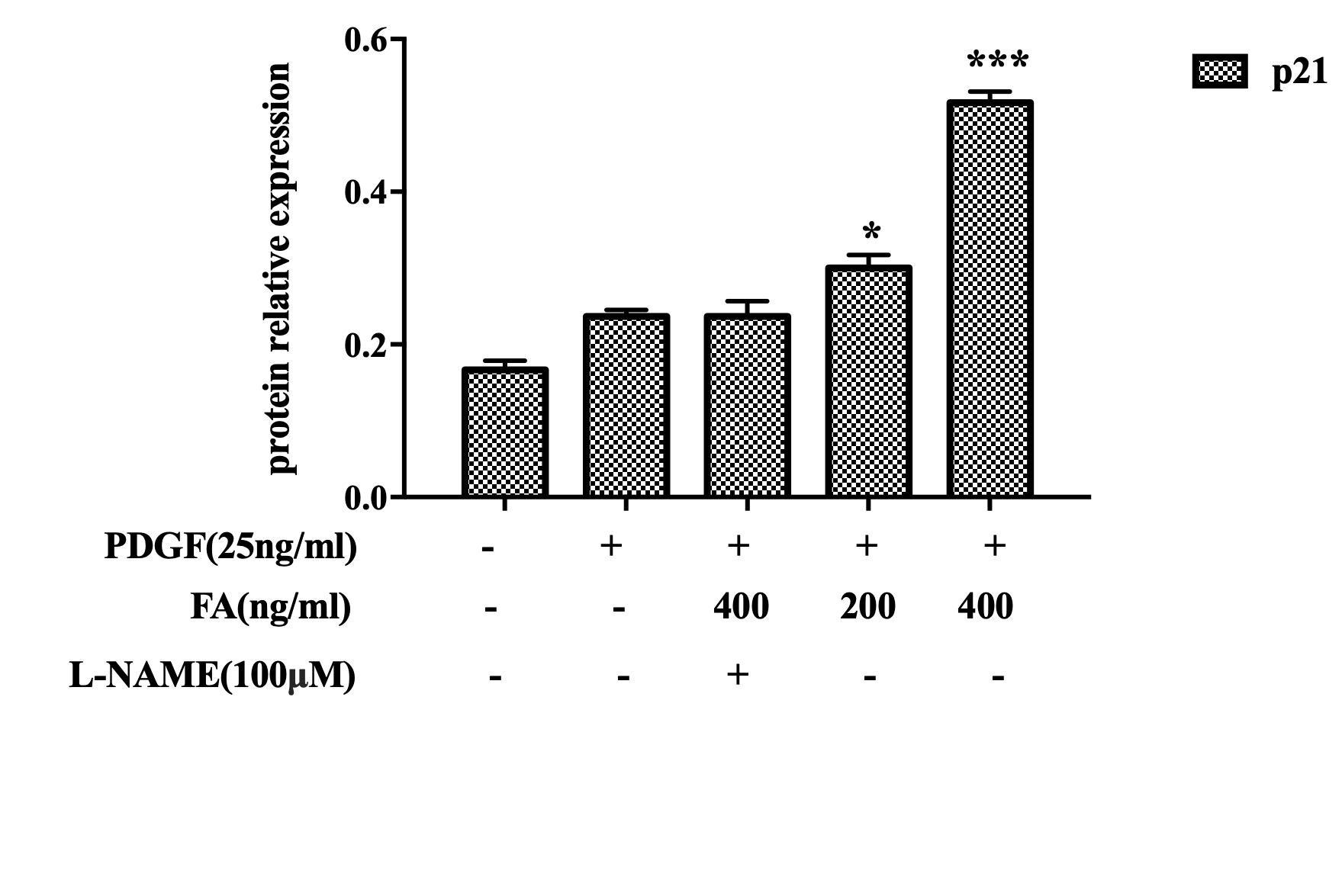


| CDK 2 | | |
| --- | --- | --- |
| 0.24 | 0.22 | 0.23 |
| 0.64 | 0.6 | 0.6 |
| 0.57 | 0.63 | 0.61 |
| 0.31 | 0.3 | 0.3 |
| 0.24 | 0.23 | 0.2 |


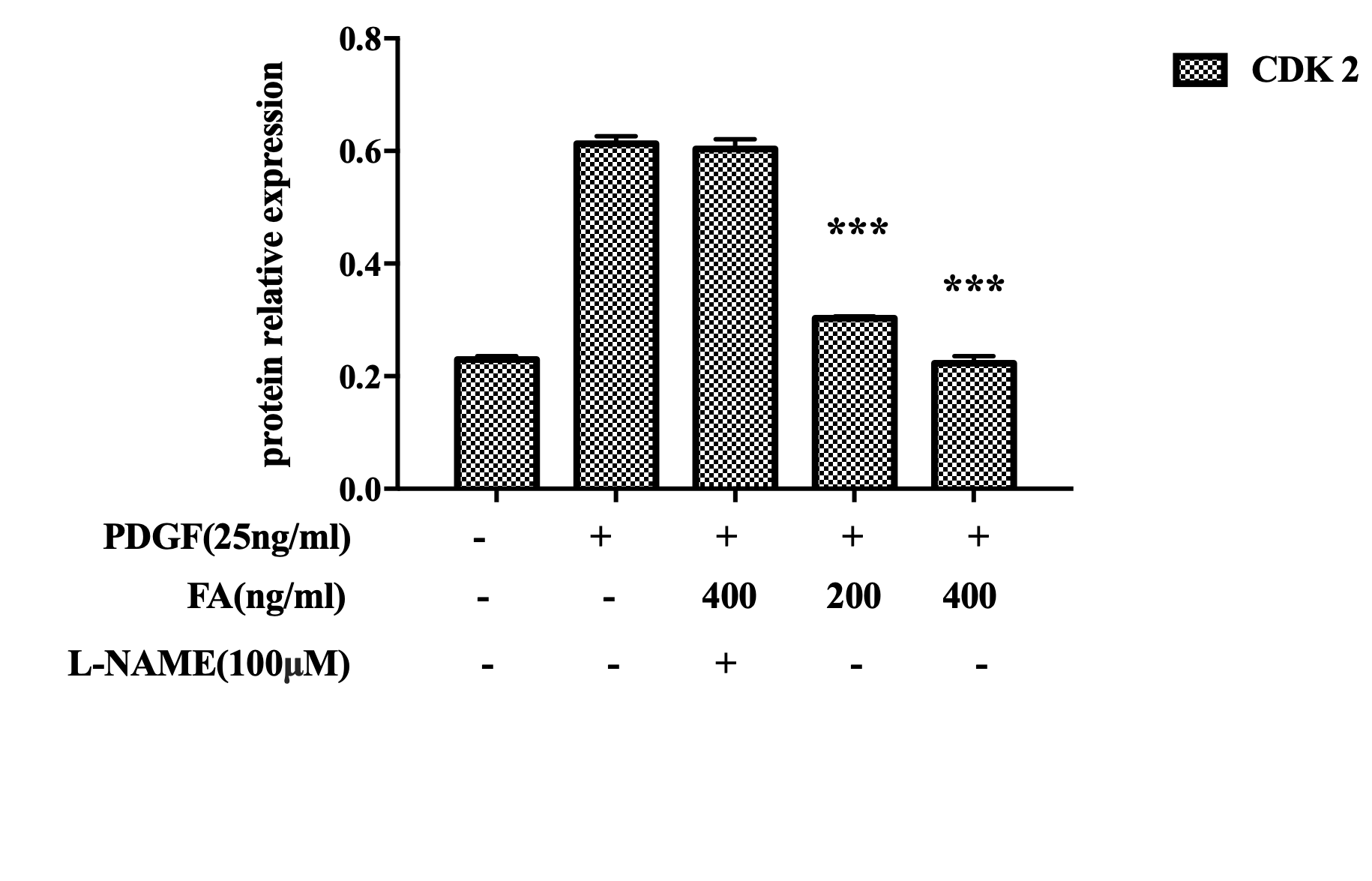


| CDK 4 | | |
| --- | --- | --- |
| 0.27 | 0.23 | 0.26 |
| 0.55 | 0.56 | 0.5 |
| 0.53 | 0.55 | 0.58 |
| 0.32 | 0.3 | 0.29 |
| 0.21 | 0.19 | 0.24 |


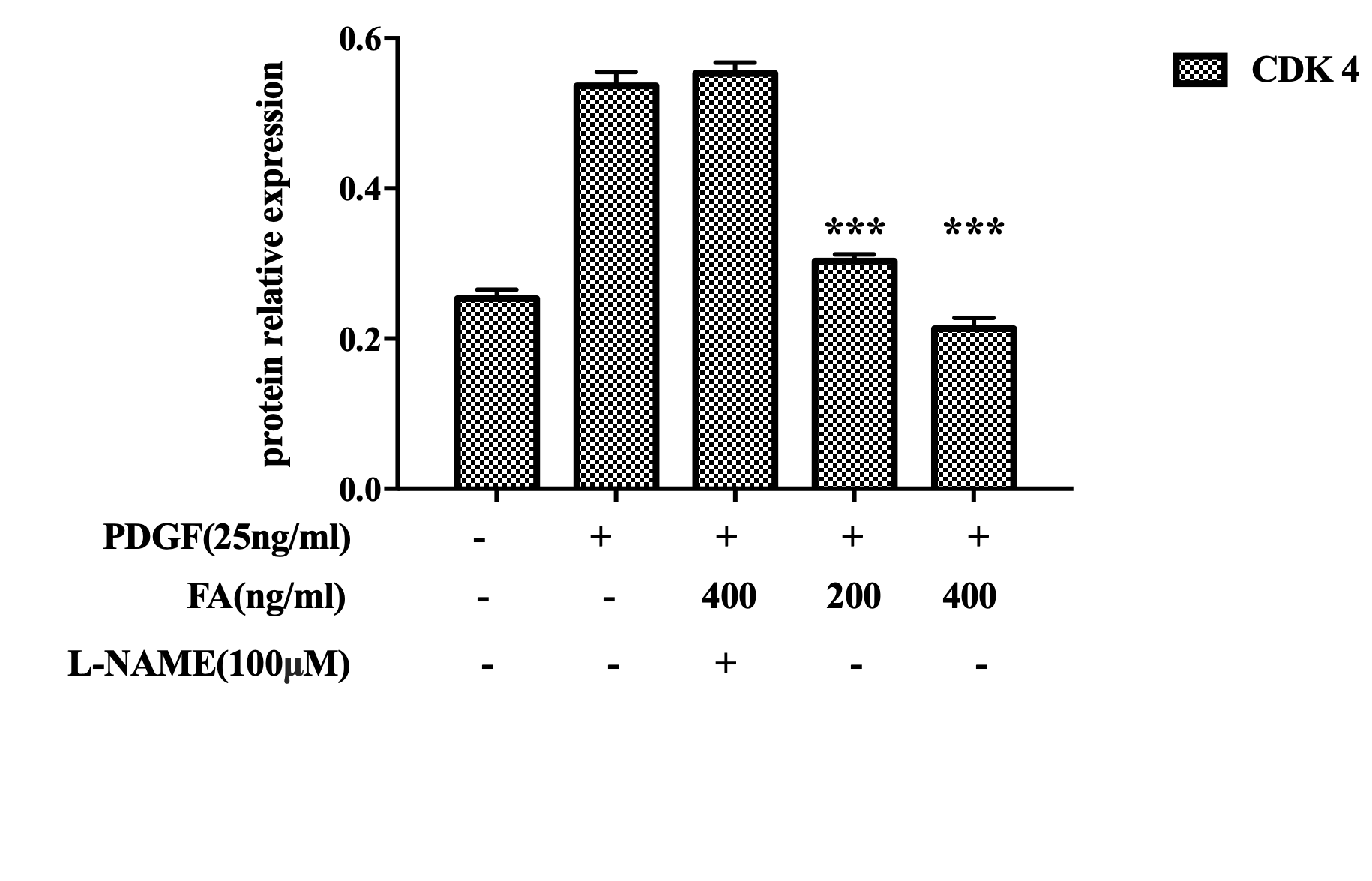


| Cyclin D1 | | |
| --- | --- | --- |
| 0.2 | 0.22 | 0.17 |
| 0.48 | 0.42 | 0.5 |
| 0.42 | 0.49 | 0.48 |
| 0.39 | 0.36 | 0.35 |
| 0.3 | 0.25 | 0.32 |


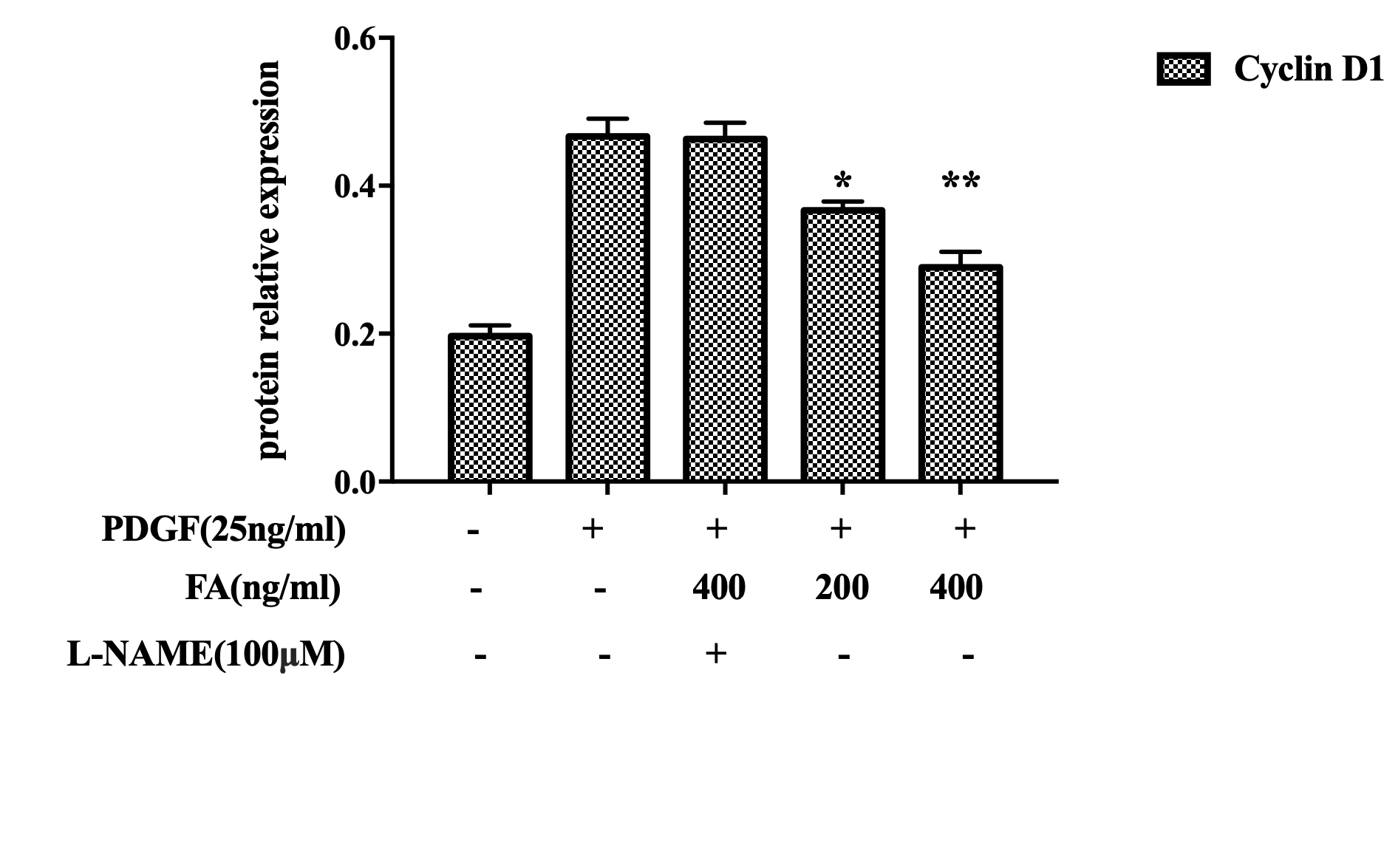


| Cyclin E | | |
| --- | --- | --- |
| 0.19 | 0.2 | 0.18 |
| 0.58 | 0.52 | 0.57 |
| 0.46 | 0.59 | 0.58 |
| 0.41 | 0.39 | 0.43 |
| 0.3 | 0.28 | 0.31 |


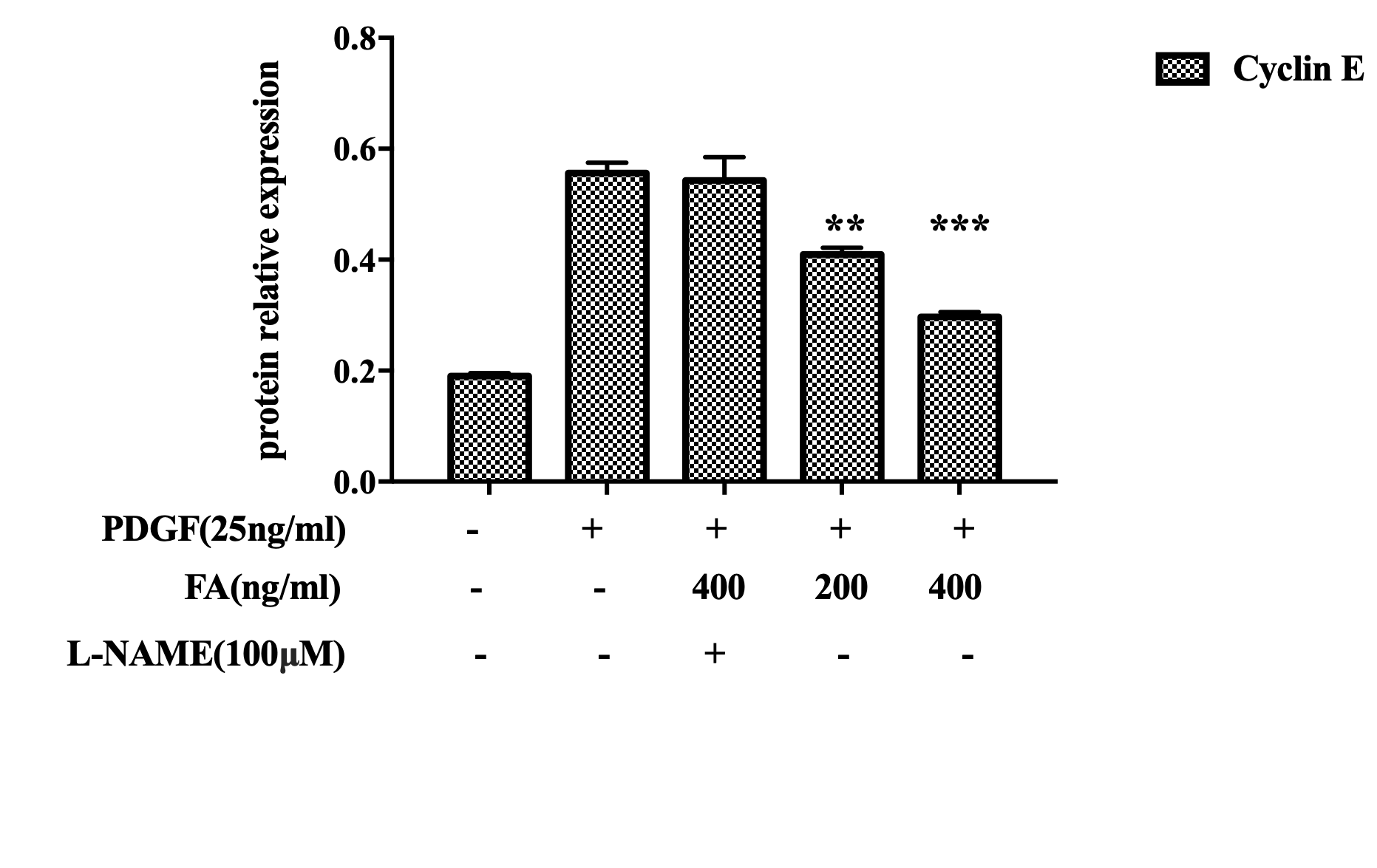


| PCNA | | |
| --- | --- | --- |
| 0.21 | 0.19 | 0.2 |
| 0.51 | 0.5 | 0.52 |
| 0.45 | 0.51 | 0.53 |
| 0.35 | 0.39 | 0.4 |
| 0.32 | 0.33 | 0.29 |


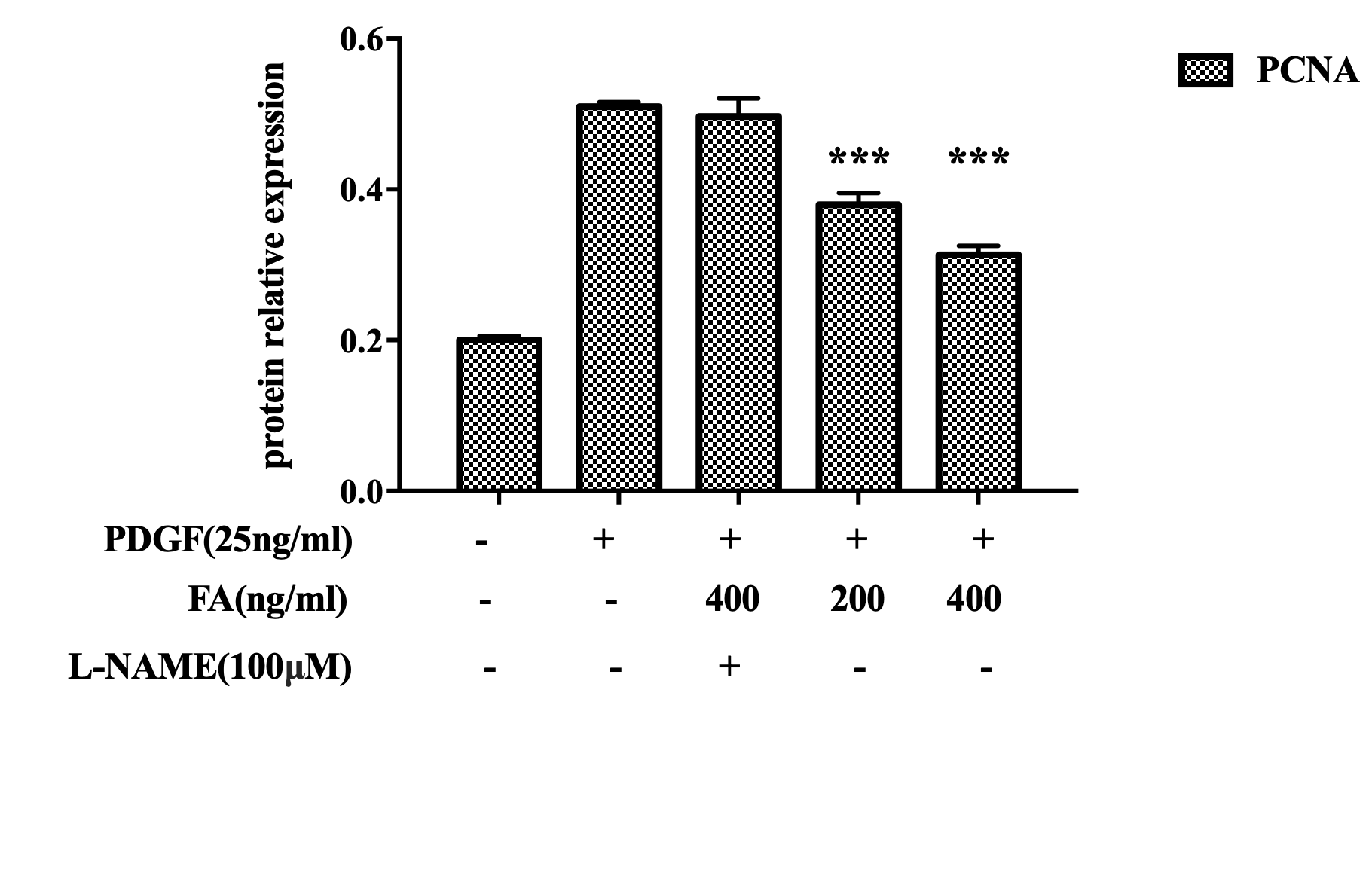

Supplement: Supplementary file 4 — Supplementary file4 (DOCX 2204 KB) [file 12265_2021_10196_MOESM4_ESM.docx]

**Figure 7**

**
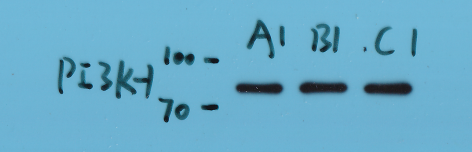
**

**
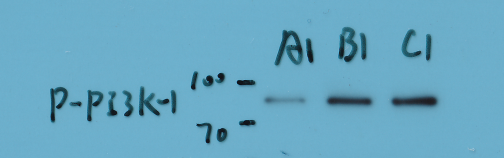
**

**
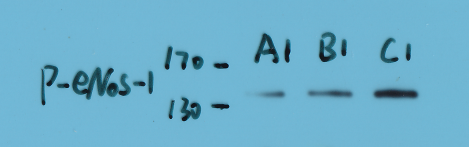
**

**
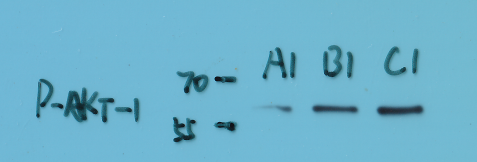
**

**
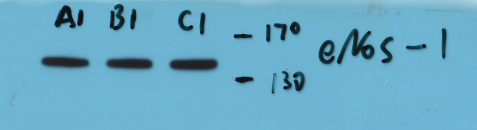
**

**
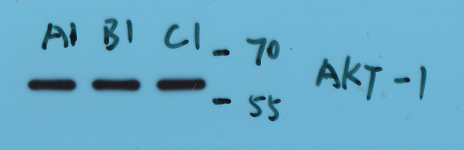
**

**
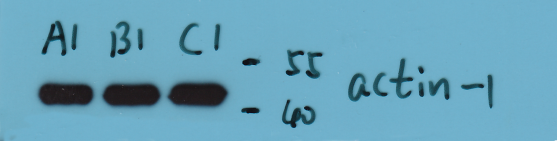
**


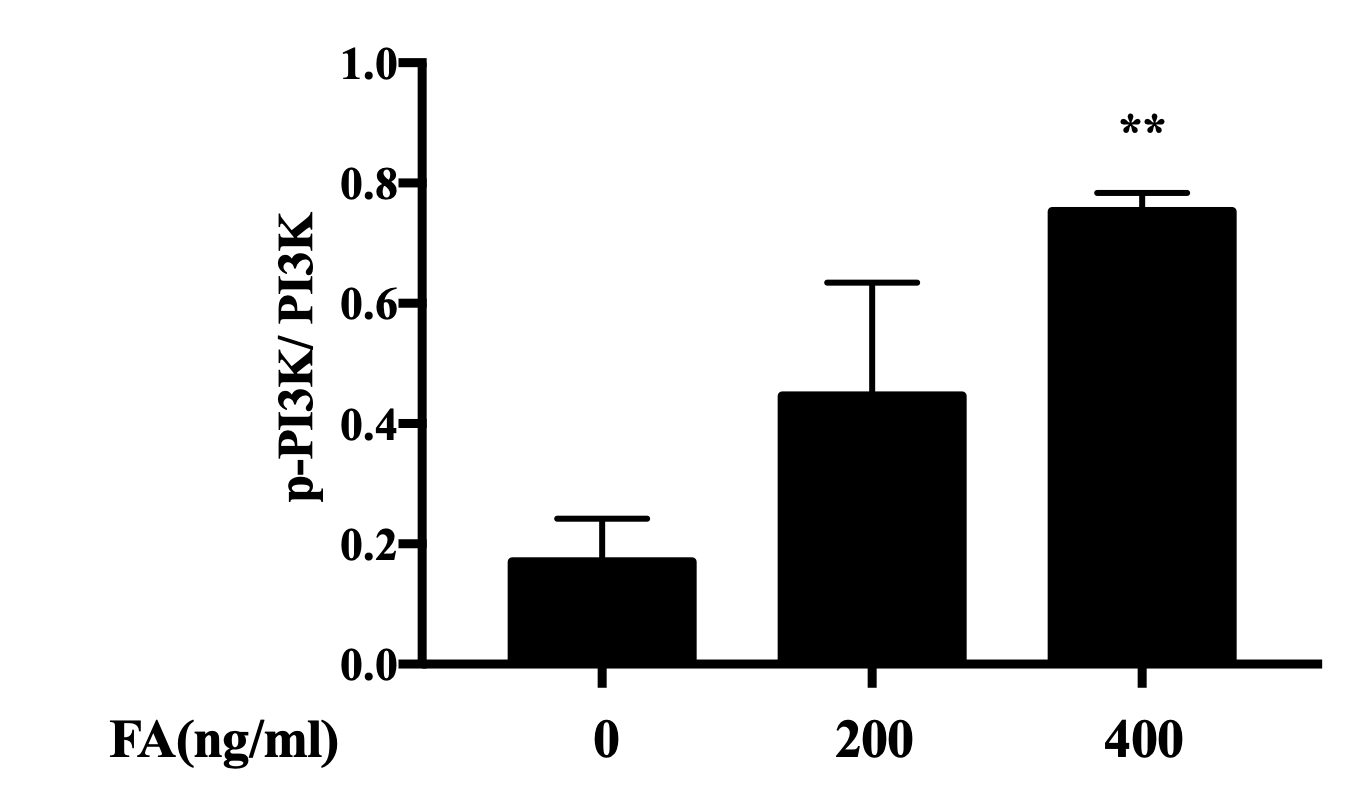

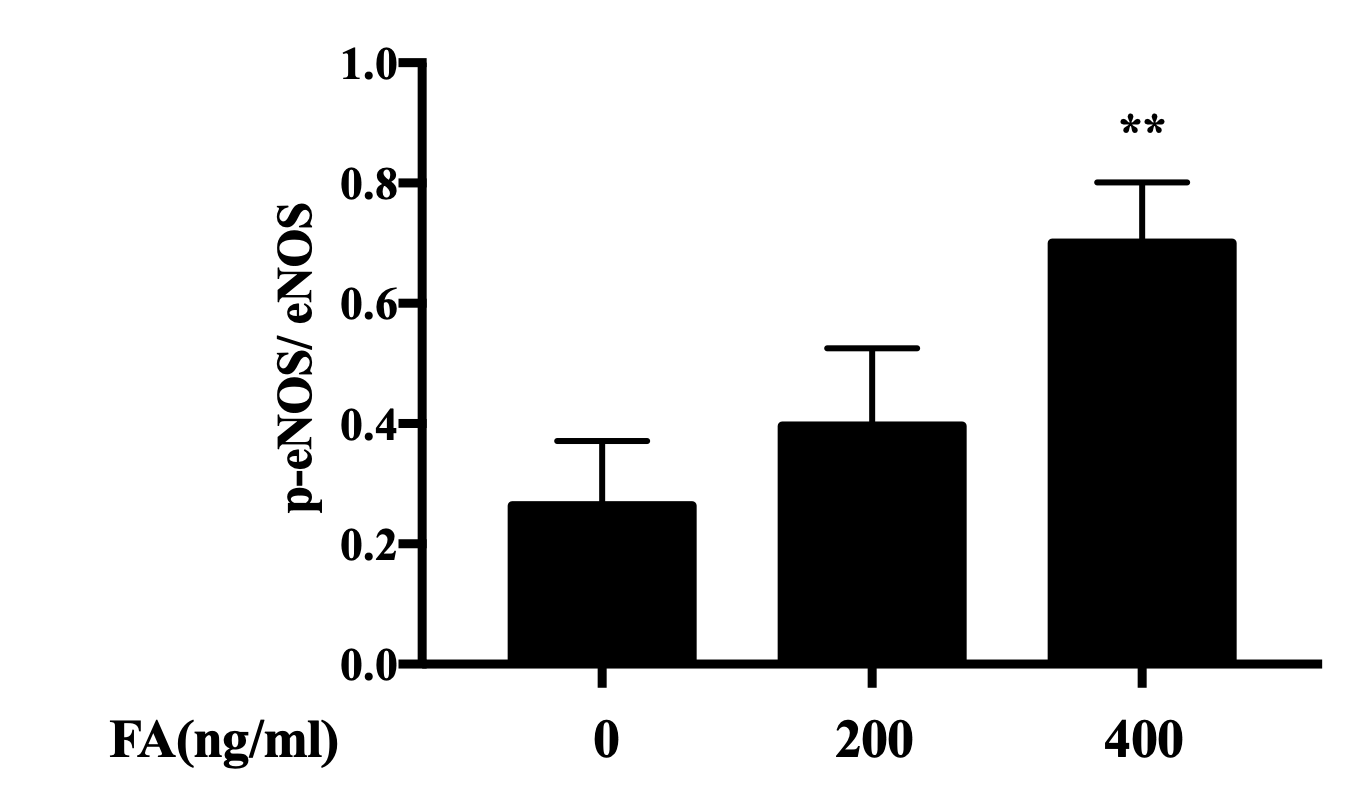

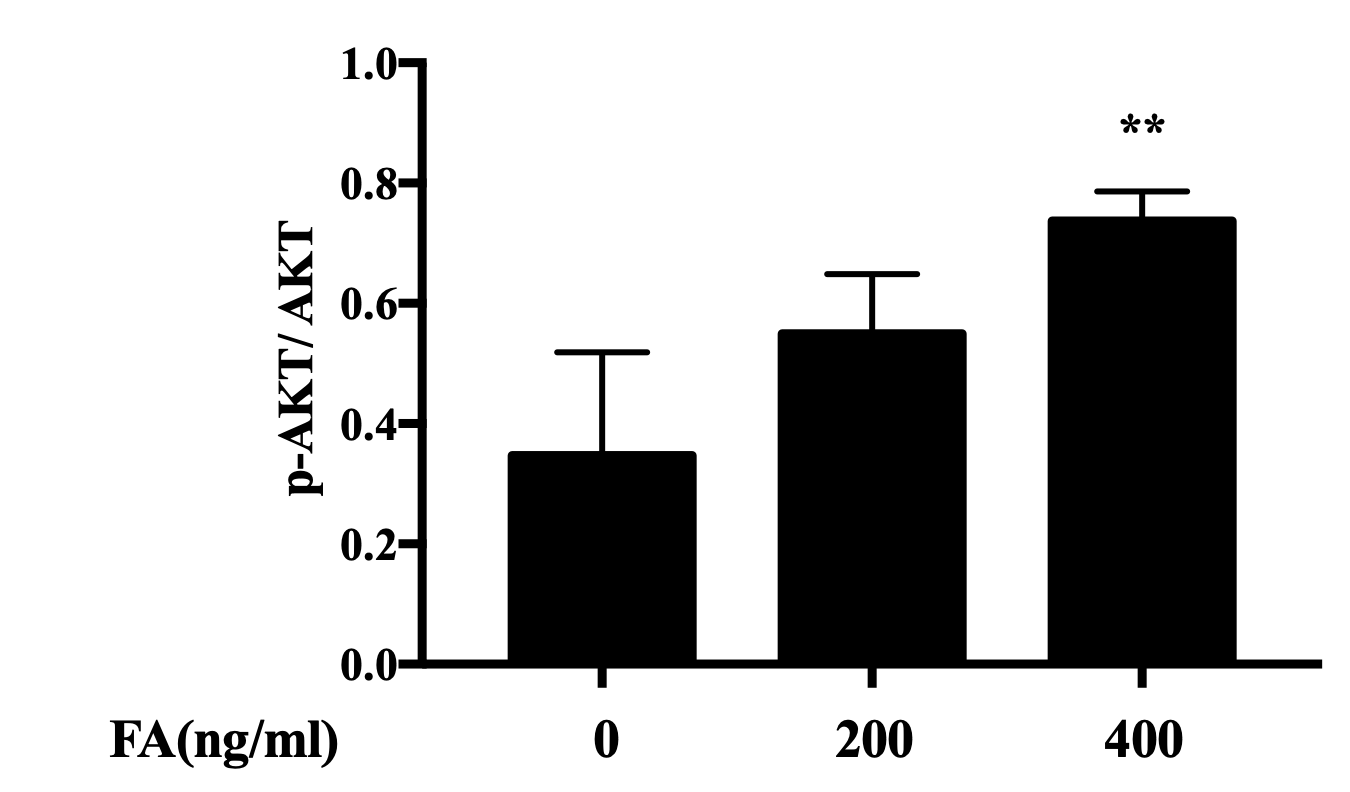

Supplement: Supplementary file 7 — Supplementary file7 (DOCX 880 KB) [file 12265_2021_10196_MOESM7_ESM.docx]
