# Supplementary material for: Ferulic Acid Alleviates Atherosclerotic Plaques by Inhibiting VSMC Proliferation Through the NO/p21 Signaling pathway
Source: J Cardiovasc Transl Res. 2022 Jan 6;15(4):865–75. doi: 10.1007/s12265-021-10196-8 (PMC9622559; doi:10.1007/s12265-021-10196-8)
Supplement: Supplementary file 3 — Supplementary file3 (DOCX 661 KB) [file 12265_2021_10196_MOESM3_ESM.docx]

**Figure 3A**

| Control | PDGF | PDGF+400+L-N | PDGF+200 | PDGF+400 |
| --- | --- | --- | --- | --- |
| 0.2278 | 0.3487 | 0.3446 | 0.2889 | 0.2578 |
| 0.2306 | 0.3518 | 0.3467 | 0.2844 | 0.2489 |
| 0.2286 | 0.3491 | 0.3389 | 0.2816 | 0.2546 |
| 0.2198 | 0.3525 | 0.3368 | 0.2795 | 0.2517 |
| 0.2347 | 0.3574 | 0.3363 | 0.2847 | 0.249 |
| 0.2283 | 0.3519 | 0.3407 | 0.2838 | 0.2524 |

**
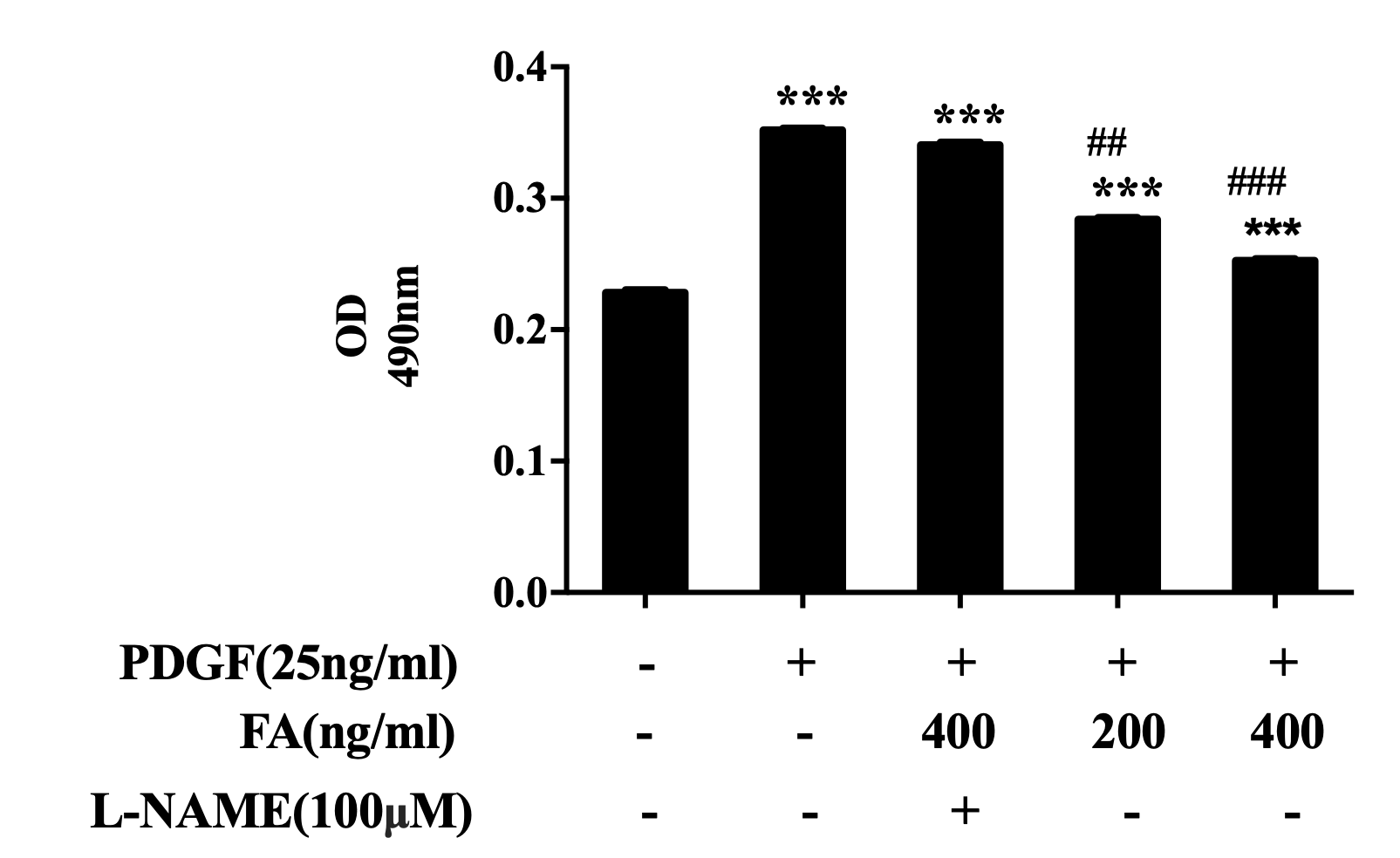
**

**Figure 3B**

**
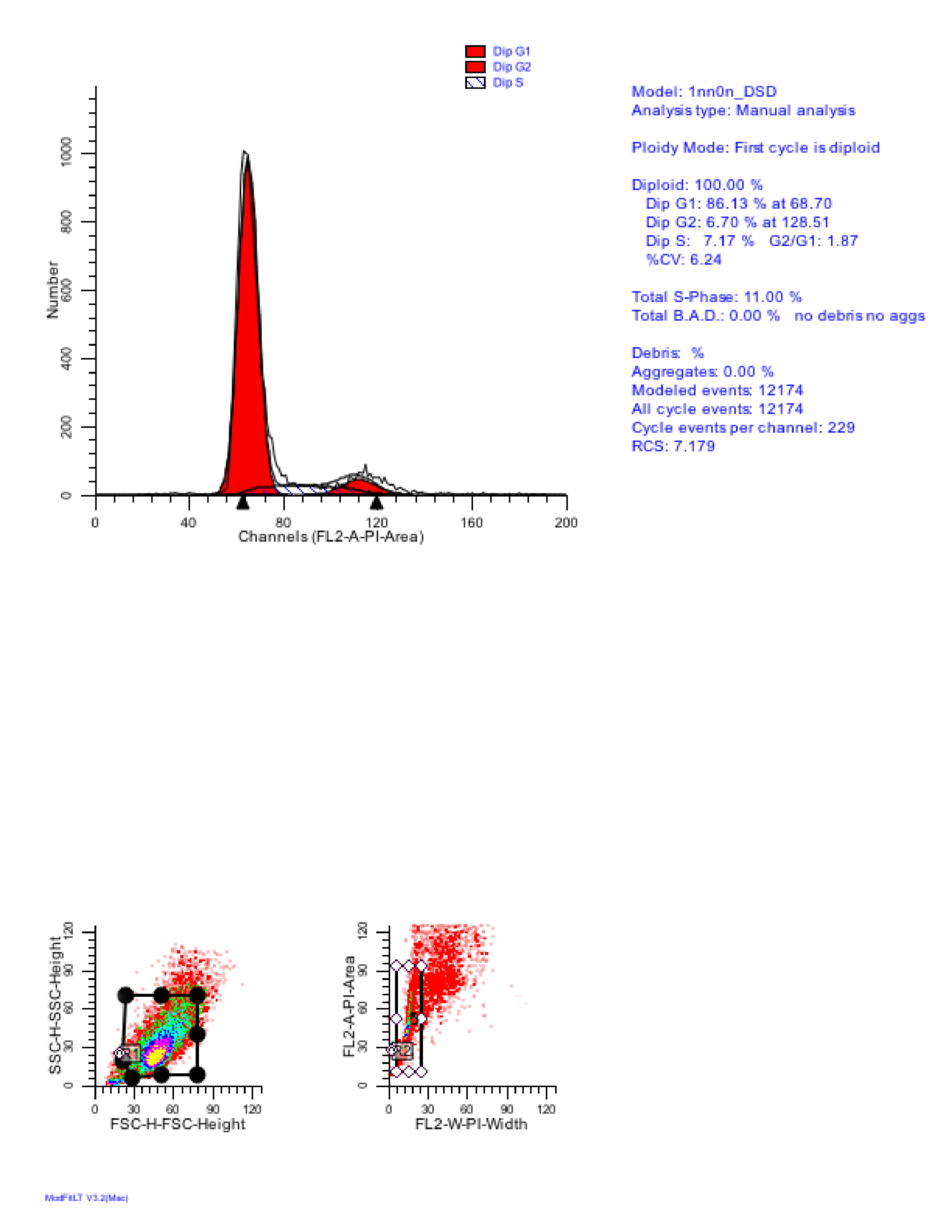
**

**Starvation**


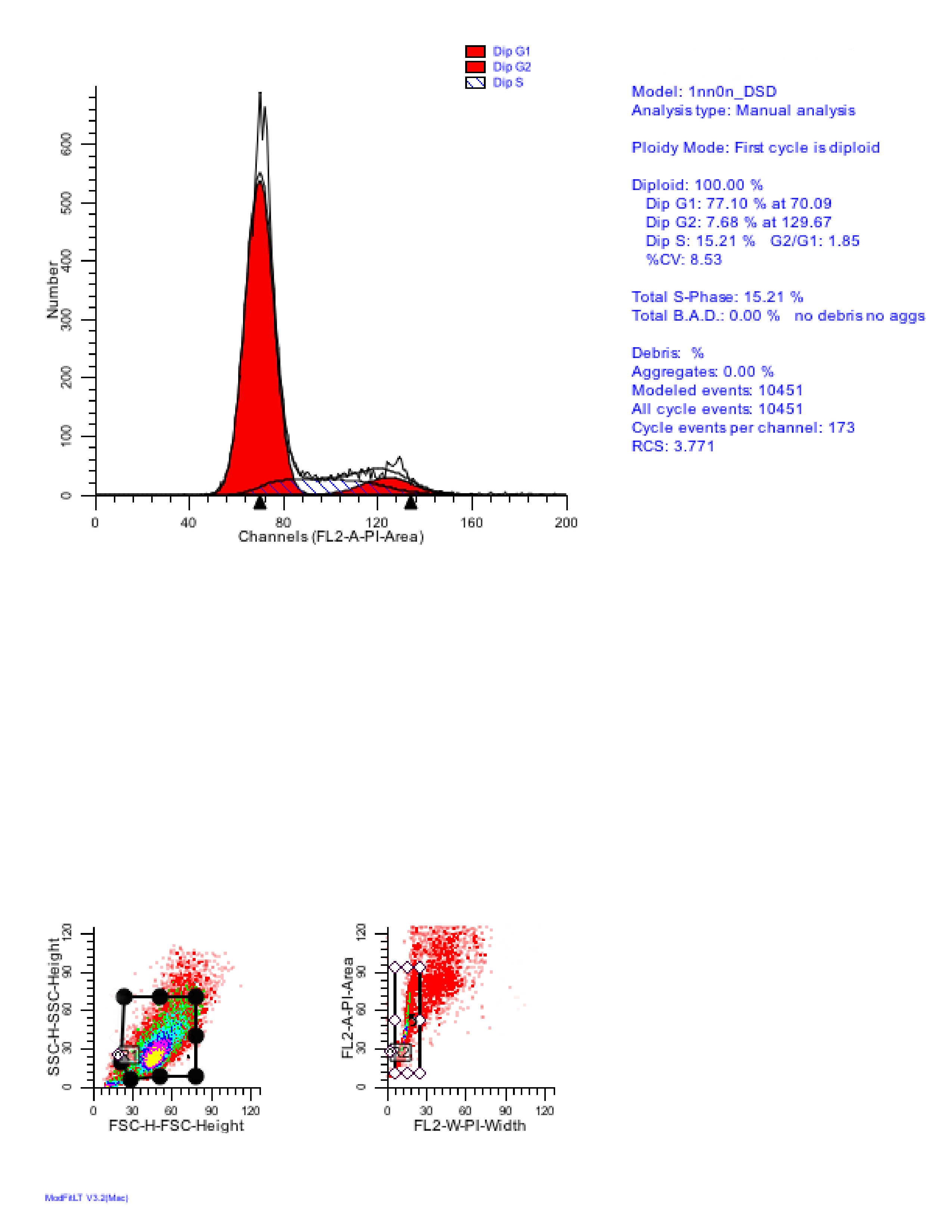
**PDGF**

**
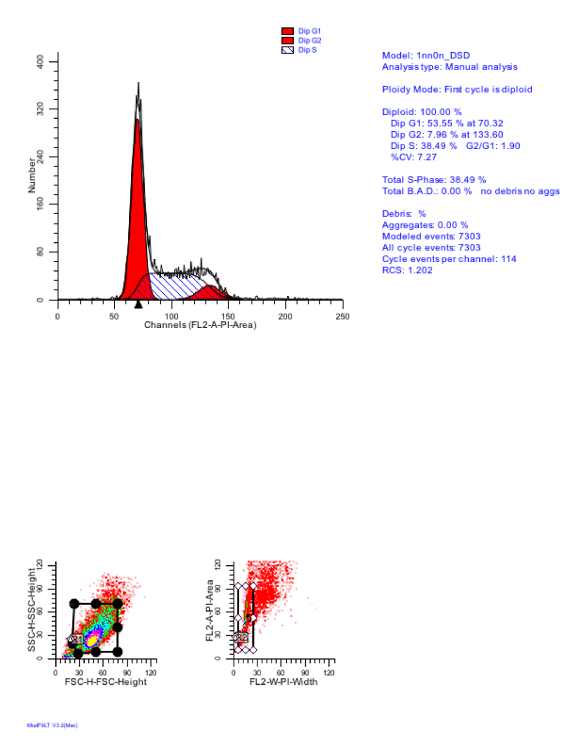
PDGF+FA400+L-NAME**

**
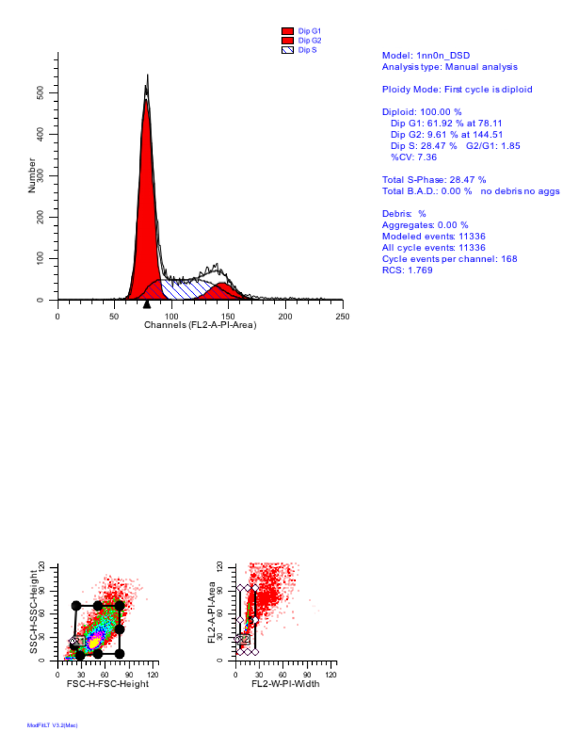
PDGF+FA200**

**
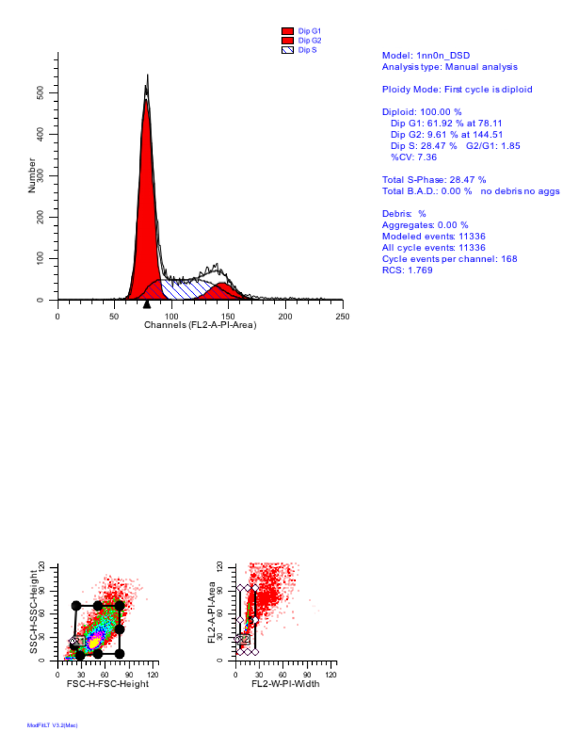
PDGF+FA400**

**Figure 3C**

|  | G0/G1 | | | S | | | G2/M | | |
| --- | --- | --- | --- | --- | --- | --- | --- | --- | --- |
| control | 77.1 | 75.47 | 75.83 | 15.21 | 16.33 | 16.01 | 7.68 | 8.2 | 8.16 |
| PDGF | 53.55 | 54.17 | 52.84 | 38.49 | 36.98 | 39.05 | 7.96 | 8.85 | 8.11 |
| 400+L-N | 61.92 | 55.14 | 54.47 | 28.47 | 33.31 | 35.99 | 9.61 | 11.55 | 9.54 |
| 200 | 65.57 | 66.23 | 67.25 | 28.09 | 26.78 | 27.43 | 6.35 | 6.99 | 5.32 |
| 400 | 70.33 | 74.69 | 75.27 | 17.89 | 14.43 | 17.05 | 11.78 | 10.88 | 7.68 |

**
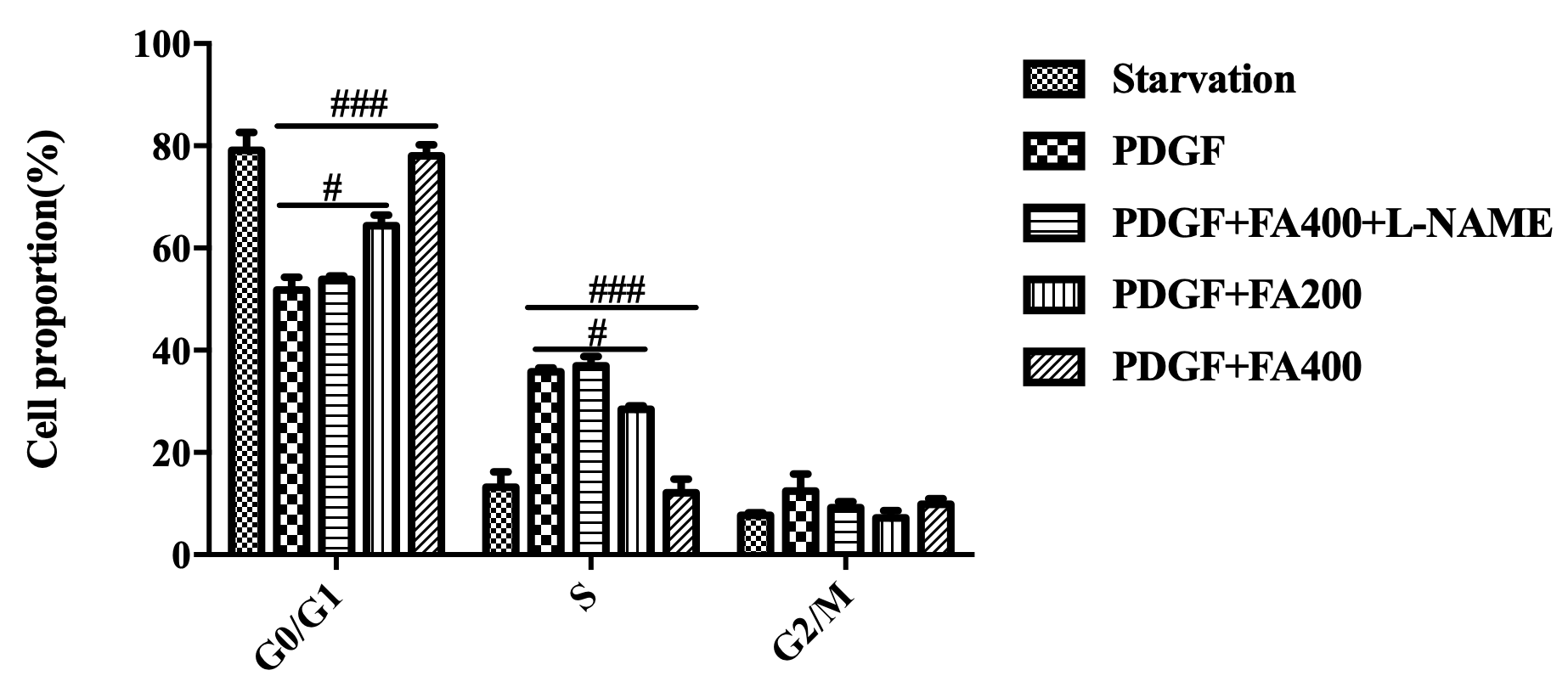
**
