# Supplementary material for: Ferulic Acid Alleviates Atherosclerotic Plaques by Inhibiting VSMC Proliferation Through the NO/p21 Signaling pathway
Source: J Cardiovasc Transl Res. 2022 Jan 6;15(4):865–75. doi: 10.1007/s12265-021-10196-8 (PMC9622559; doi:10.1007/s12265-021-10196-8)
Supplement: Supplementary file 5 — Supplementary file5 (DOCX 4353 KB) [file 12265_2021_10196_MOESM5_ESM.docx]

**Figure 5**

**PDGF**

**
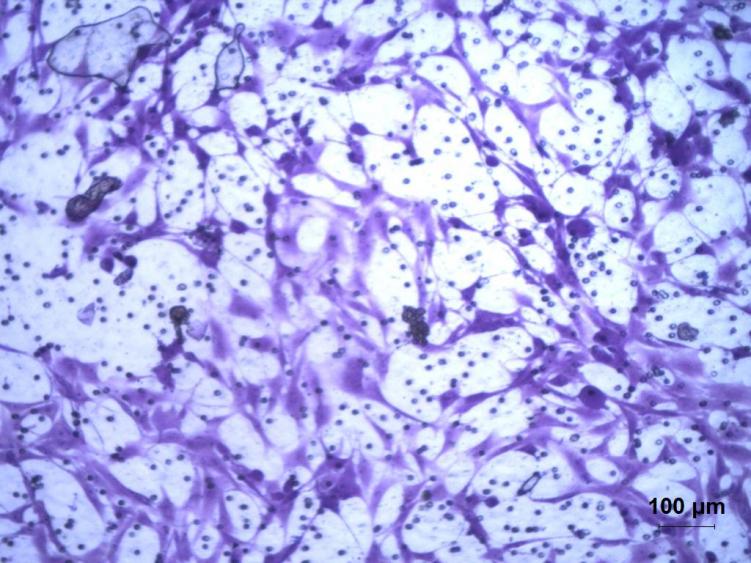

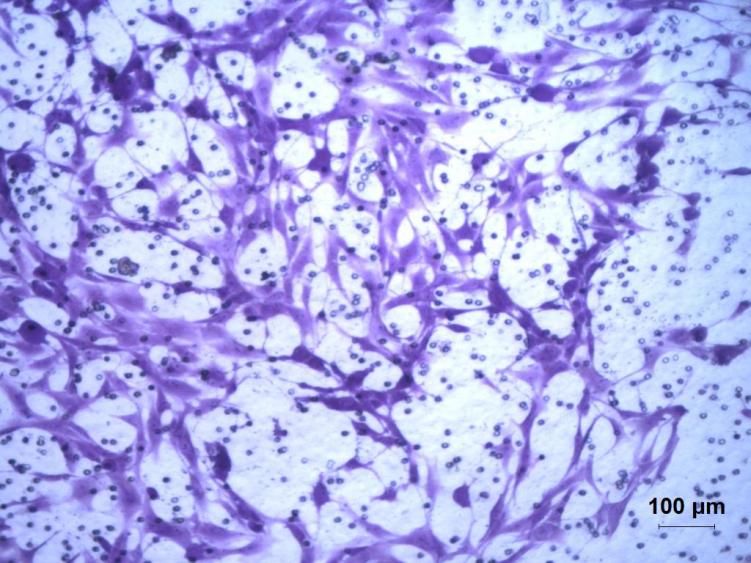
**

**
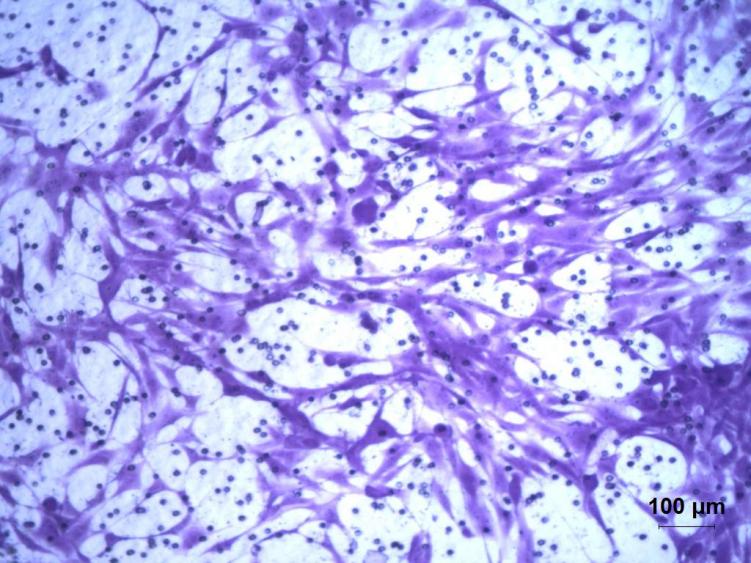
**

**PDGF+FA(400ng/ml)**

**
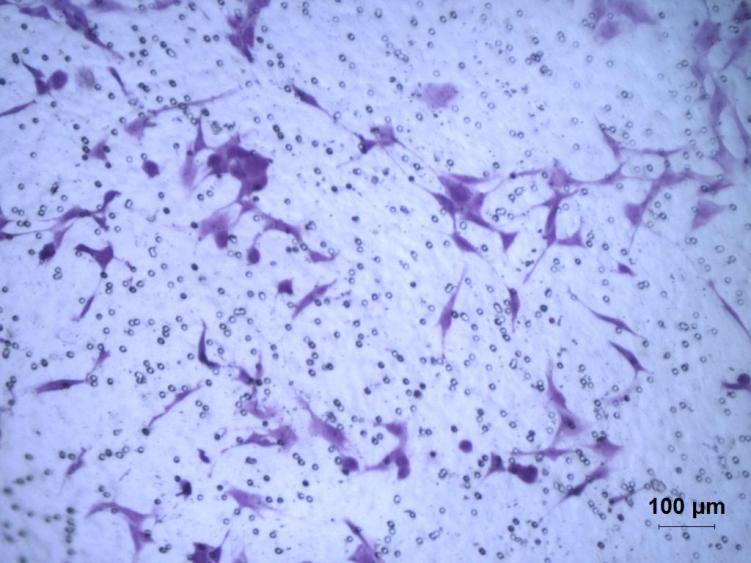

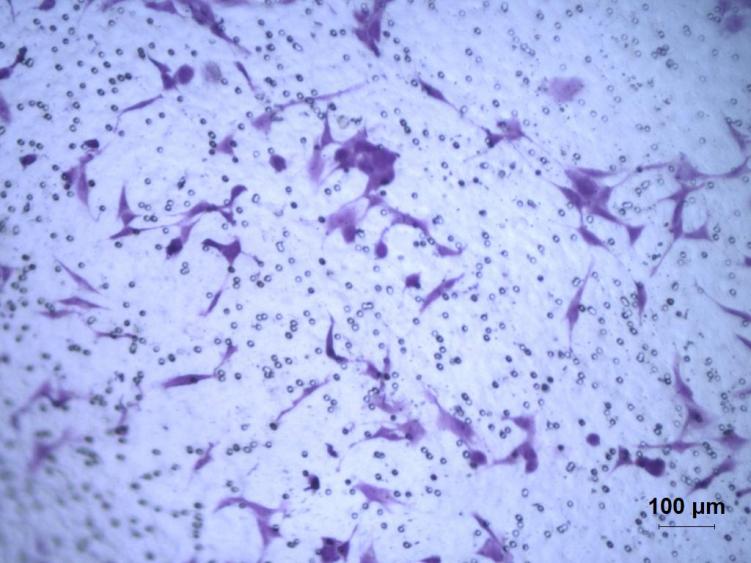

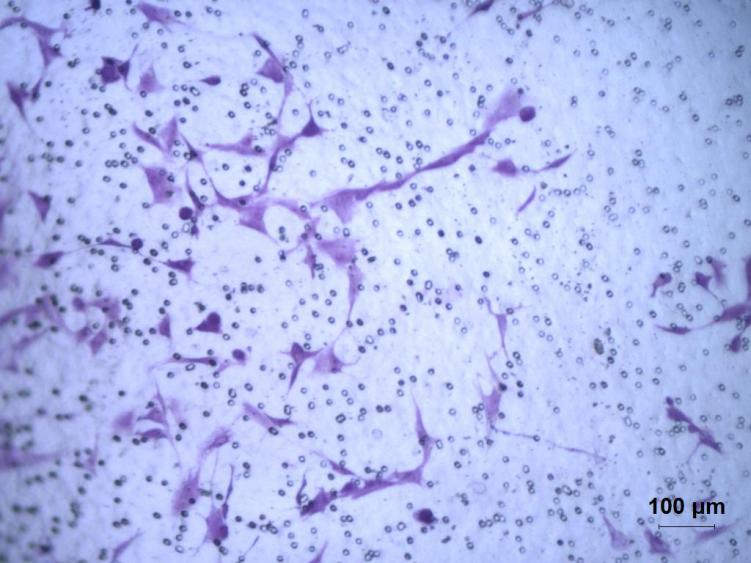
**

**PDGF+FA(200ng/ml)**

**
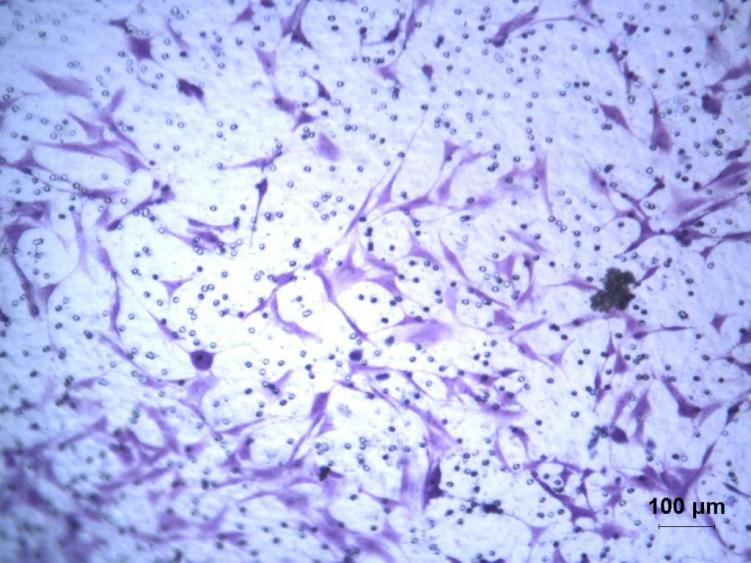

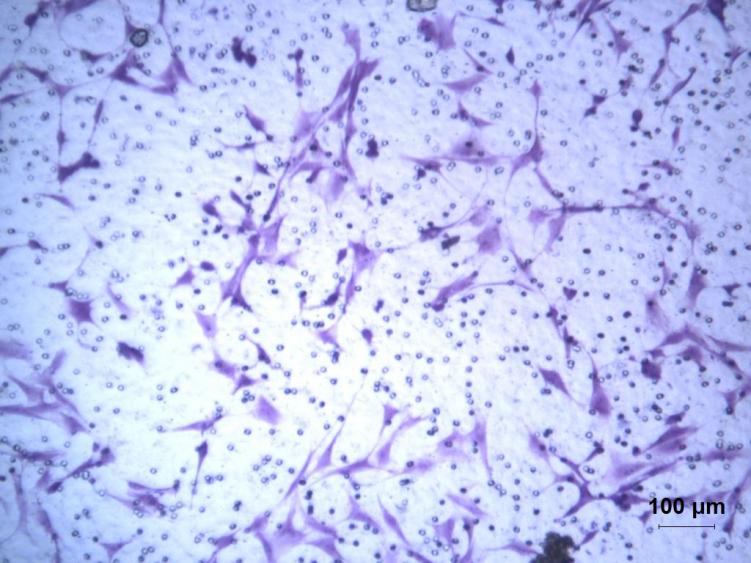

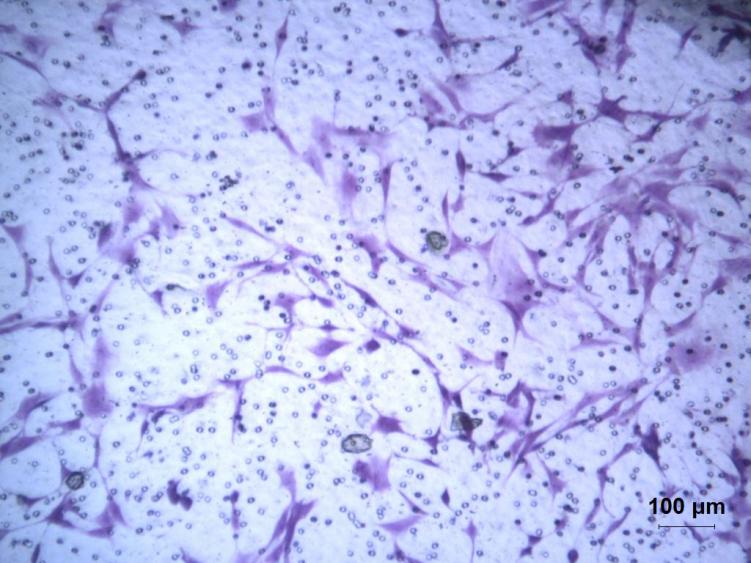
**

**Untreated cell**

**
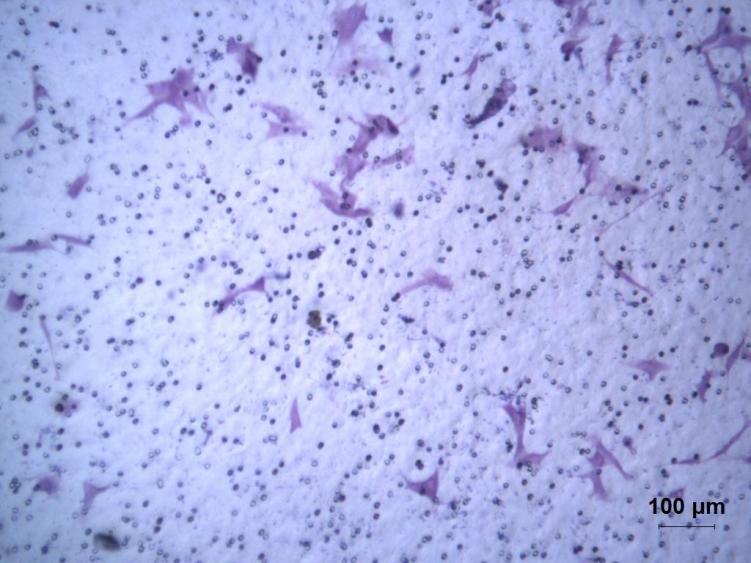

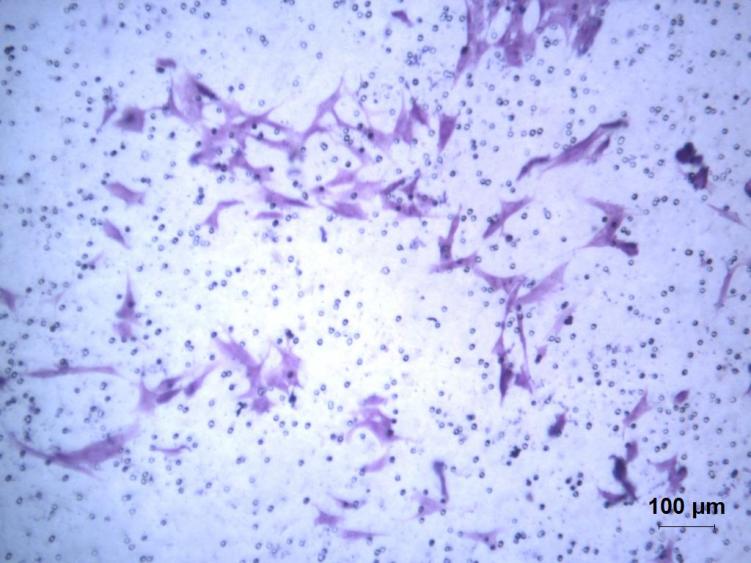

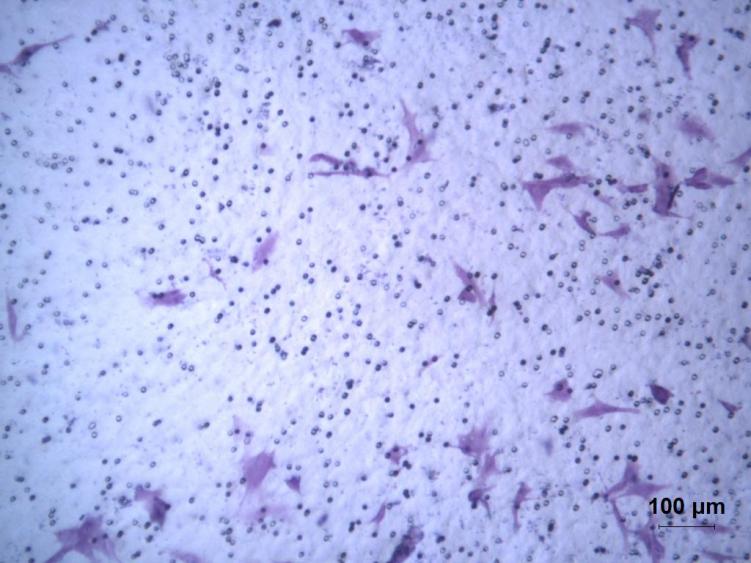
**

| Untreated cell | PDGF | PDGF+200 | PDGF+400 |
| --- | --- | --- | --- |
| 45 | 266 | 158 | 85 |
| 47 | 238 | 155 | 73 |
| 65 | 243 | 132 | 88 |

**
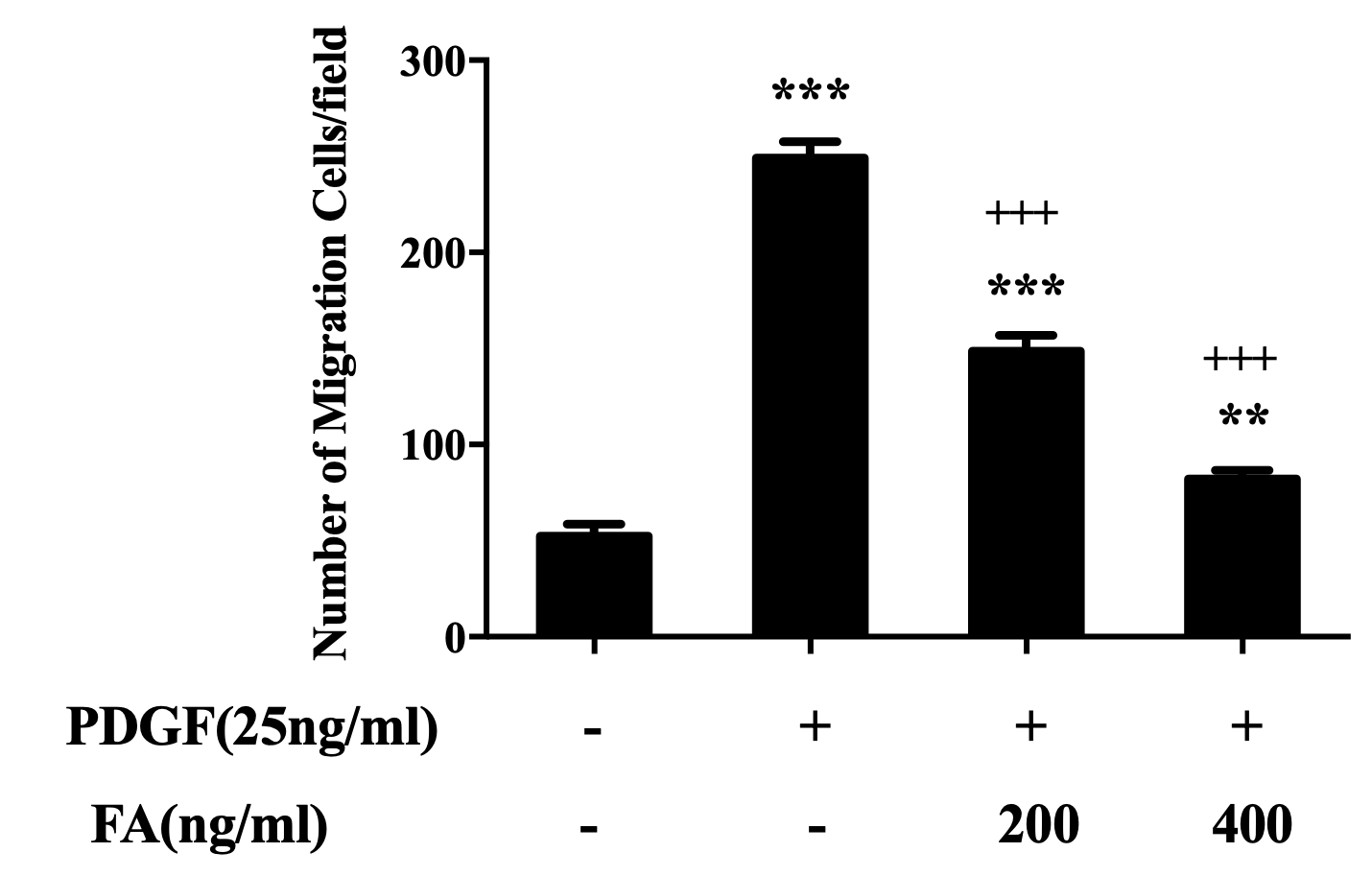
**

**PDGF**

**
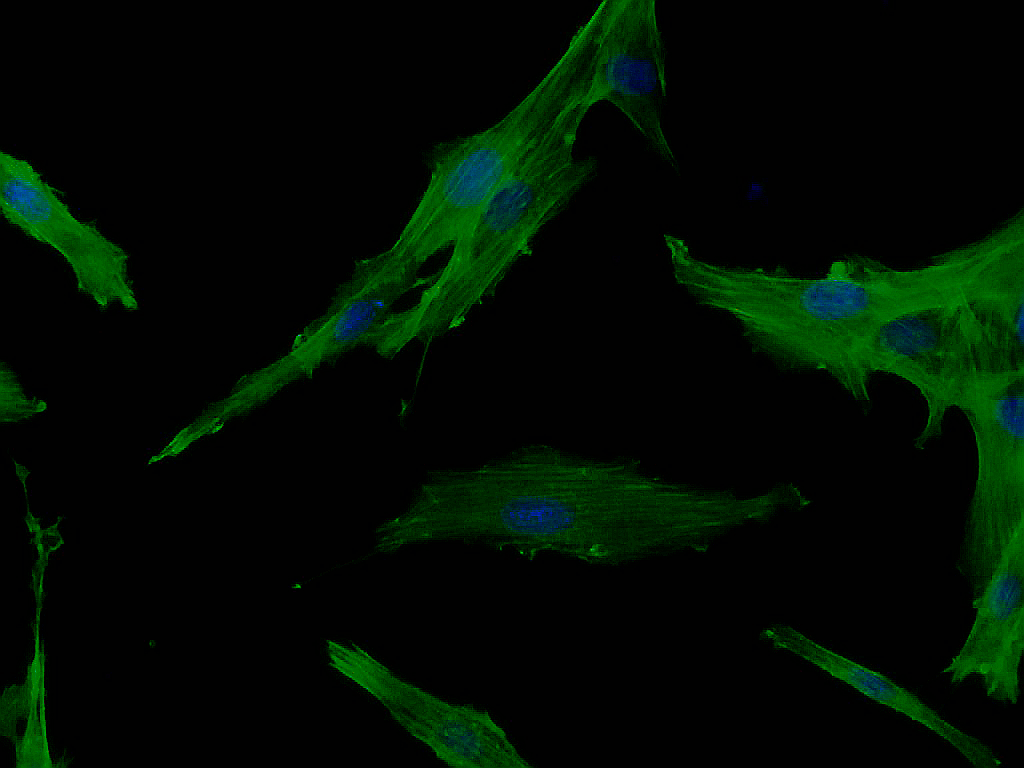

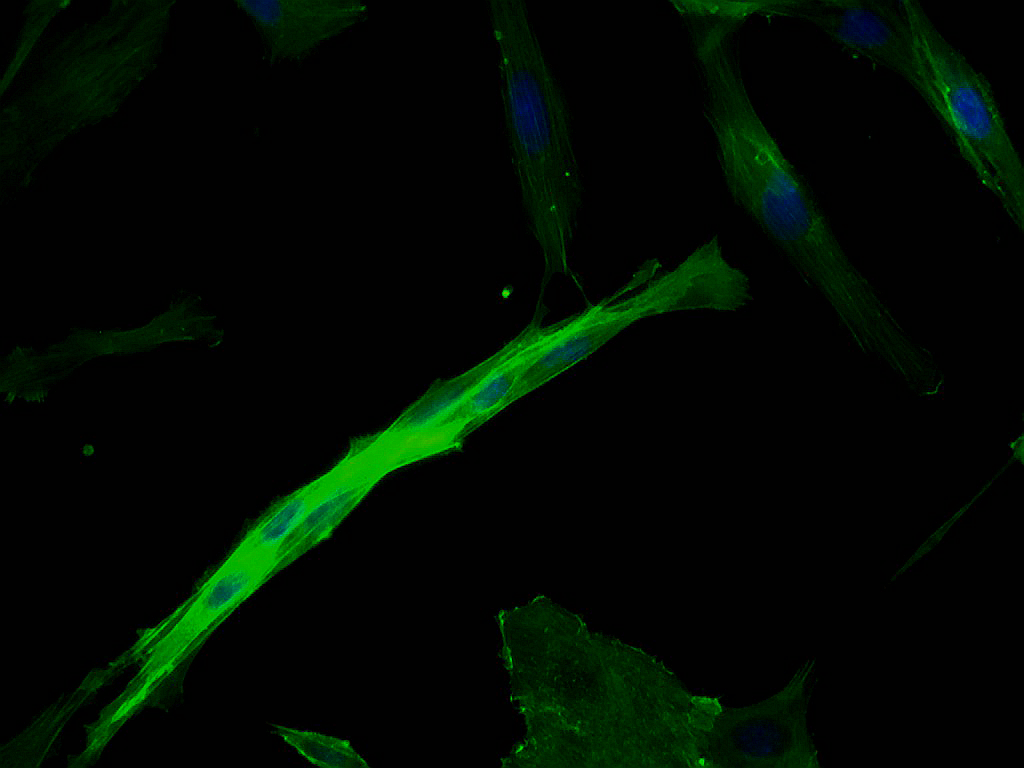

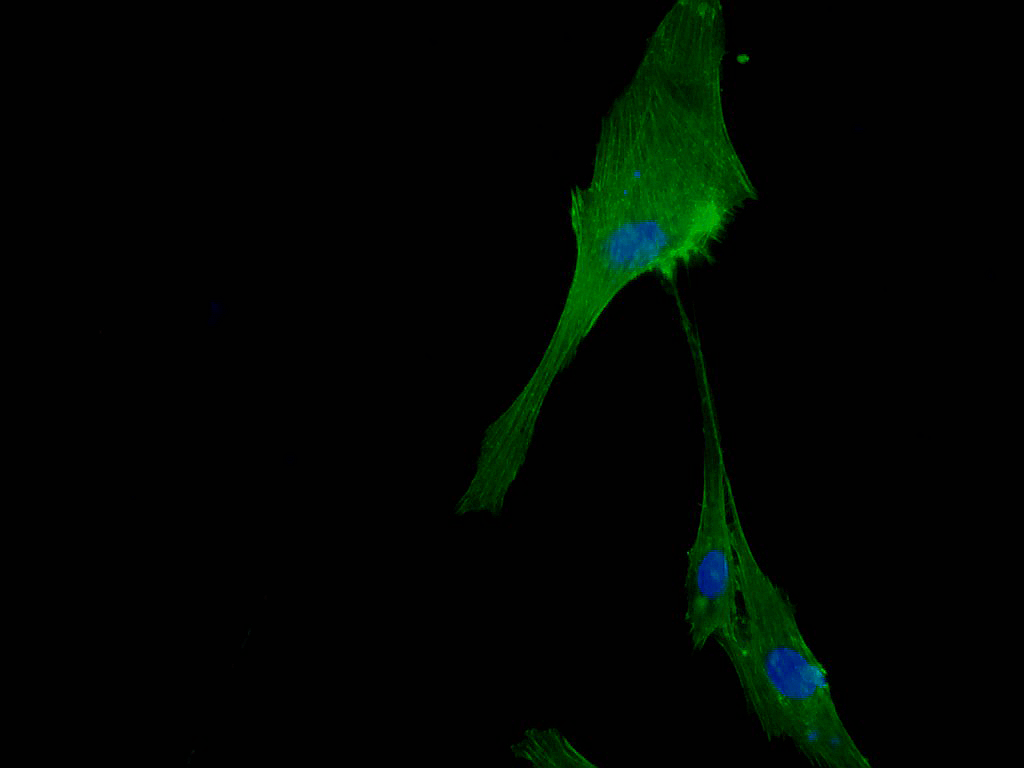
**

**Untreated cell**

**
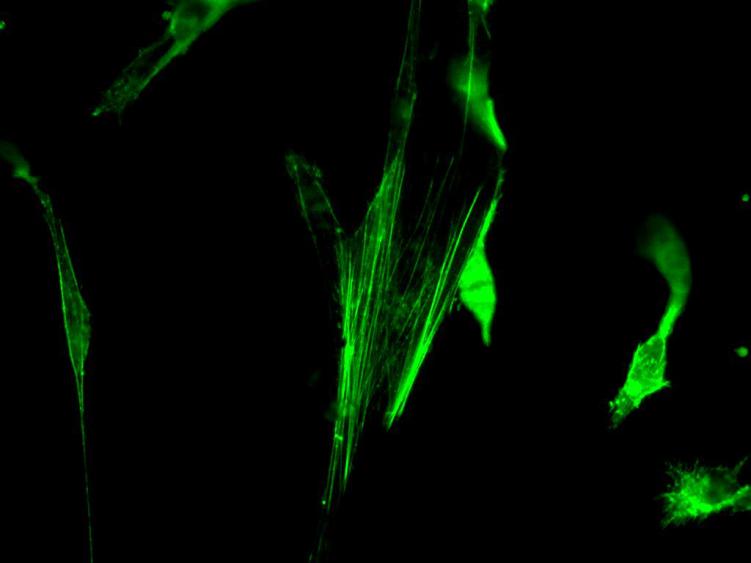

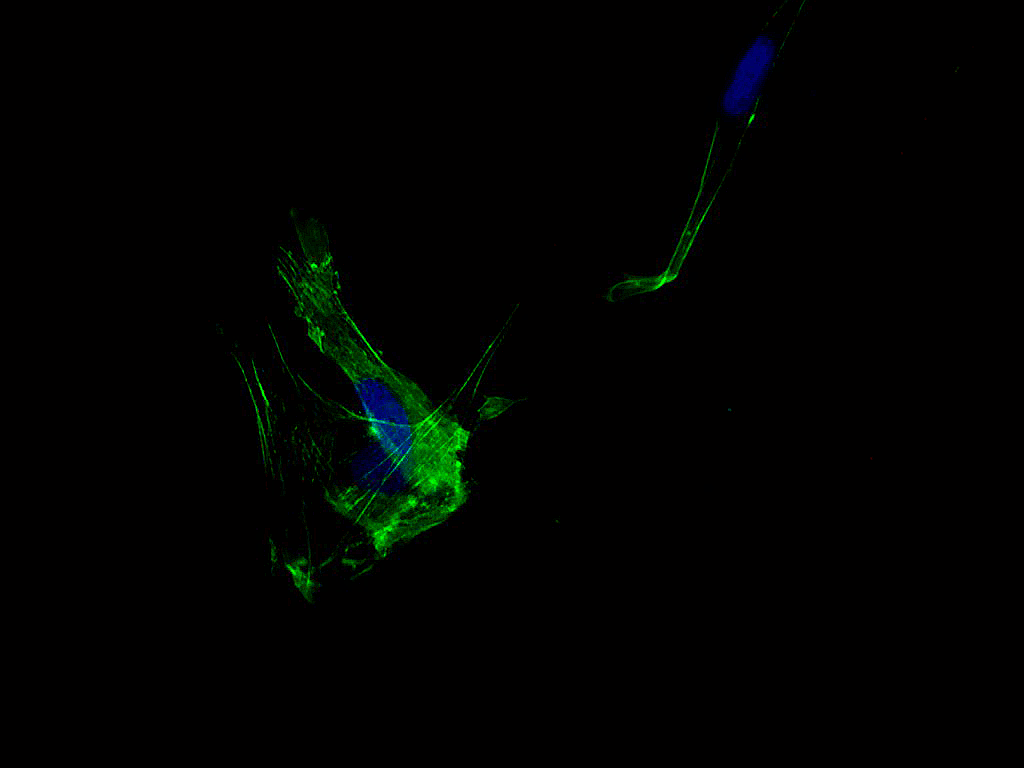

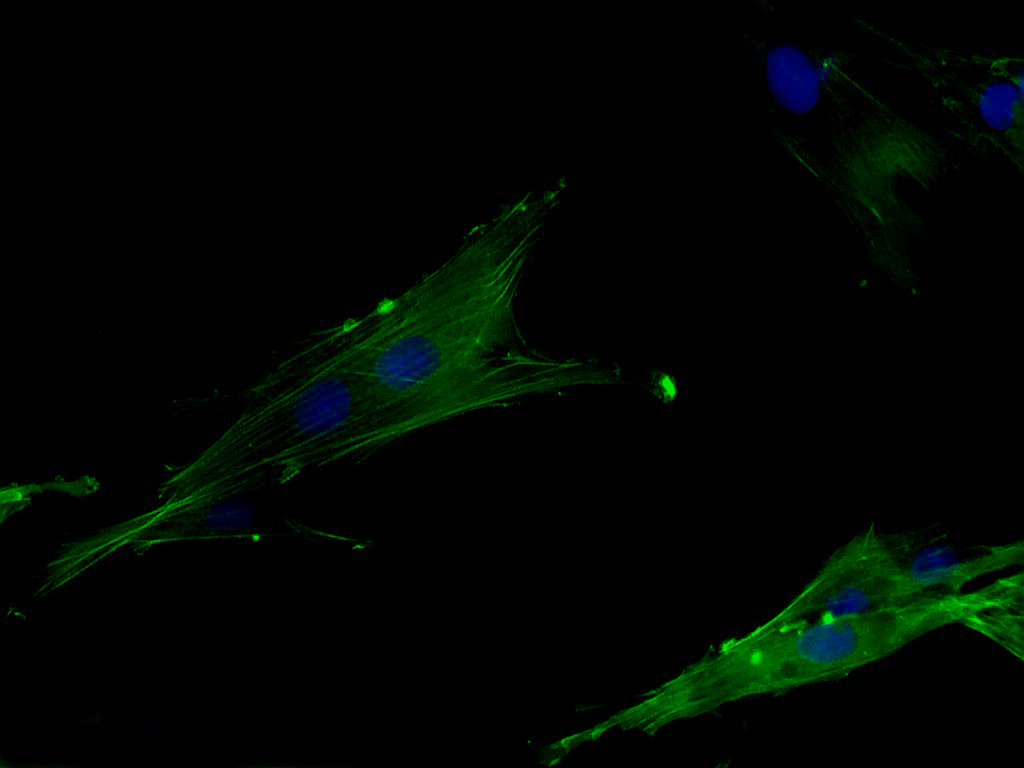
**

**PDGF+FA(200ng/ml)**

**
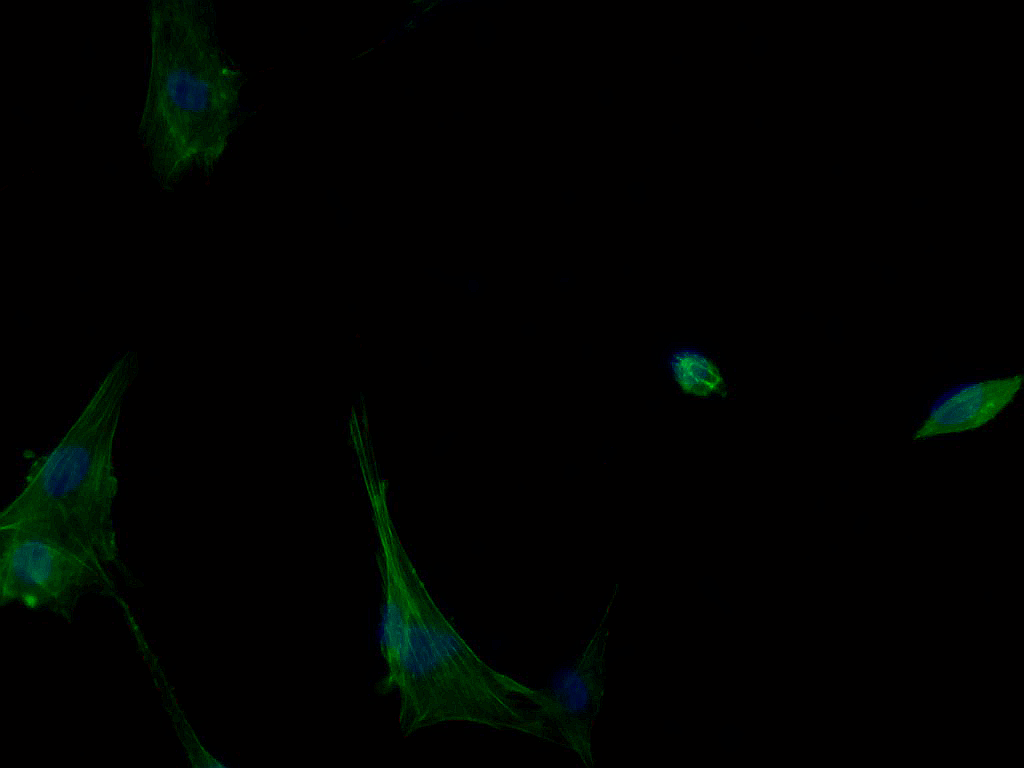

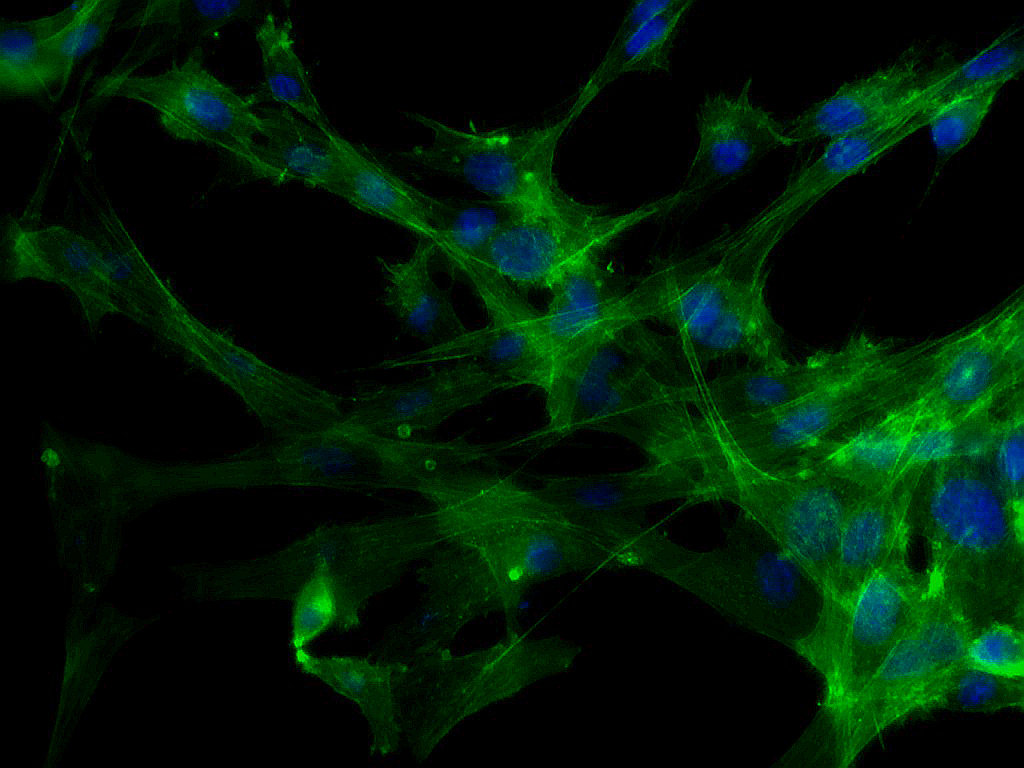

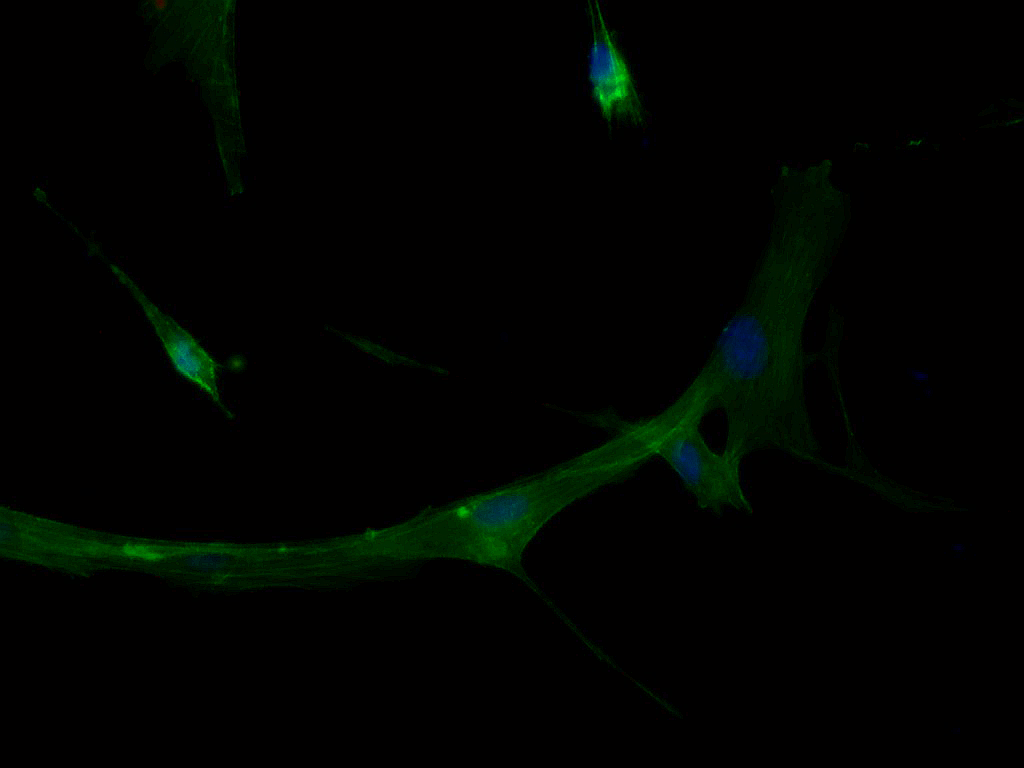
**

**PDGF+FA(400ng/ml)**

**
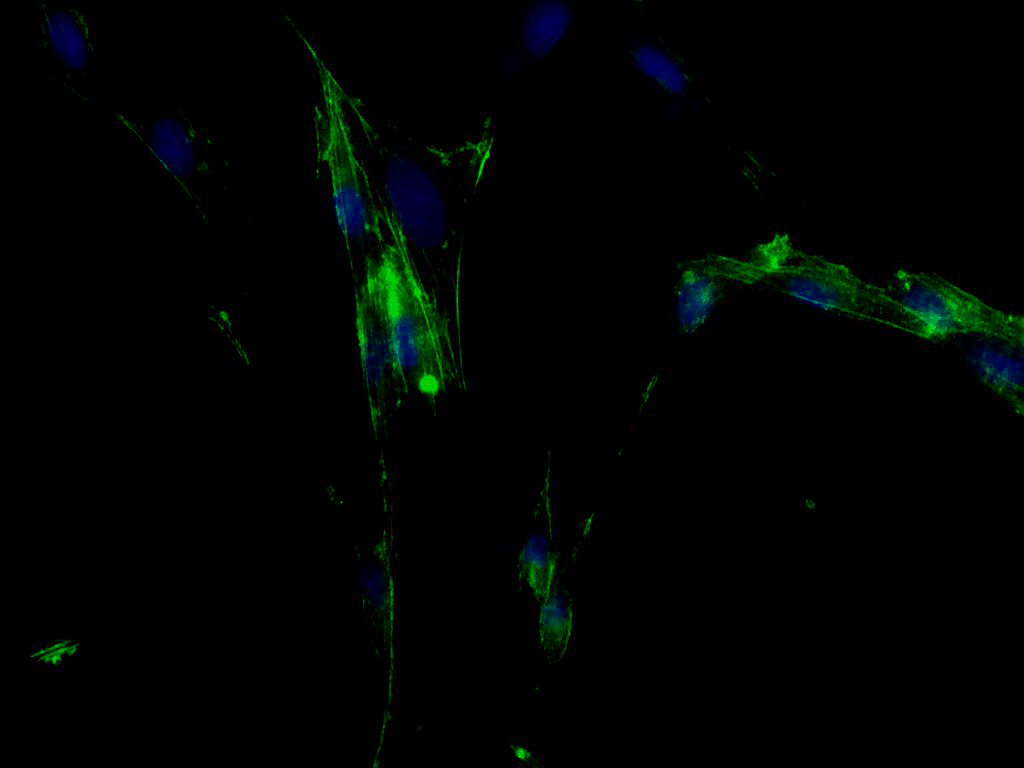

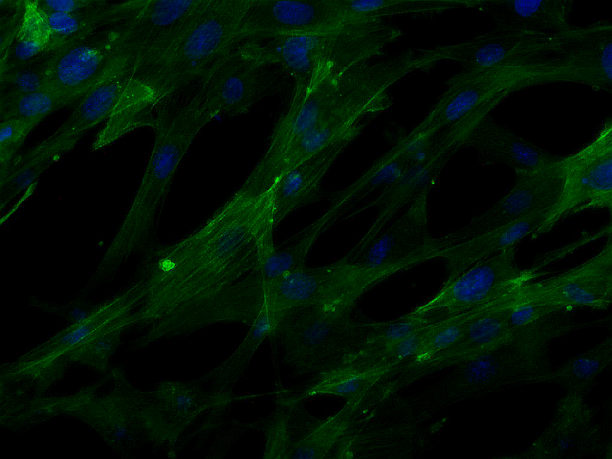

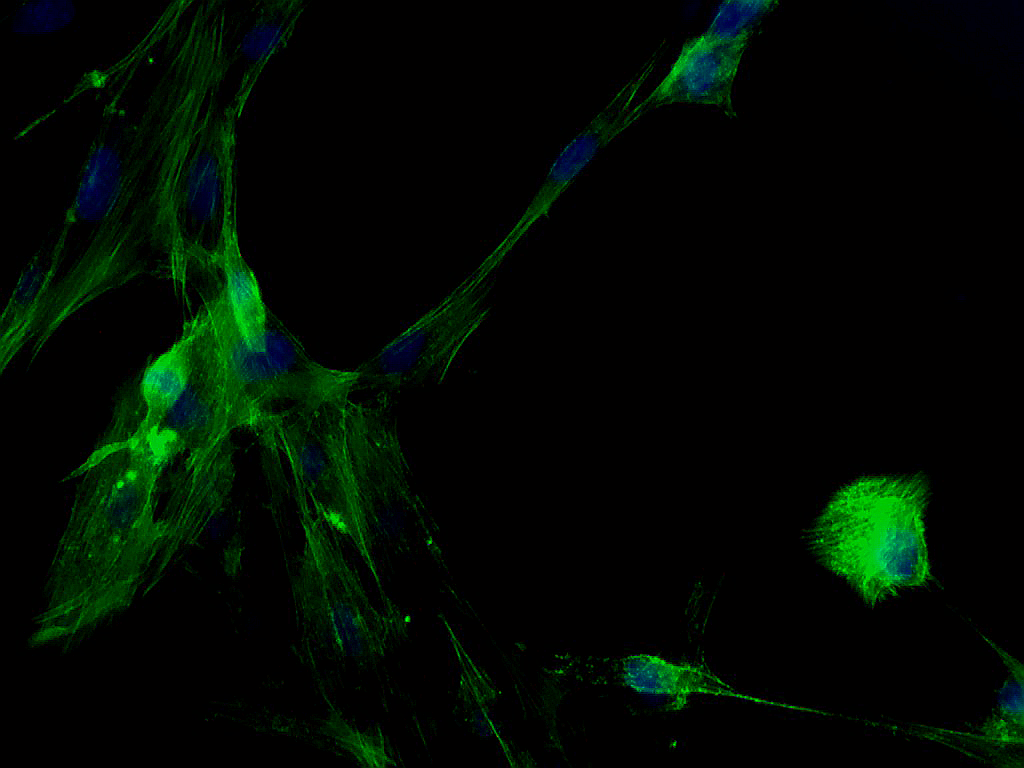
**
