# Supplementary material for: Ferulic Acid Alleviates Atherosclerotic Plaques by Inhibiting VSMC Proliferation Through the NO/p21 Signaling pathway
Source: J Cardiovasc Transl Res. 2022 Jan 6;15(4):865–75. doi: 10.1007/s12265-021-10196-8 (PMC9622559; doi:10.1007/s12265-021-10196-8)

**Figure 6**

| HFD | HFD+Simva | HFD+FA |
| --- | --- | --- |
| 1.13 | 1.513 | 2.805 |
| 1.013 | 2.012 | 1.231 |
| 2.524 | 2.011 | 2.011 |
| 4.312 | 1.958 | 1.932 |
| 1.013 | 3.012 | 3.121 |
| 0.901 | 2.413 | 1.012 |
| 1.542 | 0.045 | 0.521 |
| 0.312 | 0.923 | 1.21 |

**
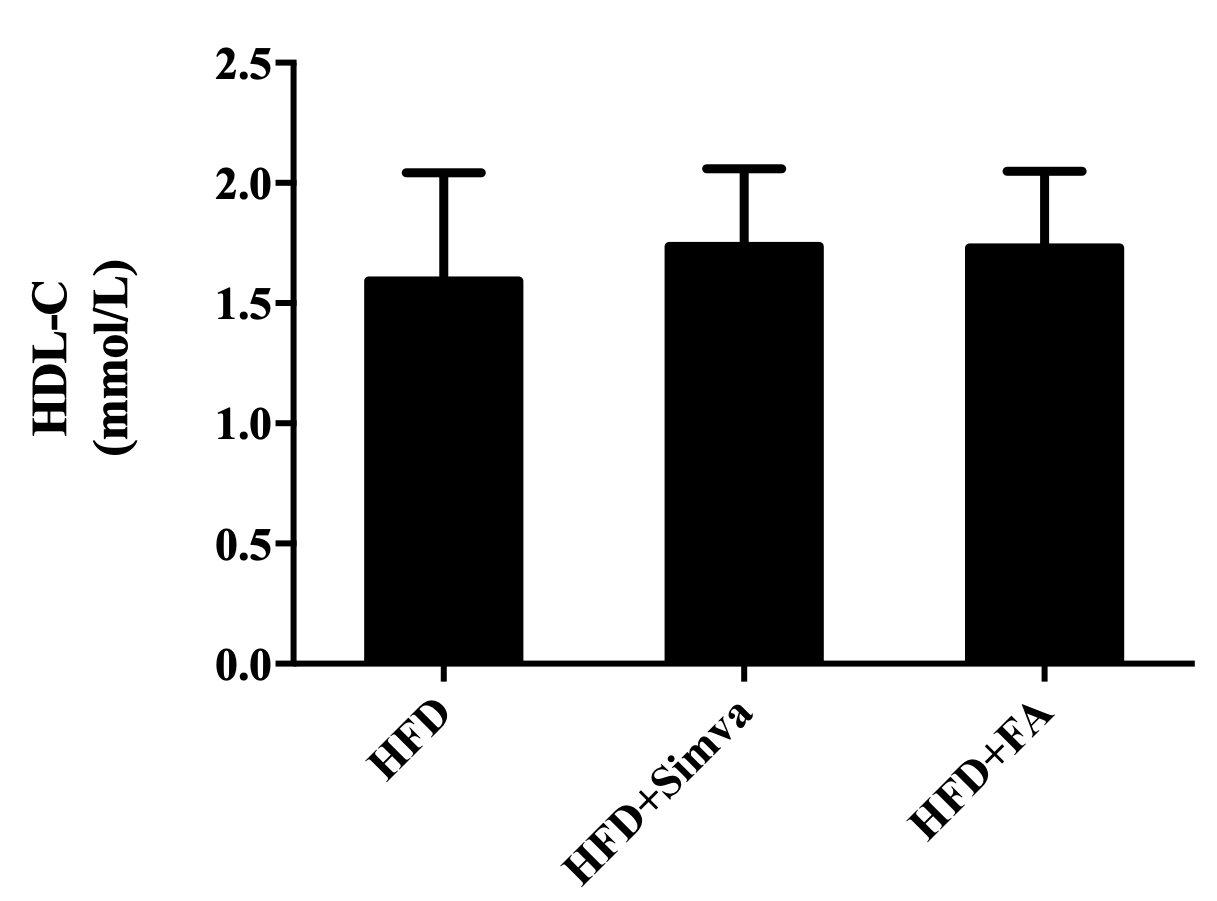
**

| HFD | HFD+Simva | HFD+FA |
| --- | --- | --- |
| 5.445 | 3.089 | 3.493 |
| 4.913 | 3.897 | 3.012 |
| 5.891 | 3.701 | 4.891 |
| 8.089 | 4.624 | 2.037 |
| 6.542 | 1.378 | 0.102 |
| 2.237 | 4.052 | 2.781 |
| 5.087 | 0.672 | 4.021 |
| 6.871 | 2.891 | 4.589 |

**
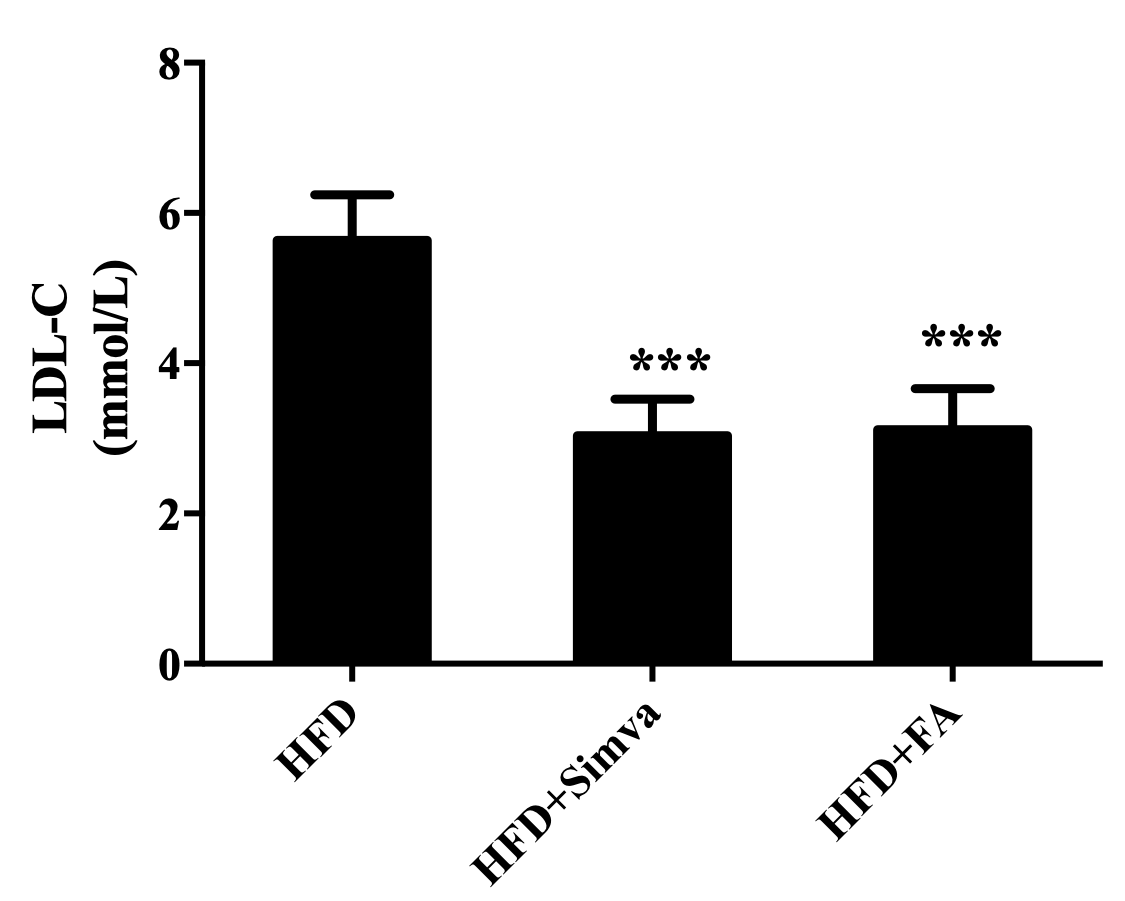
**

| HFD | HFD+Simva | HFD+FA |
| --- | --- | --- |
| 15.23 | 7.43 | 11.52 |
| 13.48 | 8.23 | 12.87 |
| 16.21 | 12.99 | 13.09 |
| 14.57 | 9.64 | 9.11 |
| 15.57 | 9.79 | 10.81 |
| 15.02 | 11.23 | 18.79 |
| 18.29 | 8.13 | 11.2 |
| 14.68 | 4.33 | 10.38 |

**
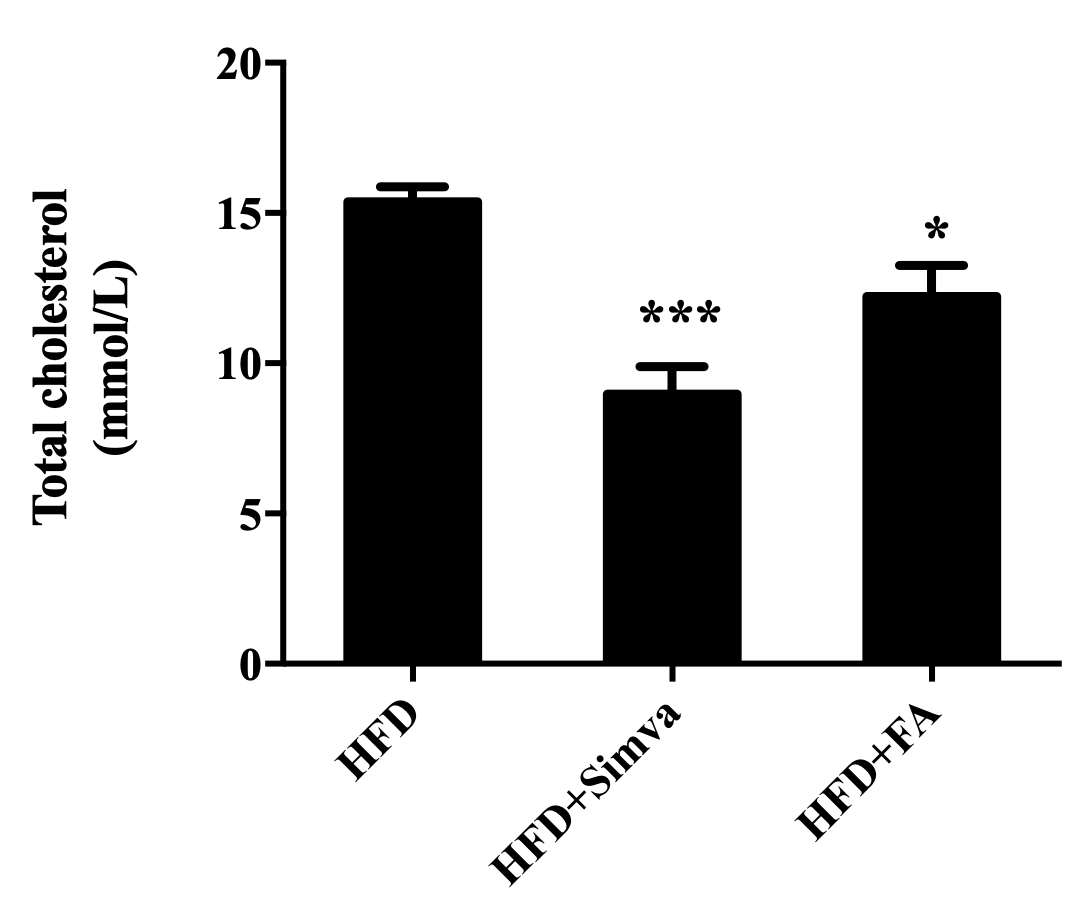
**

| HFD | HFD+Simva | HFD+FA |
| --- | --- | --- |
| 1.68 | 0.423 | 0.831 |
| 2.323 | 0.831 | 1.212 |
| 0.792 | 0.725 | 0.793 |
| 1.321 | 0.312 | 0.542 |
| 1.453 | 0.456 | 0.942 |
| 2.574 | 0.334 | 0.81 |
| 1.562 | 0.512 | 1.021 |
| 1.793 | 0.437 | 0.987 |

**
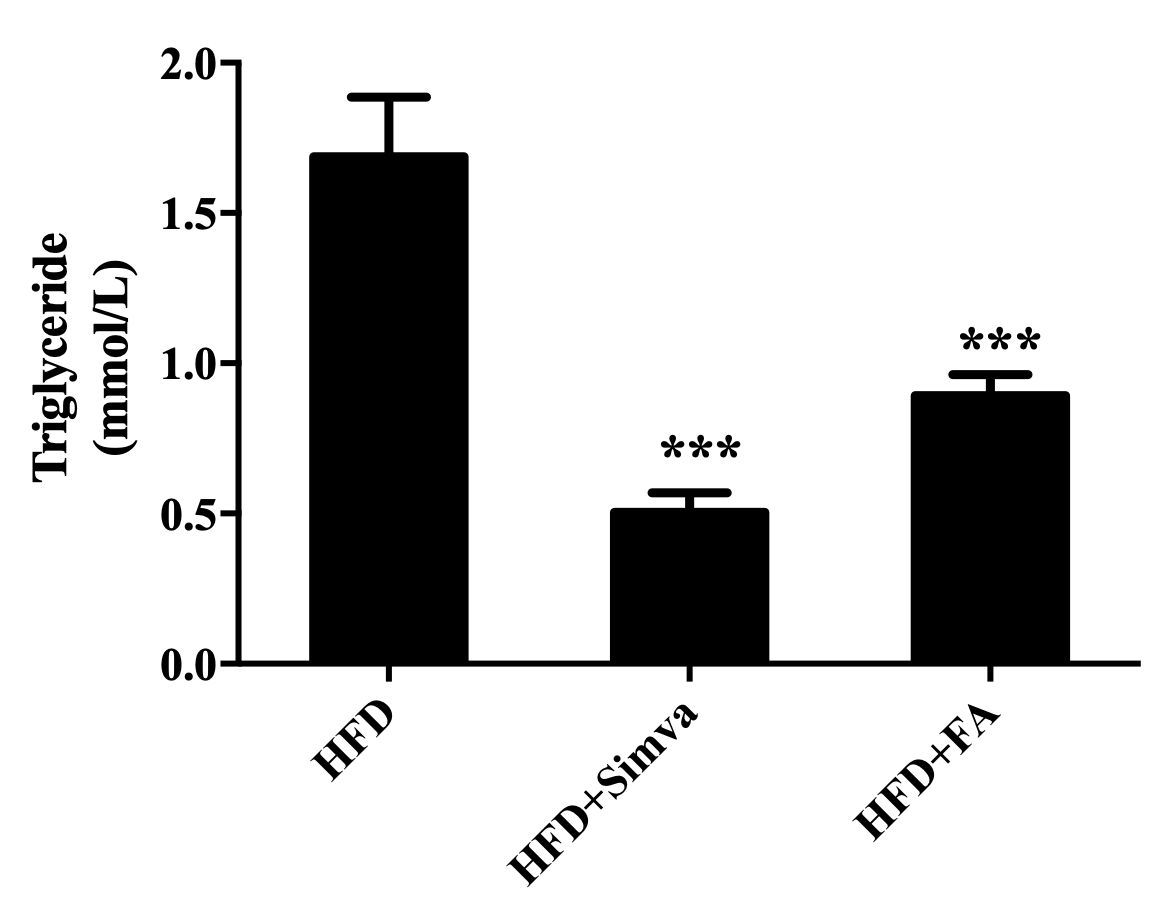
**

**HFD**


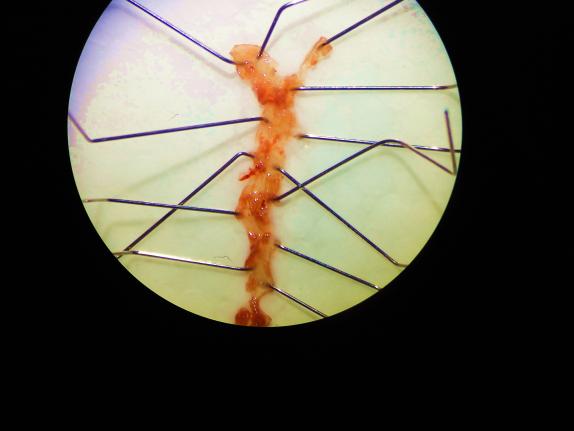

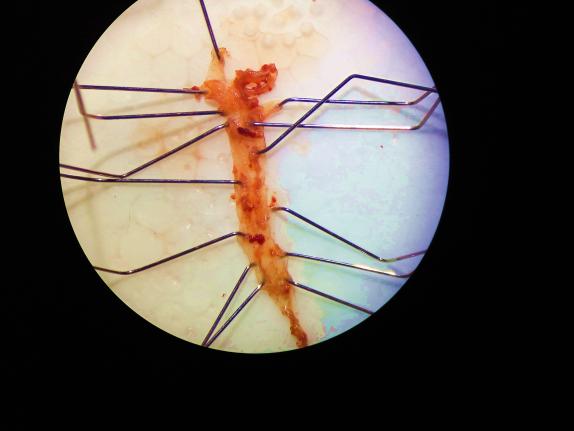


**
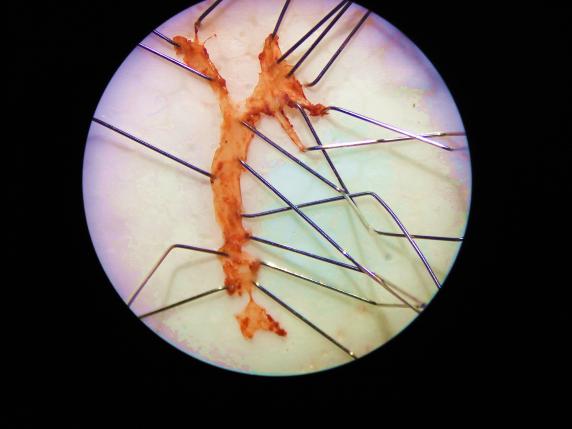

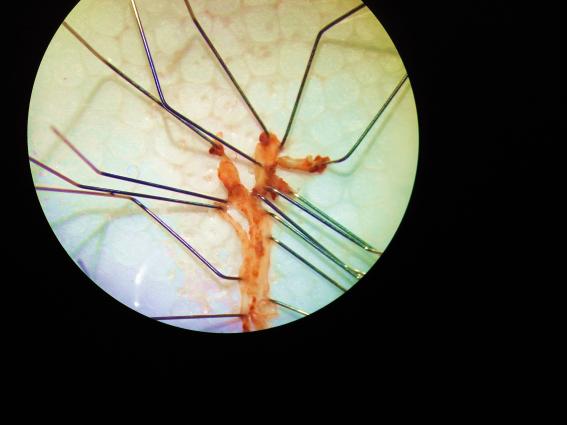
**

**
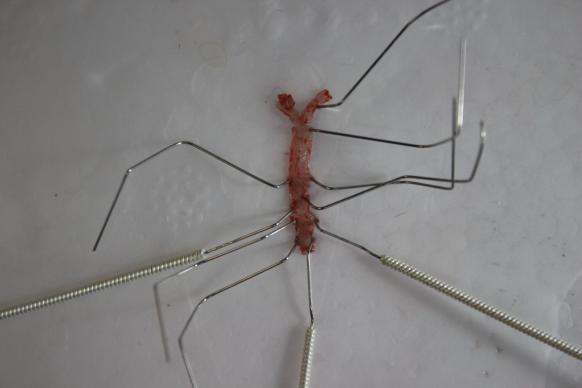
**

**
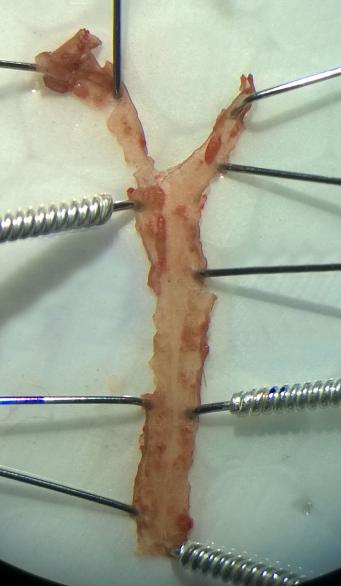
**

**HFD+FA**


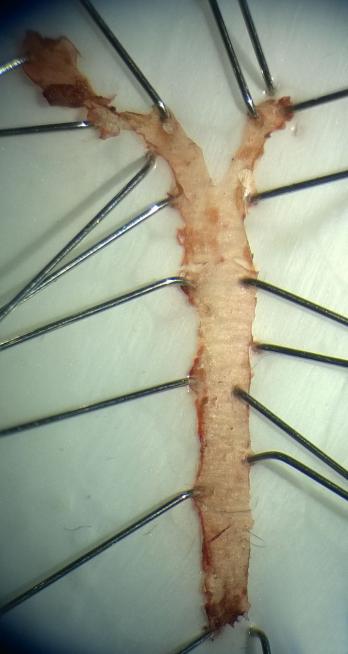

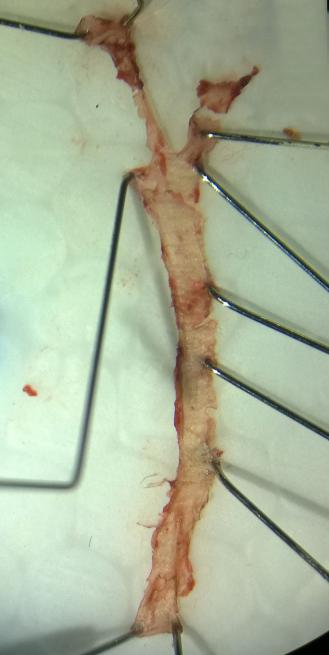

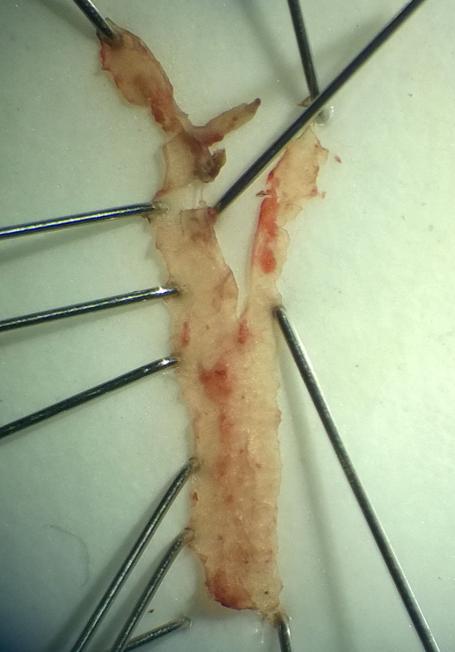

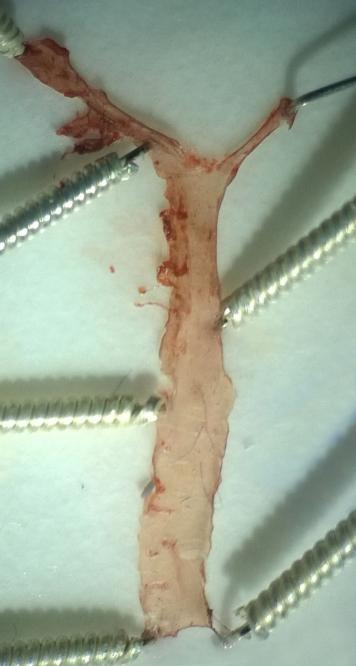

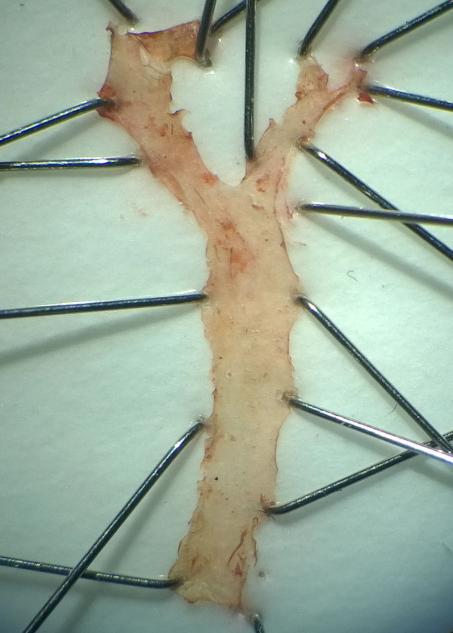

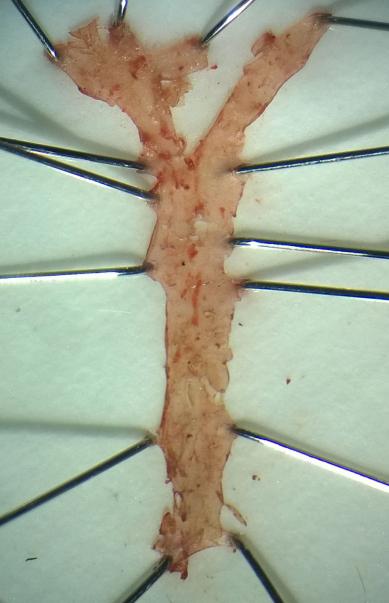


**HFD+Simva**


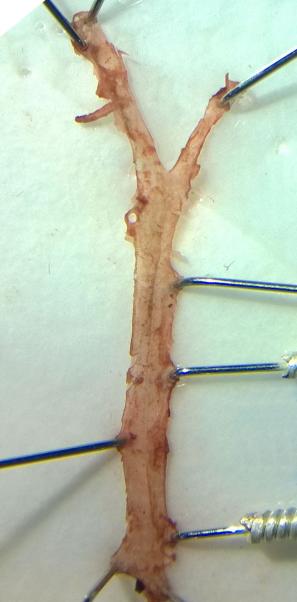

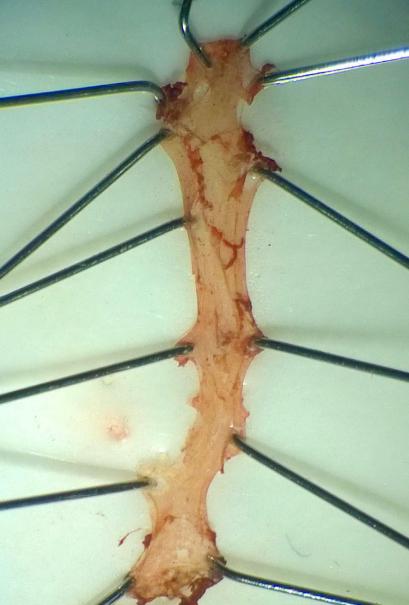

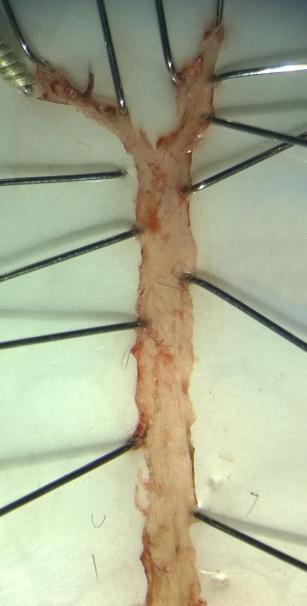

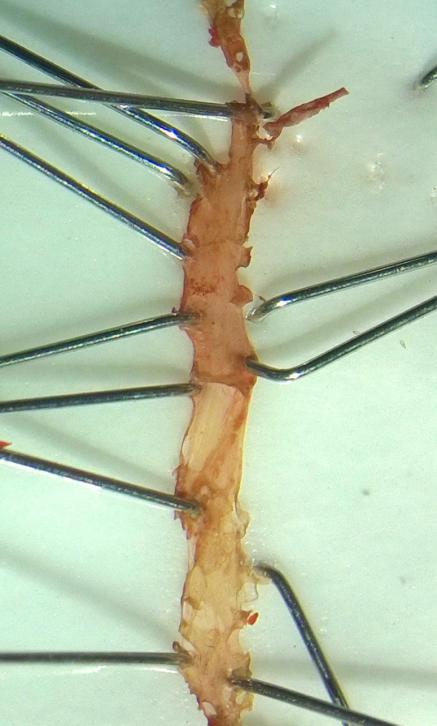

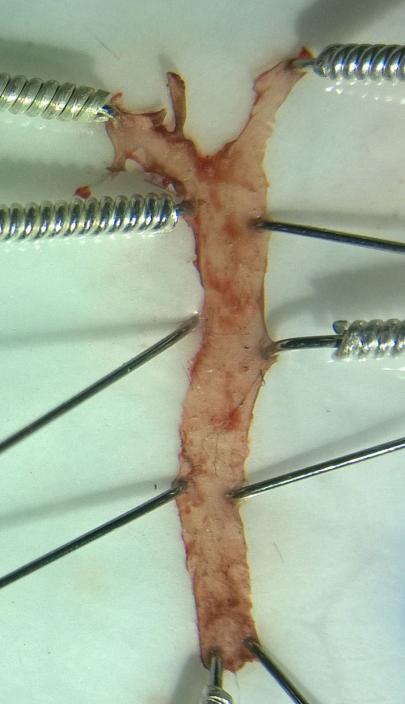

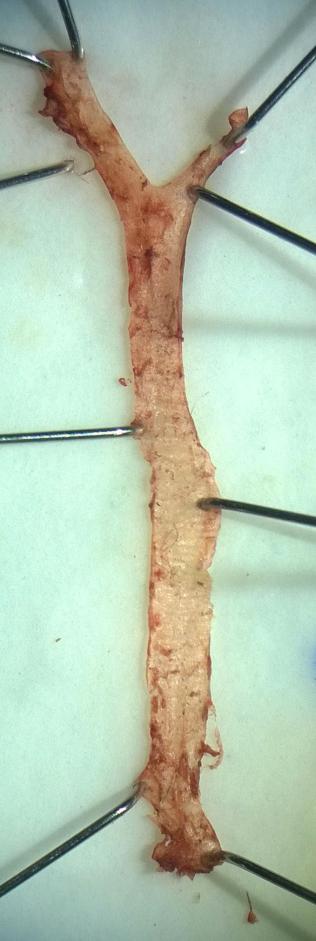


**HFD**


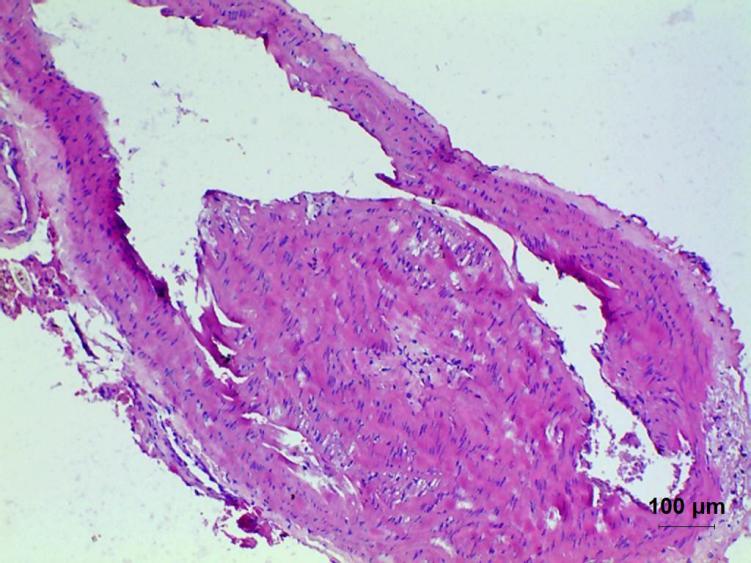

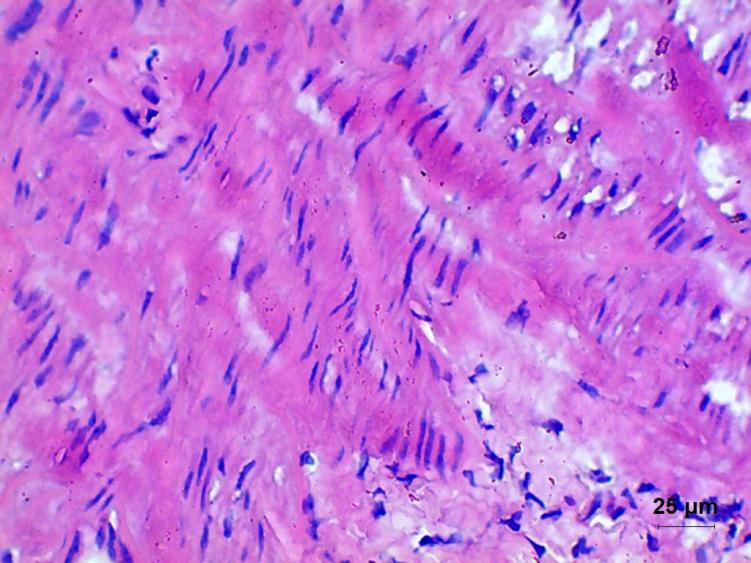

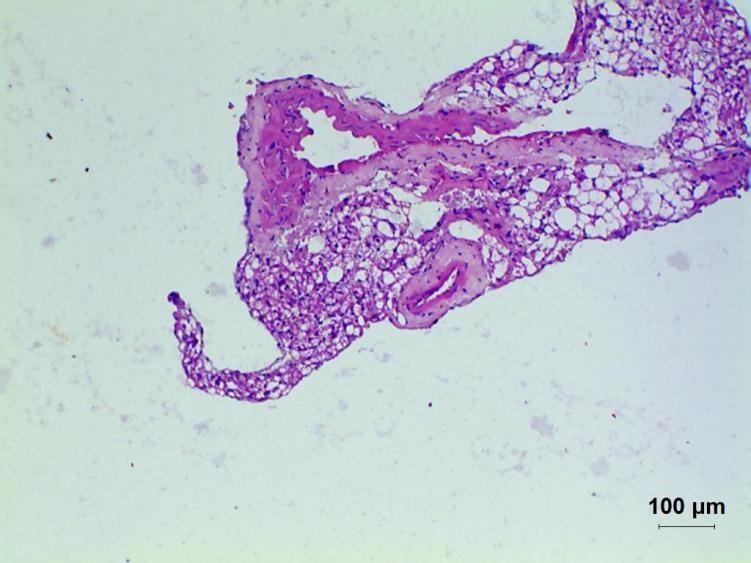

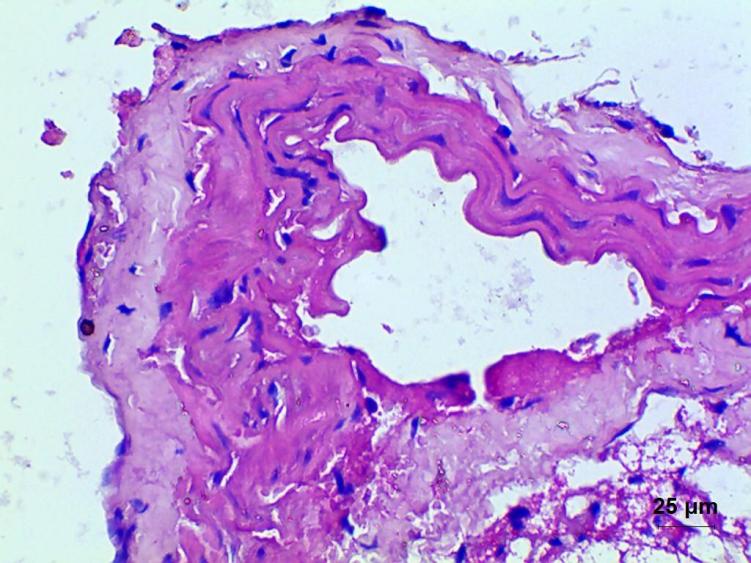

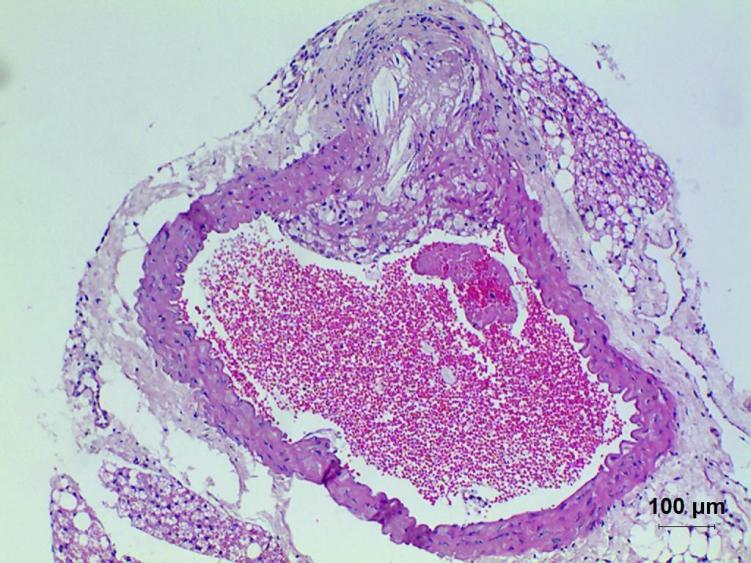

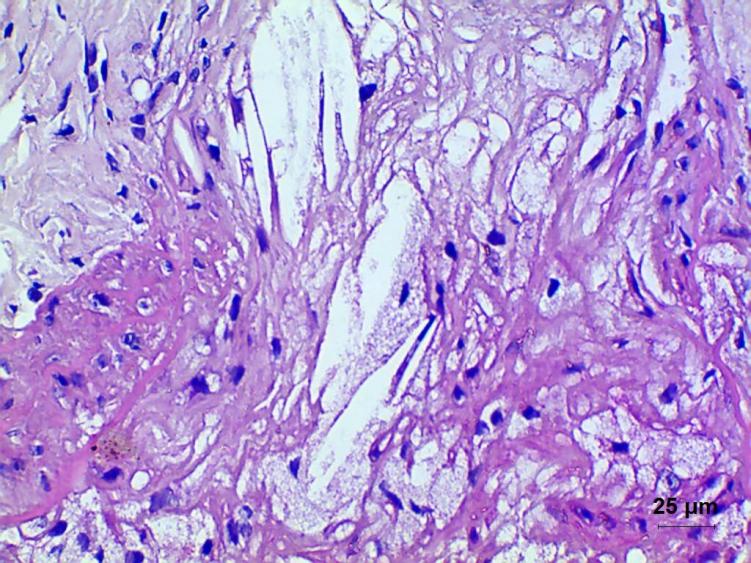

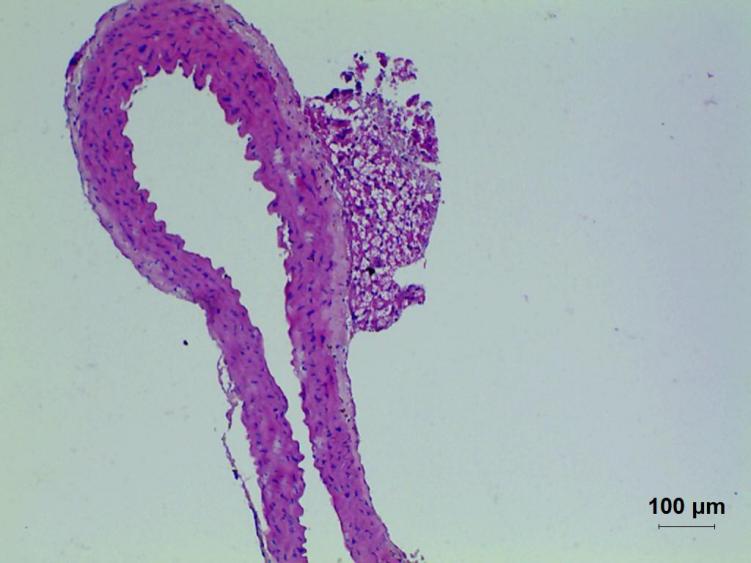

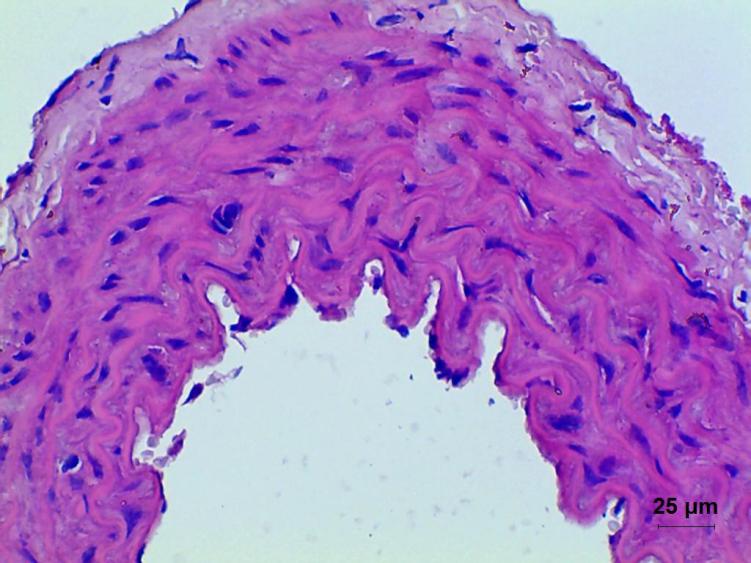

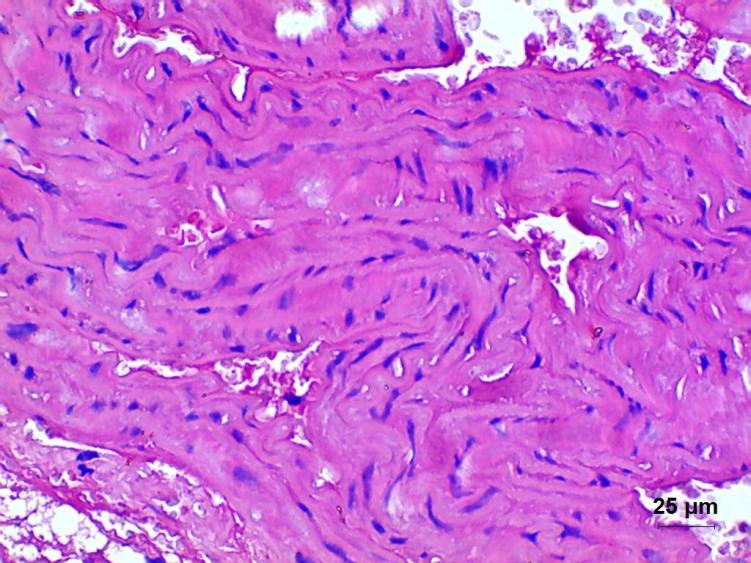

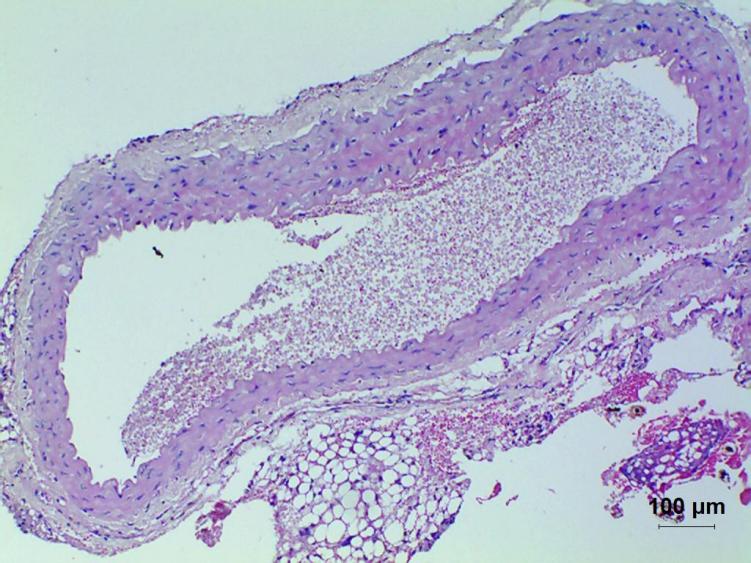

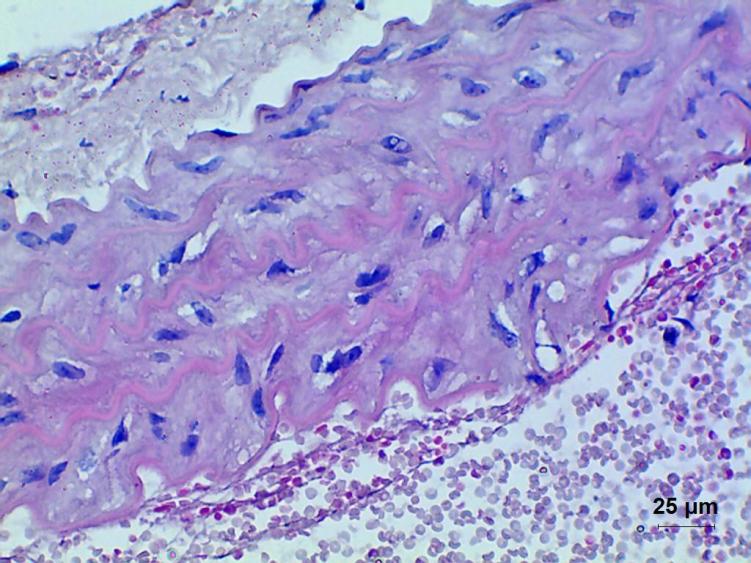


**HFD+FA**

**
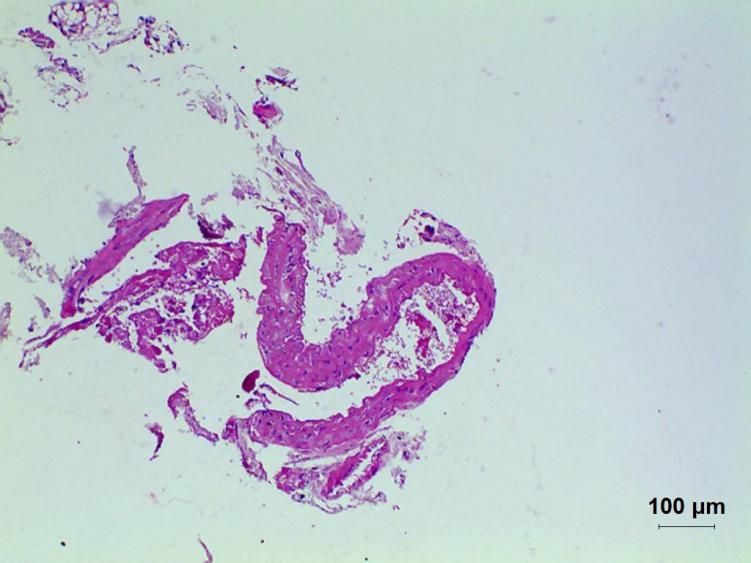

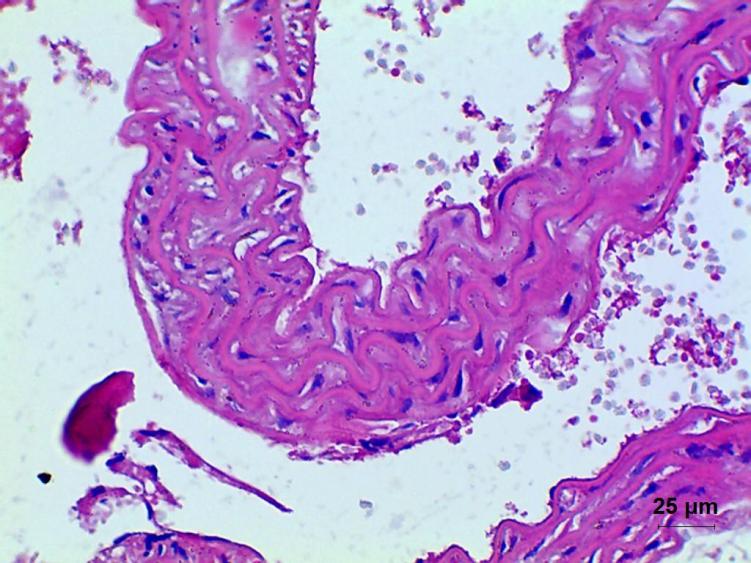

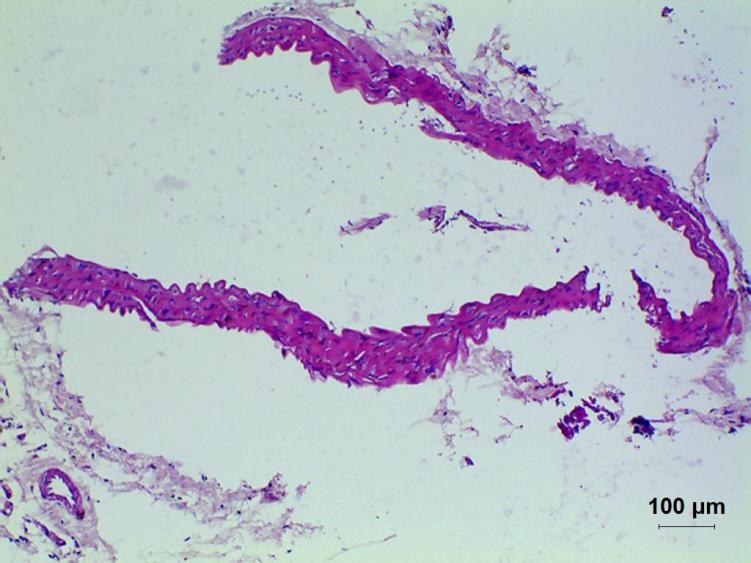

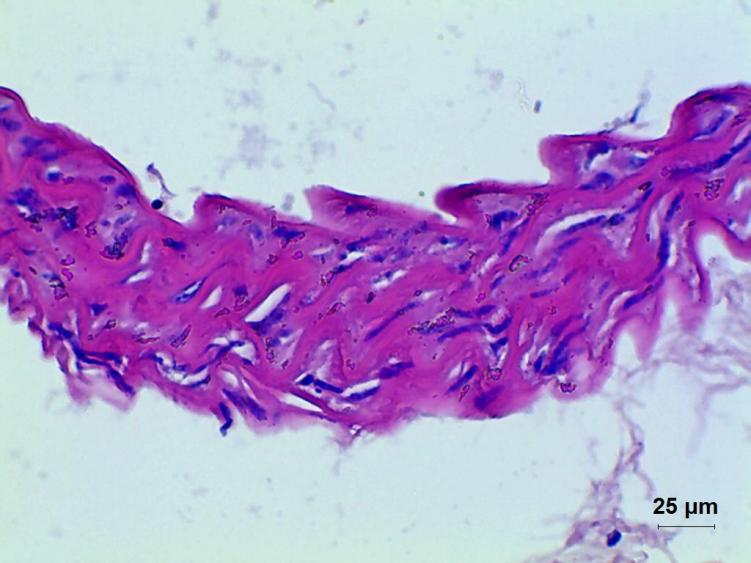

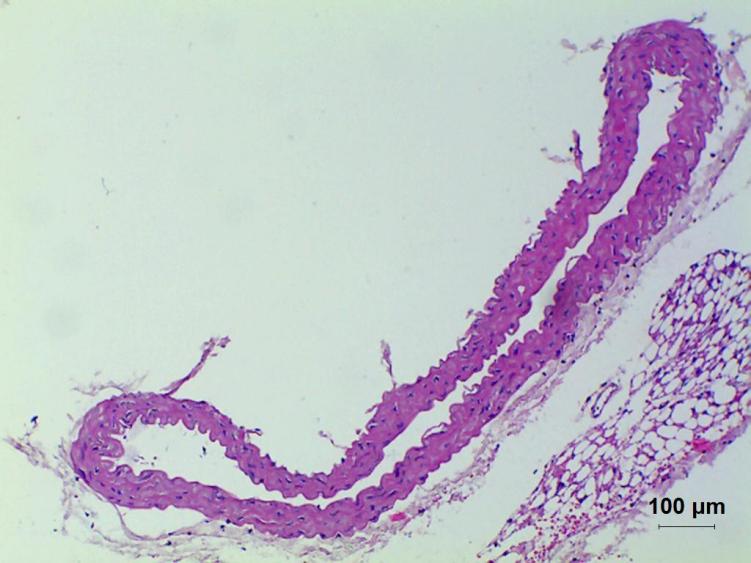

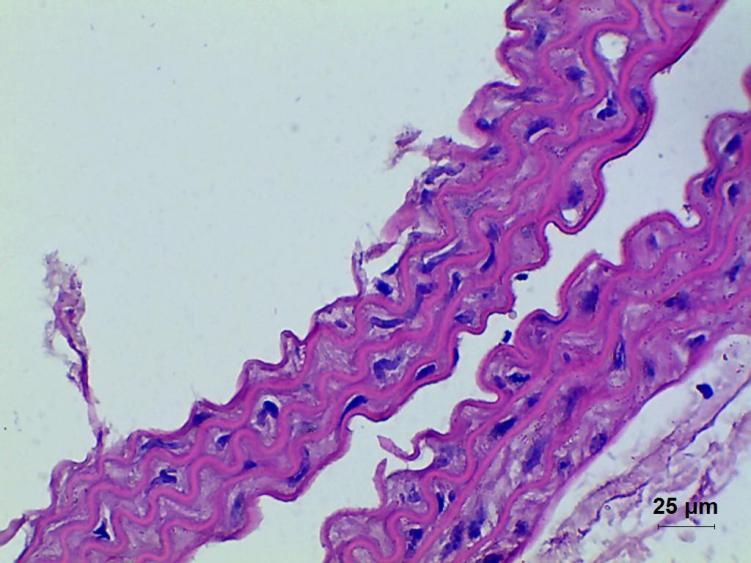

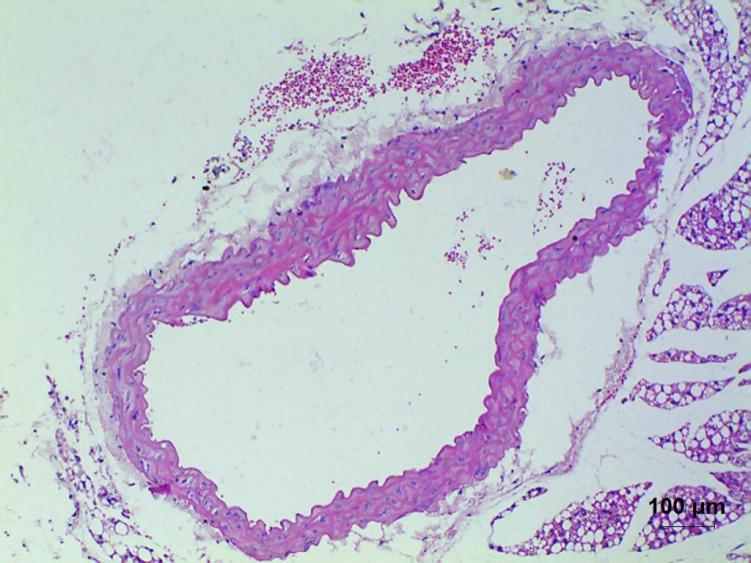

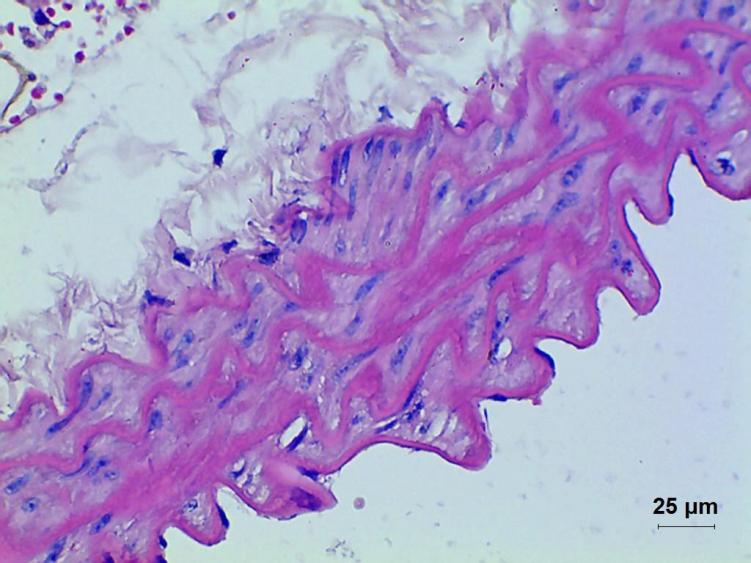

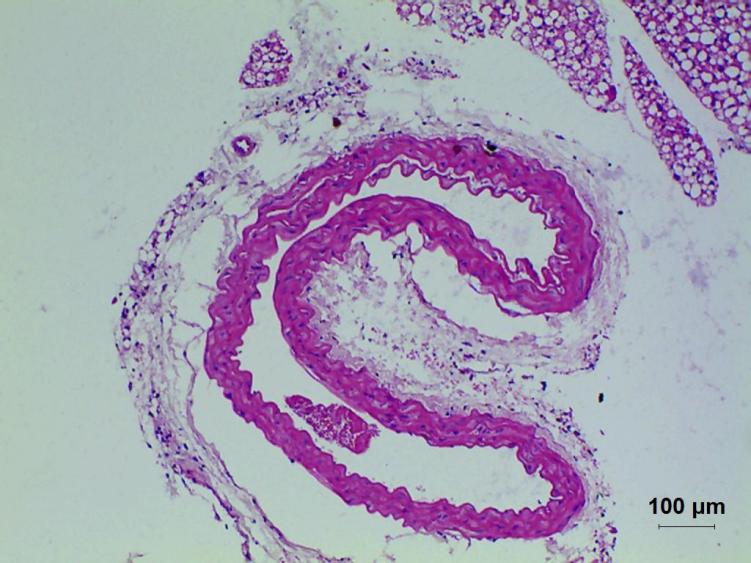

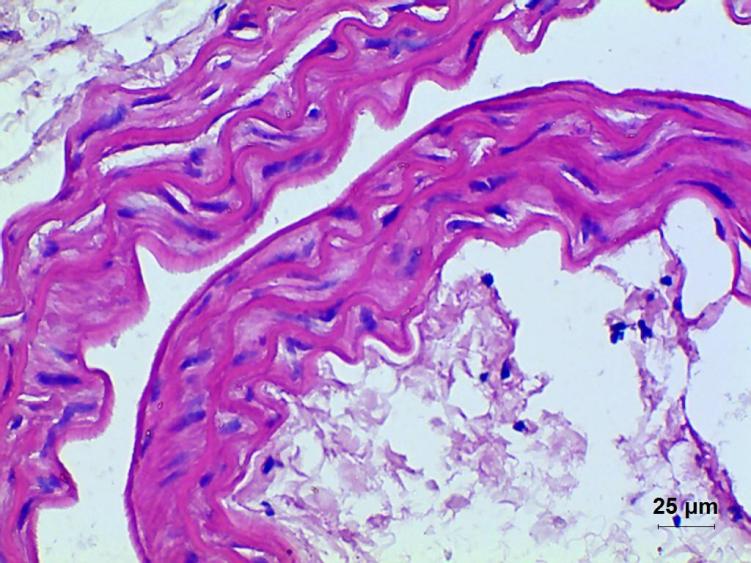

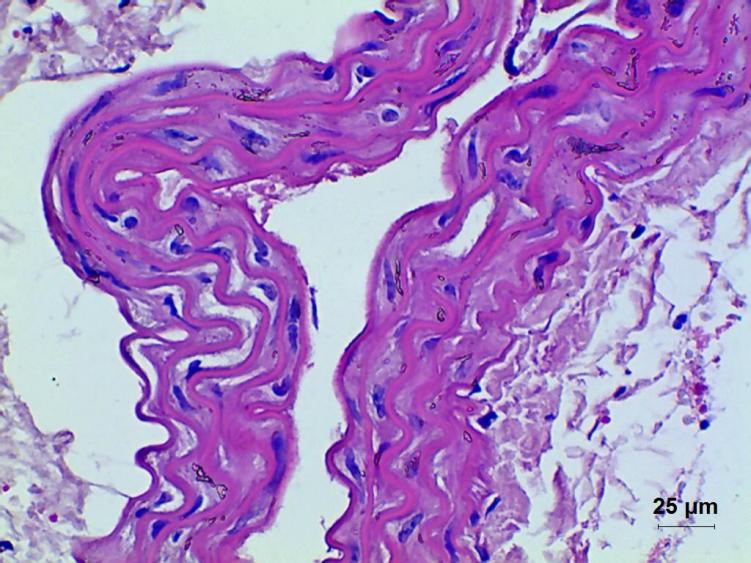
**

**HFD+Simva**


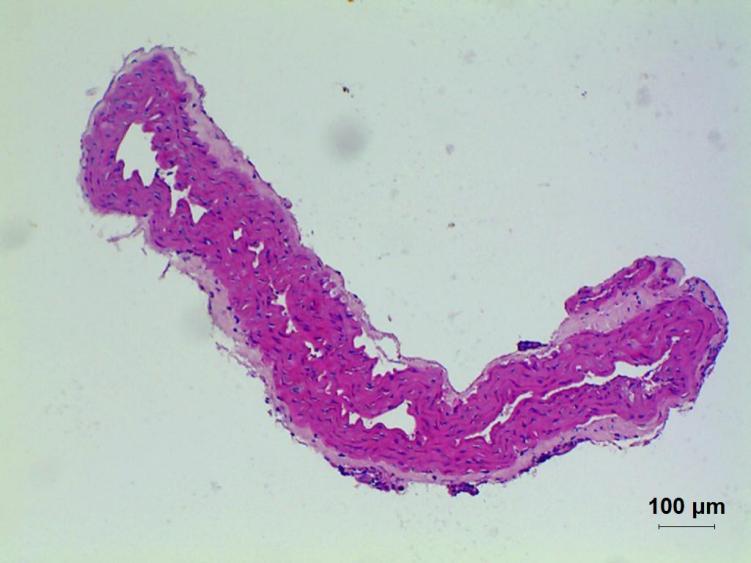

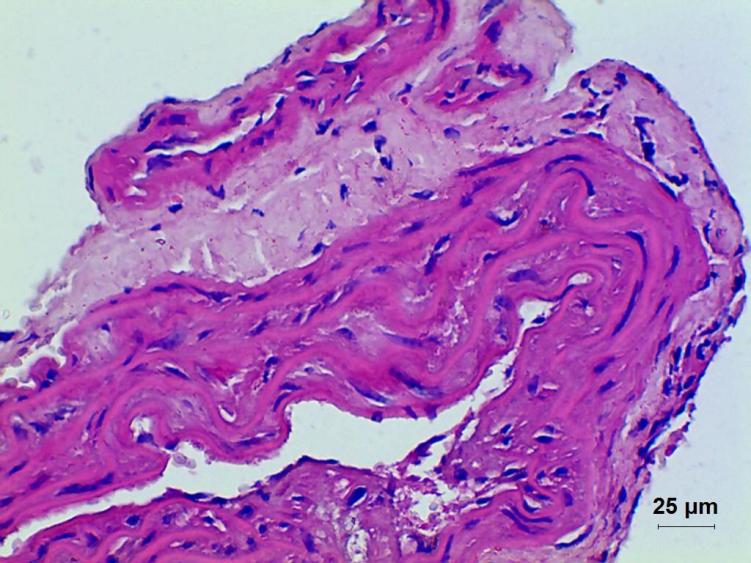

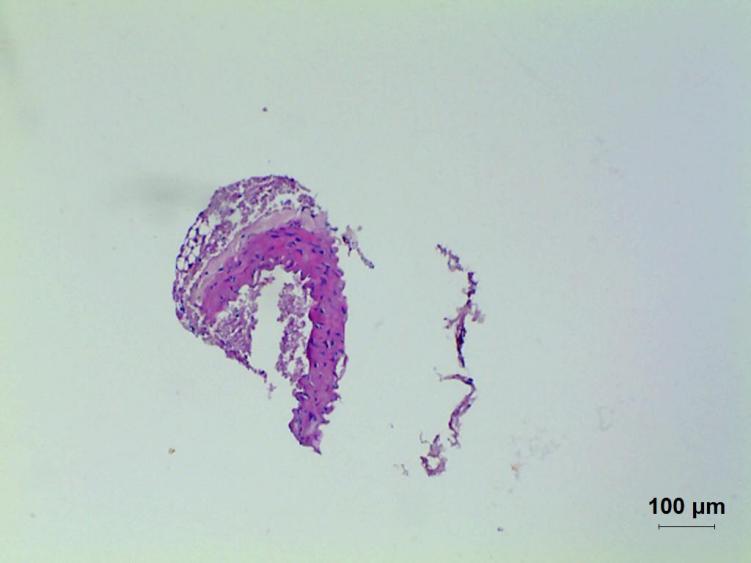

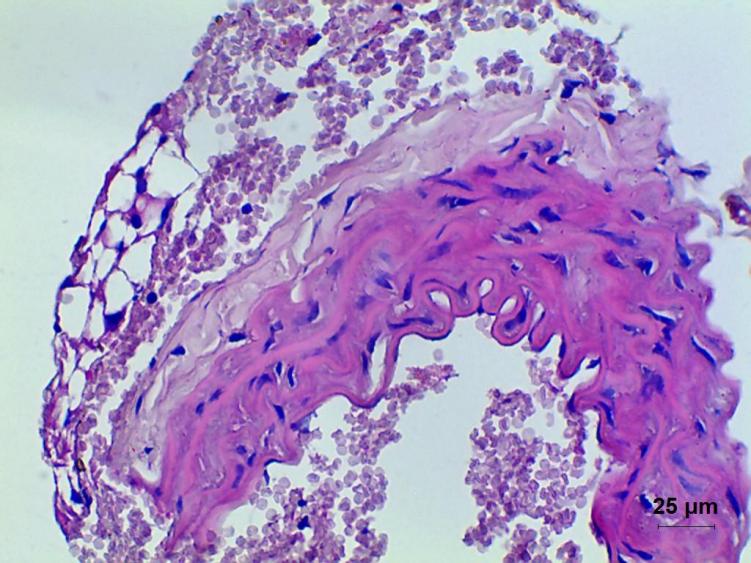

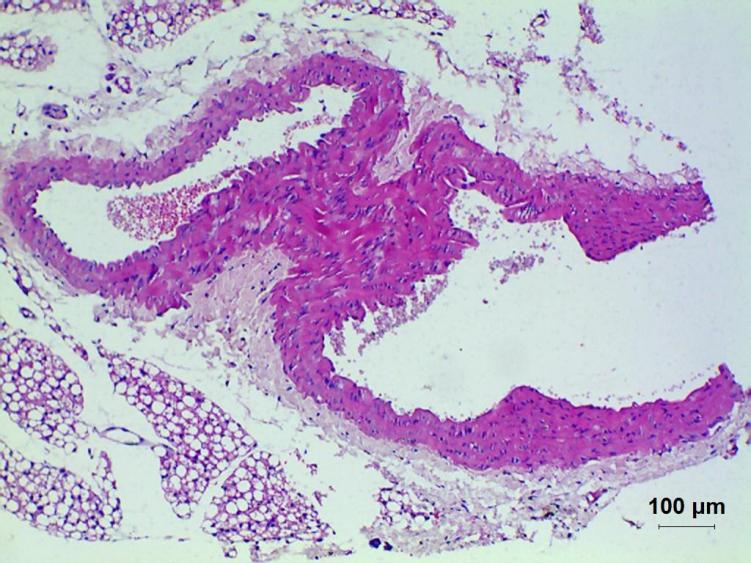

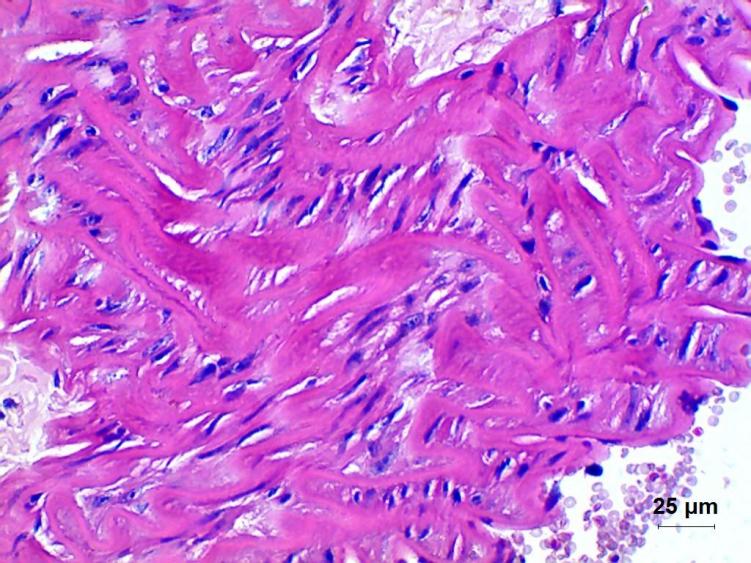

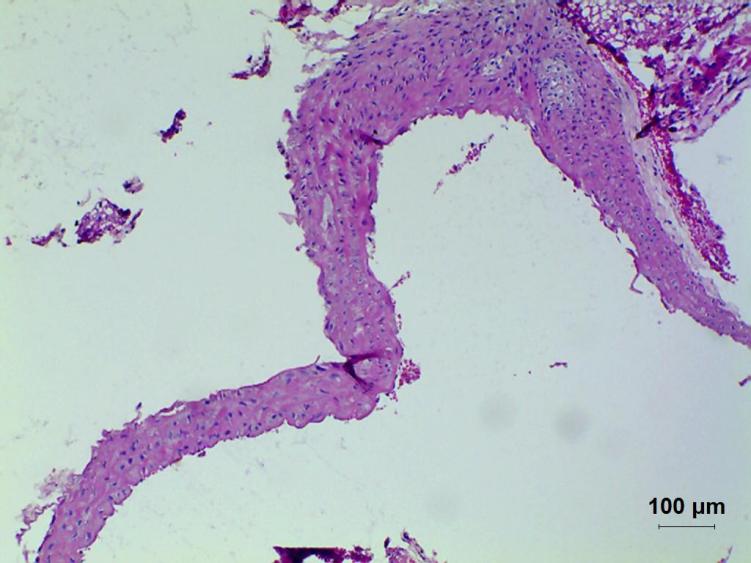

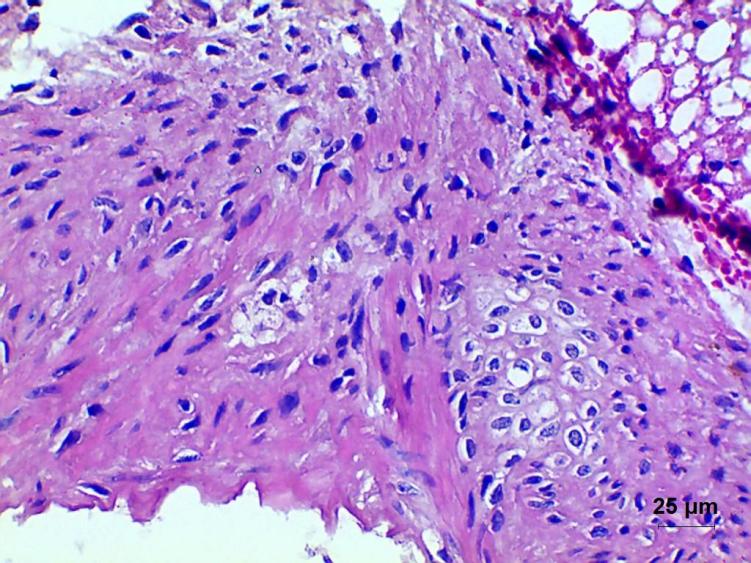

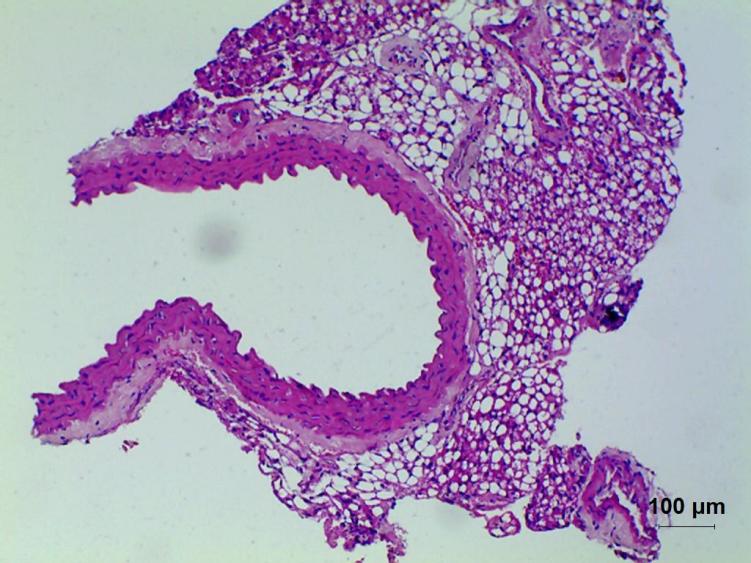

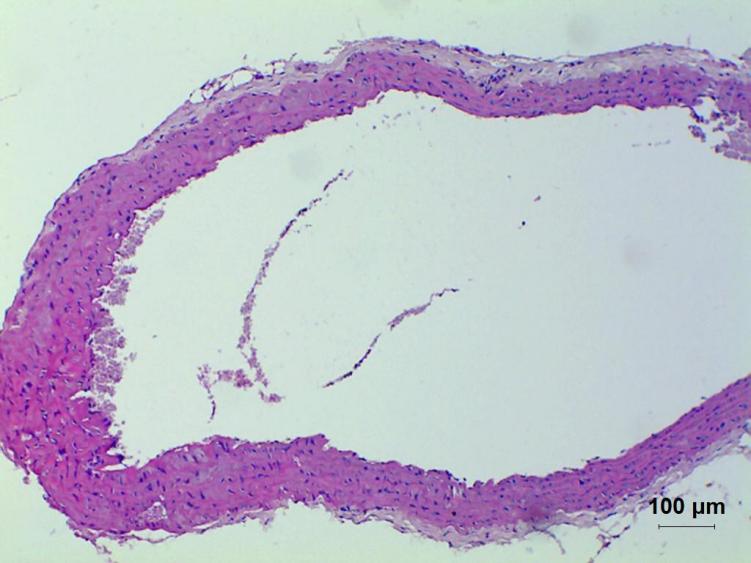

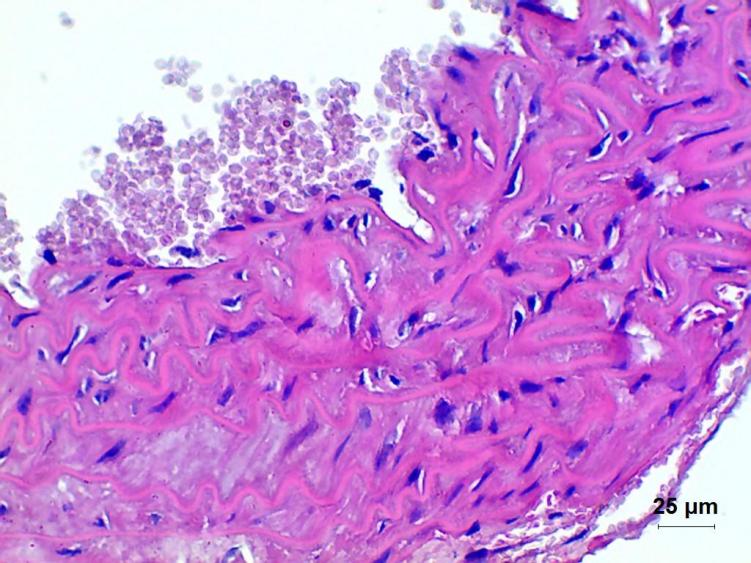

Supplement: Supplementary file 6 — Supplementary file6 (DOCX 2895 KB) [file 12265_2021_10196_MOESM6_ESM.docx]
